# Triplet‐Sensitized Switching of High‐Energy‐Density Norbornadienes for Molecular Solar Thermal Energy Storage with Visible Light

**DOI:** 10.1002/anie.202414733

**Published:** 2024-11-02

**Authors:** Till J. B. Zähringer, Nico Perez Lopez, Robin Schulte, Matthias Schmitz, Heiko Ihmels, Christoph Kerzig

**Affiliations:** ^1^ Department of Chemistry Johannes Gutenberg University Mainz Duesbergweg 10–14 55128 Mainz Germany; ^2^ Department of Chemistry-Biology and Center of Micro- and Nanochemistry and (Bio)Technology (Cμ) University of Siegen Adolf-Reichwein-Str. 2 57068 Siegen Germany

**Keywords:** Energy Transfer, MOST, Solar Energy Conversion, Sustainable Chemistry, Time-Resolved Spectroscopy

## Abstract

Norbornadiene‐based photoswitches have emerged as promising candidates for harnessing and storing solar energy, holding great promise as a viable solution to meet the growing energy demands. Despite their potential, the effectiveness of their direct photochemical conversion into the resulting quadricyclanes has room for improvement owing to (i) moderate quantum yields, (ii) poor overlap with the solar spectrum and (iii) photochemical back reactions. Herein, we present an approach to enhance the performance of such molecular solar thermal energy storage (MOST) systems through the triplet‐sensitized conversion of aryl‐substituted norbornadienes. Our study combines deep spectroscopic analyses, irradiation experiments, and quantum mechanical calculations to elucidate the energy transfer mechanism and inherent advantages of the resulting MOST systems. We demonstrate remarkable quantum yields using readily available sensitizers under both LED and solar light irradiation, significantly surpassing those achieved through direct excitation with photons of higher energy. In contrast to the conventional approach, light‐induced back reactions of the high‐energy products do not play any role, allowing quantitative switching within minutes. These results not only underscore the potential of triplet‐sensitized MOST systems to leverage the high energy storage capabilities of multistate photoswitches but they might also stimulate the broader usage of sensitization strategies in photochemical energy conversion.

## Graphical abstract

A highly efficient energy storage system using underexplored sensitization strategies has been developed. Quantitative conversion and maximum isomerization quantum yields of aryl‐substituted norbornadienes to the stable quadricyclane counterpart with exceptional energy storage capabilities are achieved, while harvesting photons from the solar spectrum efficiently.

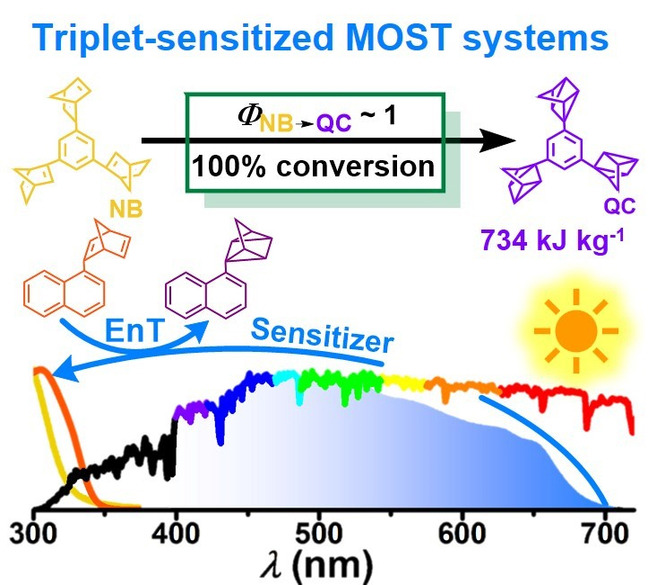

## Introduction

The unpredictability and non‐continuity of solar energy reaching the Earth's surface cannot yet be compensated by reliable energy storage systems. Promising methods of conserving solar energy are achieved by initially converting it to electrical energy and storing it as mechanical, electrochemical, or chemical energy.^[1]^ In this context, so‐called molecular solar thermal energy storage (MOST) systems that directly capture solar energy and release thermal energy on demand attracted much interest.[^2^, ^3^, ^4^, ^5^, ^6^] MOST systems are based on molecular photoswitches[^7^, ^8^, ^9^] that upon excitation store energy through reversible chemical bond formation or isomerization processes. Among them, norbornadiene‐, azobenzene‐, and dihydroazulene‐based structures are the most extensively studied systems.[^6^, ^10^, ^11^] Already more than 30 years ago, Yoshida and Bren’ et al. defined essential criteria that must be met by molecular systems for efficiently storing solar energy.[^12^, ^13^] Briefly,

1. absorptivity in the visible spectral range,
2. energy storage capabilities above 300 kJ/kg,
3. prolonged conservation of the stored energy by the photoisomer,
4. high quantum yield of the photochemical forward isomerization and a high efficiency of the backward reaction upon thermal or catalytic initiation,
5. readily available and cheap chromophores with sufficient stability.

**Figure 1:**
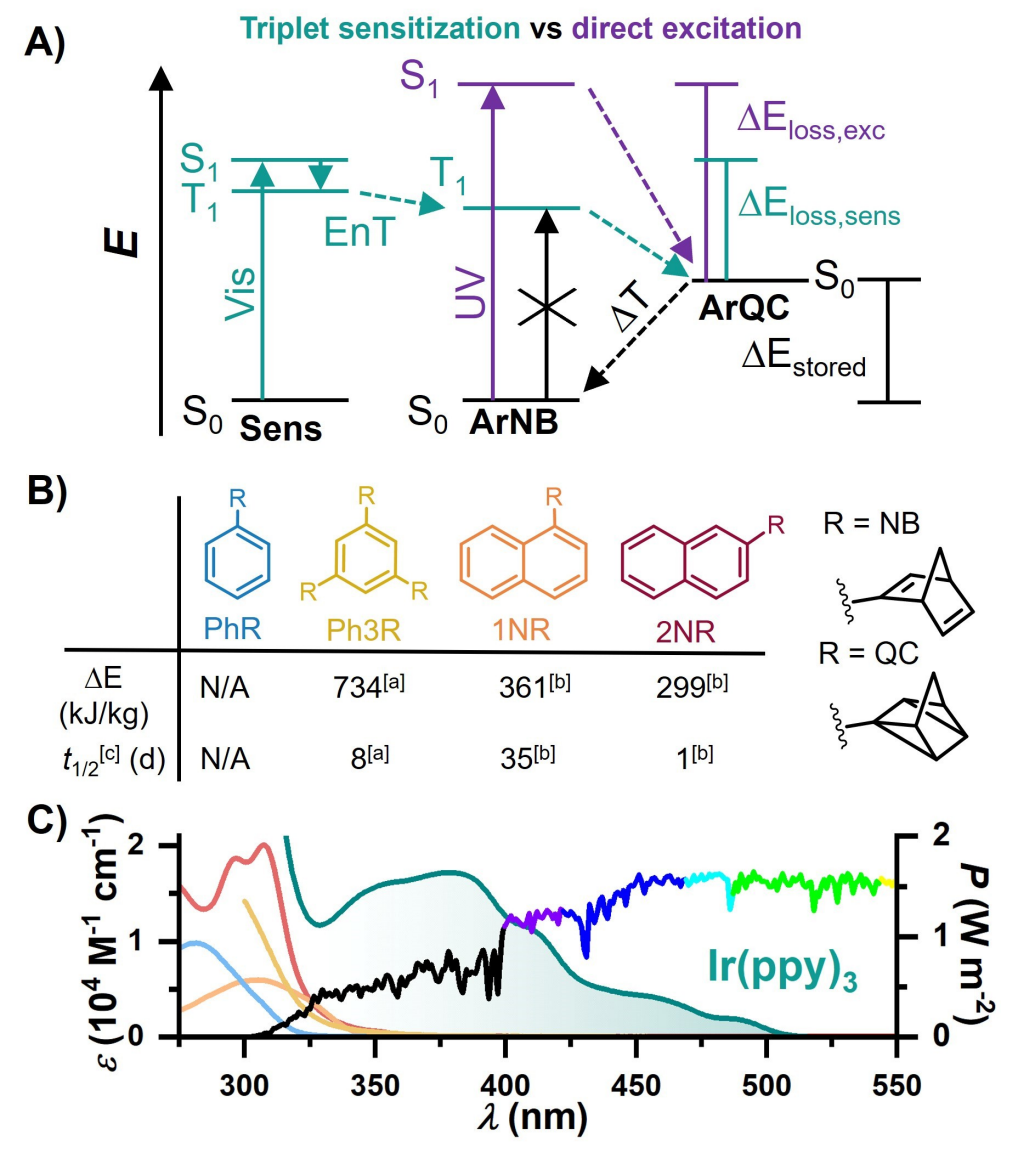
A) Energy diagram of the isomerization of aryl‐substituted norbornadienes (ArNB) to the respective quadricyclane (ArQC) following direct excitation in comparison to triplet sensitization. B) Molecular structures of aryl‐substituted norbornadiene photoswitches. [a] Taken from Ref [43] [b] Taken from Ref [44], along with key properties. [c] Reaction enthalpy for cycloreversion and thermal half‐life of the ArQC calculated at 25 °C. C) Color‐coded absorption spectra of the norbornadienes and a prototypical sensitizer (green line, see below for its structure and properties) overlaid by the terrestrial solar irradiation spectrum.^[45]^

The photoinduced [2+2] cycloaddition of norbornadiene (NB) has long been recognized as a promising reaction for energy storage applications.[^12^, ^14^] This is due to its high energy storage capability (~1000 kJ/kg)^[15]^ and the sufficient half‐life^[16]^ of the photoisomer quadricyclane (QC). Direct excitation of NB is, however, not practical due to the poor solar spectral overlap (Figure S28) and the low conversion quantum yield ($\Phi_{NB\to QC}$ =0.03–0.05).[^17^, ^18^] With these challenges in mind, novel NB structures exhibiting pull‐push conjugation at one NB double bond, steric hindrance as well as multistate photoswitches were developed.[^13^, ^19^, ^20^, ^21^, ^22^, ^23^] These modifications successfully improve key properties such as solar spectral match, energy storage density and/or thermal half‐life. Nonetheless, it is important to note that improvements in one characteristic often entail a compromise with other attributes.^[19]^ In stark contrast to direct excitation forming the high energy S_1_ state, isomerization reactions are frequently feasible from the lower‐lying T_1_ triplet state (Figure 1A).[^12^, ^24^, ^25^, ^26^, ^27^] Direct excitation to the triplet state is spin‐forbidden and not observed for NB and a triplet sensitizer is thus required. The triplet state energy of NB, reported to be on the order of 2.7 eV,^[28]^ is better accessible with light of the solar spectrum compared to the singlet‐excited state. As a further benefit, the sensitization approach not only minimizes energy losses in the isomerization process but also permits inherently higher quantum efficiencies, because the isomerization of unsubstituted NB via the triplet‐excited state is intrinsically favored ($\Phi_{NB\to QC}$ ~ 1). This is due to the nature of the geometrically relaxed triplet state (as a 1,3 diradical) resembling closely the QC structure.[^28^, ^29^] Yet, two problems arise with the sensitization‐driven conversion of NB: (i) Sensitizing the triplet state (2.7 eV, 459 nm), requires high‐energy sensitizers that, owing to energy loss, absorb only a small fraction of the sunlight reaching Earth. In this context, ketones,^[12]^ acridones,^[30]^ Cu(I)‐complexes,[^31^, ^32^] transition‐metal doped TiO_2_,^[33]^ Rh‐^[34]^ and Ir‐complexes[^35^, ^36^] sensitizers and covalently bond sensitizer‐norbonadiene structures[^37^, ^38^] have been explored. Fast degradation of these sensitizers and/or costly synthesis renders them impractical for repeated large‐scale applications. (ii) NB is a highly reactive species in the triplet state and quickly degrades, reacting with the sensitizer itself making it unsuitable for multiple conversion cycles.[^39^, ^40^] To address these issues, norbornadienes bearing functional groups were investigated. While the triplet energy of these derivatives was essentially reduced, the interconversion efficiency to QC is negatively affected such that quantum yields well below 20 % resulted, and even cycloreversion to the NB isomer was observed.[^41^, ^42^]

All these findings highlight the complexities encountered in the development of new photoswitches for energy storage. The process is far from straightforward due to the diverse effects of structural modifications on the relevant MOST criteria. A profound understanding of the underlying mechanisms is crucial to overcome these hurdles, potentially paving the way for significant advancements in the field.[^14^, ^46^] Recently, one of our groups introduced mono‐, bis‐ and tris‐norbornadienyl substituted arenes that can be readily synthesized by a Suzuki–Miyaura coupling reaction.[^43^, ^44^] Exceptional energy storage densities (up to 734 kJ/kg) are achieved nearing those of unsubstituted NB as a result of the low molecular weight.^[47]^ However, direct excitation by solar irradiation is inefficient because excitation is only accomplished by the marginal UV fraction (<350 nm) (Figure 1C). The combination of π‐extension lowering the excited‐state energies and the absence of donor as well as acceptor groups potentially avoids photoinduced electron transfer. Hence, we speculated that these promising multichromophoric photoswitches^[48]^ could serve as a basis for exploring the potential of triplet energy transfer‐driven MOST systems. In this work, four aryl‐norbornadiene derivatives (Figure 1B) were selected that not only are highly promising regarding MOST applications but also enable us to clarify key photophysical properties focusing on triplet‐excited states. Through deep kinetic and mechanistic analyses, substantiated by quantum mechanical calculations and visible light driven conversion cycles, we gained substantial insights into the sensitized MOST systems. As we will show, these systems can be driven using visible photons close to the maximum of the terrestrial solar spectrum (~440–550 nm) with quantum yields approaching unity. Combined with the high energy density and the long half‐life of the ArQC isomers (compare, Figure 1B), the MOST systems presented herein satisfy essentially all aspects that are crucial for MOST applications.

## Results and Discussion

### Selection of Photoswitch–Sensitizer Pairs and Initial Energy Transfer Studies

**Figure 2:**
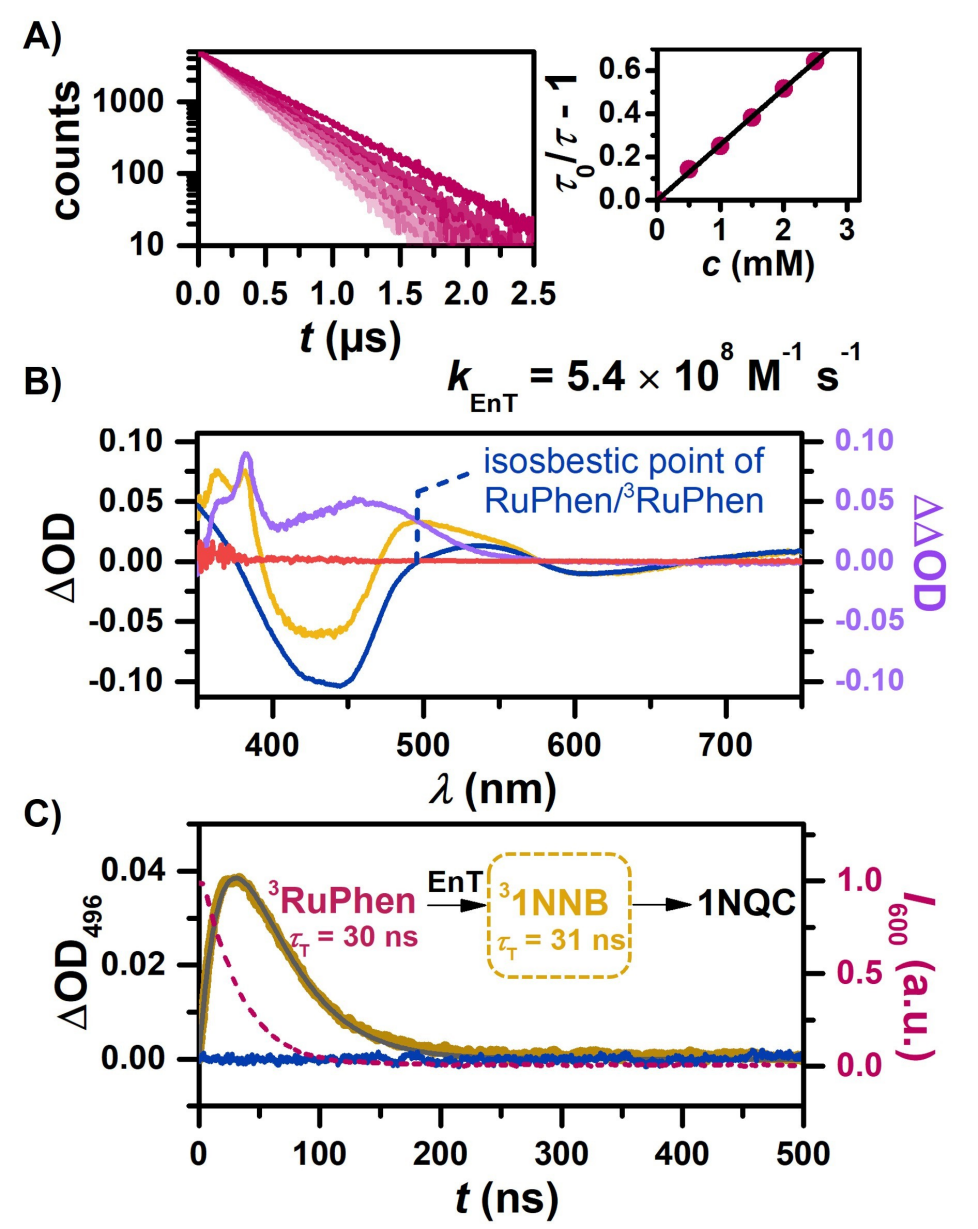
Mechanistic investigations of the triplet state of 1NNB by laser flash photolysis using 532 nm laser pulses. A) Kinetic emission of RuPhen with increasing 1NNB concentration and corresponding Stern–Volmer analysis. B) TA spectra of a solution of 100 μM RuPhen in the absence (dark blue) and presence of 55 mM 1NNB in deaerated MeCN recorded 30 ns (orange) and 1 μs (pink) after excitation (integration time, 20 ns). The reference spectrum of ^3^RuPhen was scaled to match in the region >600 nm in order to determine the respective difference spectrum (purple). C) Kinetic TA traces of both solutions at the isosbestic point of RuPhen (496 nm) and kinetic emission of RuPhen in the presence of 1NNB (dotted purple line). The triplet lifetime of 1NNB was determined by a kinetic analysis^[50]^ (gray line).

Among the aryl‐substituted norbornadienes (Figure 1B), Ph3NB is especially appealing due to its superior physical properties, the possibility of accessing multiple states, and increased energy density achieved by incorporating several energy storage units in one molecule.^[48]^ Nevertheless, spectroscopic investigations of multistate photoswitches are challenging, which is why PhNB, bearing a single NB moiety, serves as a reference compound in these studies. The isomer pair 1NNB/1NQC is notable for its 35‐day half‐life, which is highly advantageous for MOST applications.^[44]^ Furthermore, the distinctive absorption features of the naphthalene scaffold facilitate mechanistic investigations in ground‐state UV/Vis and transient absorption spectra. Although its structural isomer, 2NNB, may not possess equally appealing characteristics, examining it in conjunction with 1NNB may offer valuable understanding of how small structural changes affect the performance of photoswitches. Thus, our investigations employed both naphthalene derivatives that were initially combined with the well‐studied triplet sensitizer [Ru(phen)_3_](PF_6_)_2_ (RuPhen). The following experiments were conducted with 1NNB and 2NNB, for clarity only the results for 1NNB are displayed in the main manuscript (see Chapter S8.1). Initial Stern–Volmer experiments in MeCN revealed that excited RuPhen (unquenched lifetime, ~500 ns)^[49]^ is effectively quenched by the norbornadienes suggesting that triplet‐triplet energy transfer (EnT) is feasible (Figure 2A). However, studying triplet‐excited states of organic chromophores via sensitization, especially those with (ultra)short lifetimes like the 6.2 ns^[28]^ observed for unsubstituted norbornadiene, can be challenging because triplet formation and decay occur on similar time scales. Laser flash photolysis (LFP) with optical detection is a well‐suited method in this scenario, enabling the direct examination of such transient, non‐emissive triplet states that are otherwise difficult to probe.[^46^, ^50^, ^51^, ^52^] Pulsed laser excitation at 532 nm allowed us to selectively excite RuPhen and to study quenching products by transient absorption (TA) spectroscopy. High concentrations of 1NNB (55 mM) were employed to accumulate ^3^1NNB in large amounts facilitating precise and reliable measurements. After complete sensitizer quenching (pink spectrum in Figure 2B), only the baseline level of RuPhen was detected. This allows us to exclude long‐lived photoproducts typically generated by electron transfer or irreversible side reactions, which is also consistent with the anticipated short triplet lifetime. A TA spectrum recorded during the quenching process initially resembles the triplet‐triplet absorption spectrum of RuPhen (Figure 2B).^[53]^ Closer inspection, however, revealed decisive differences. A reference ^3^RuPhen spectrum recorded under similar conditions was appropriately scaled to match in the region >600 nm, where we do not expect ^3^1NNB to absorb. Subtracting this TA spectrum allows us to delineate what is presumed to be the triplet absorption spectrum of 1NNB, characterized by absorption bands at 460 nm, 382 nm and a shoulder at 365 nm. Comparing this absorption spectrum to unsubstituted naphthalene we find similar absorption bands around 400 nm (Figure S23). The additional broad absorption between 400 and 500 nm is also predicted by TD‐DFT calculations (Figure S2). The TA spectrum of ^3^RuPhen exhibits an isosbestic point at 496 nm, which lays the grounds for the isolated monitoring of the formation and deactivation of ^3^1NNB (Figure 2C). Fitting this measurement with conventional A (^3^RuPhen) → B (^3^1NNB) → C (photoproduct) kinetics a lifetime of 31 ns is obtained for ^3^1NNB.^[50]^ This is indeed much shorter than reported for the naphthalene triplet (~15 μs),^[54]^ indicating a much more efficient chemical deactivation pathway. Additional experiments with 2NNB yielded an even shorter lifetime (<15 ns). Interestingly, Takamuku et al. reported significantly longer triplet lifetimes for similar norbornadienyl‐naphthalene derivatives that included an extra carboxylate group.^[42]^ However, these derivatives performed poorly, attributed to low conversion yields, and even cycloreversion was observed. Microsecond lifetimes and degradation issues were recently observed for donor‐acceptor substituted norbornadienes in related sensitization laser experiments.^[46]^ Hence, it seems natural to assume that short triplet lifetimes, as observed for two aryl‐substituted norbornadienes (1NNB and 2NNB), are beneficial for sensitized MOST applications.

### Characterizing the Triplet‐Sensitized Conversion to Quadricyclane

**Figure 3:**
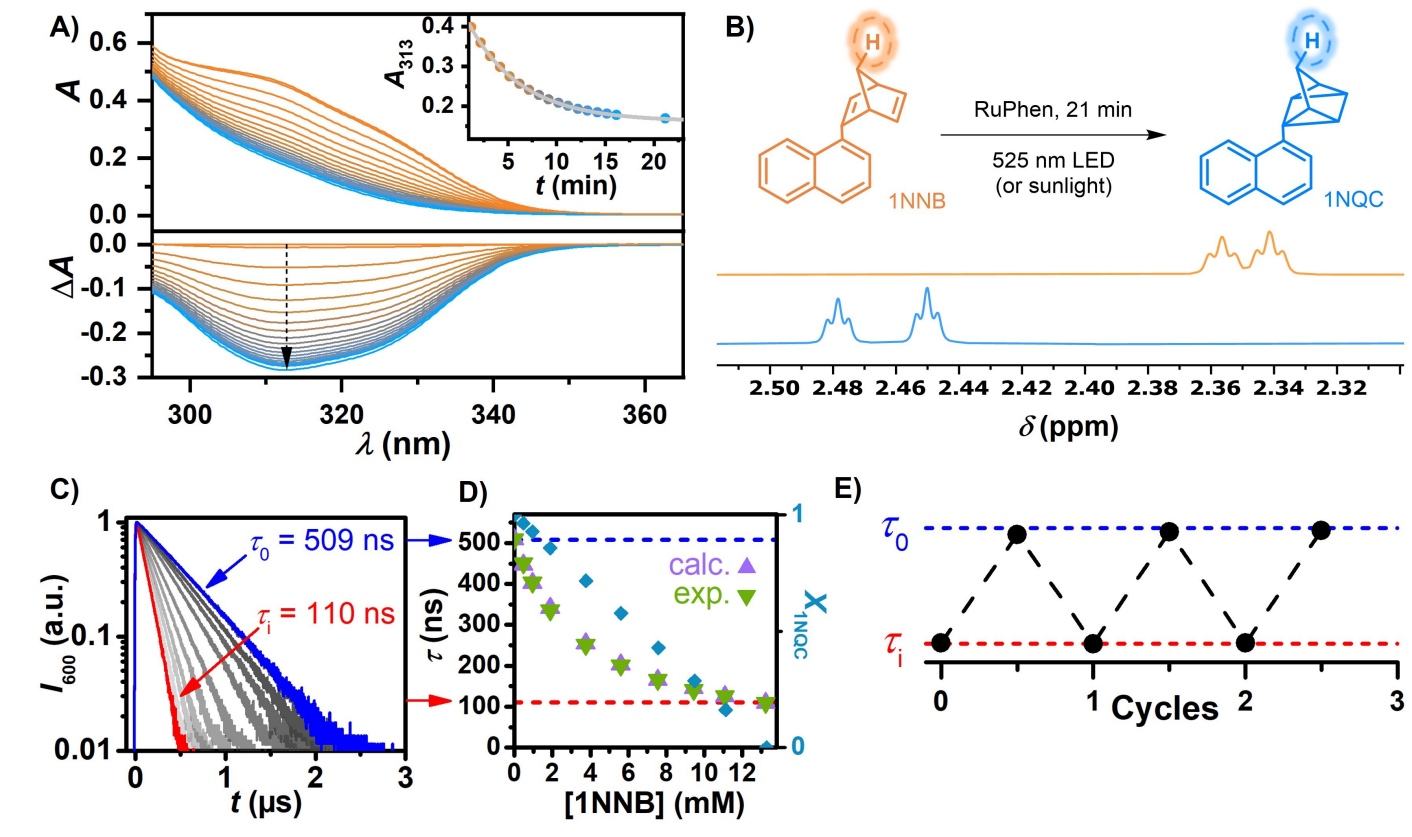
Quantification of the photoswitch state using NMR, UV/Vis absorption and room‐temperature phosphorescence lifetime measurements. A) Steady‐state UV/Vis measurements of 0.1 mM 1NNB and 100 μM RuPhen in Ar‐saturated MeCN, irradiated with a 525 nm LED. The absorption spectra are corrected by the absorption of RuPhen, which was used as baseline. Inset: Absorption at 313 nm plotted against irradiation time, fitted using an exponential decay function. B) NMR spectra displaying characteristic signals of 1NNB and 1NQC measured before and after 525 nm LED irradiation of a solution with 170 μM RuPhen and 30 mM 1NNB in Ar‐saturated CD_3_CN. C) Kinetic emission of 250 μM RuPhen and a total quencher concentration of 13.2 mM with varying ratios of 1NNB and 1NQC in Ar‐saturated MeCN upon 532 nm laser excitation. D) Experimental and calculated RuPhen lifetime plotted against the residual 1NNB concentration, and corresponding fraction of 1NQC. E) RuPhen lifetime monitored at 600 nm after laser excitation of 22.5 mM 1NNB and 250 μM RuPhen in Ar‐saturated MeCN during repeated irradiation (7 min, 525 nm LED) and heating (21 h, 65 °C) cycles.

To confirm the expected energy transfer‐induced switching, we investigated the lab‐scale transformation of 1NNB to 1NQC. A solution of 1NNB with RuPhen in Ar‐saturated MeCN was monitored by steady‐state absorption measurements during 525 nm LED irradiation (Figure 3A). The system reached a photostationary state within 20 minutes under our conditions. Larger‐scale NMR irradiation experiments with a RuPhen loading as low as 0.6 % indeed revealed that full conversion is achieved (Figure 3B). Even under solar irradiation 1NNB rapidly converts to 1NQC (Figure S41). In contrast, direct excitation in the absence of a sensitizer leads to inefficient and incomplete conversion (25 % 1NQC) as the absorption of the corresponding photoisomer is usually not negligible causing undesired photoreversion (Figure S41).[^6^, ^43^] Upon direct 315 nm excitation of a strongly diluted 1NNB solution (20 μM) up to 80 % of the desired 1NQC could be obtained in the photostationary state.^[55]^ Hence, the sensitization approach not only improved the overlap with the solar spectrum by about 200 nm but also allows rapid and complete conversion of larger amounts of 1NNB.

At different conversion stages of 1NNB to 1NQC kinetic laser measurements indicate a progressive increase of the sensitizer's emission lifetime (Figure 3C). At full conversion (100 % 1NQC) RuPhen maintains its inherent unquenched triplet lifetime, which is unaffected by 1NQC despite relatively high concentrations. This seems reasonable since the conversion of the double bonds to the quadricyclane unit renders the compound structurally similar to alkyl‐substituted naphthalene effectively changing the triplet energy. Complementary DFT calculations of the lowest excited triplet states of 1NNB and 1NQC reveal significant spin density on the double bonds of the NB moiety while contributions of the quadricyclane unit are negligible (SI, Chapter S3). Assuming that the triplet energy is similar to that of naphthalene (~2.6 eV) the energy transfer rate constant with the sensitizer RuPhen (2.19 eV) is expected to be negligibly low,^[56]^ which is in line with our observations. Rearrangement of the Stern–Volmer equation (Chapter S1) led us to derive a method to monitor the progress of the conversion of 1NNB to 1NQC simply based on the readily accessible RuPhen emission. Lifetime measurements of RuPhen were carried out with varying ratios of 1NNB and 1NQC (Figure 3D). We observed that the measured lifetime perfectly correlates with the calculated lifetime (i.e., calculated based on the 1NNB concentration and the EnT rate constant). This confirms the validity of this strategy, which has clear advantages over NMR‐ and UV/Vis‐based techniques as it is fast and it does not rely on strongly diluted solutions. This simple method can be easily adopted in most laboratories to efficiently obtain information of the photoisomerization progress and does not require sophisticated laboratory setups.^[40]^ Conversion cycles of 1NNB were monitored by this simplistic approach (Figure 3E).

### Switching Quantum Yield and Triplet Energy Determinations

The efficiency of the light‐driven part of MOST systems is fundamentally determined by the overall quantum yield Φ expressed as follows in our sensitized system:

(1) $$\Phi =\;\Phi_{ISC}\times \Phi_{EnT}\times \;\Phi_{NB\to QC\;}$$

**Figure 4:**
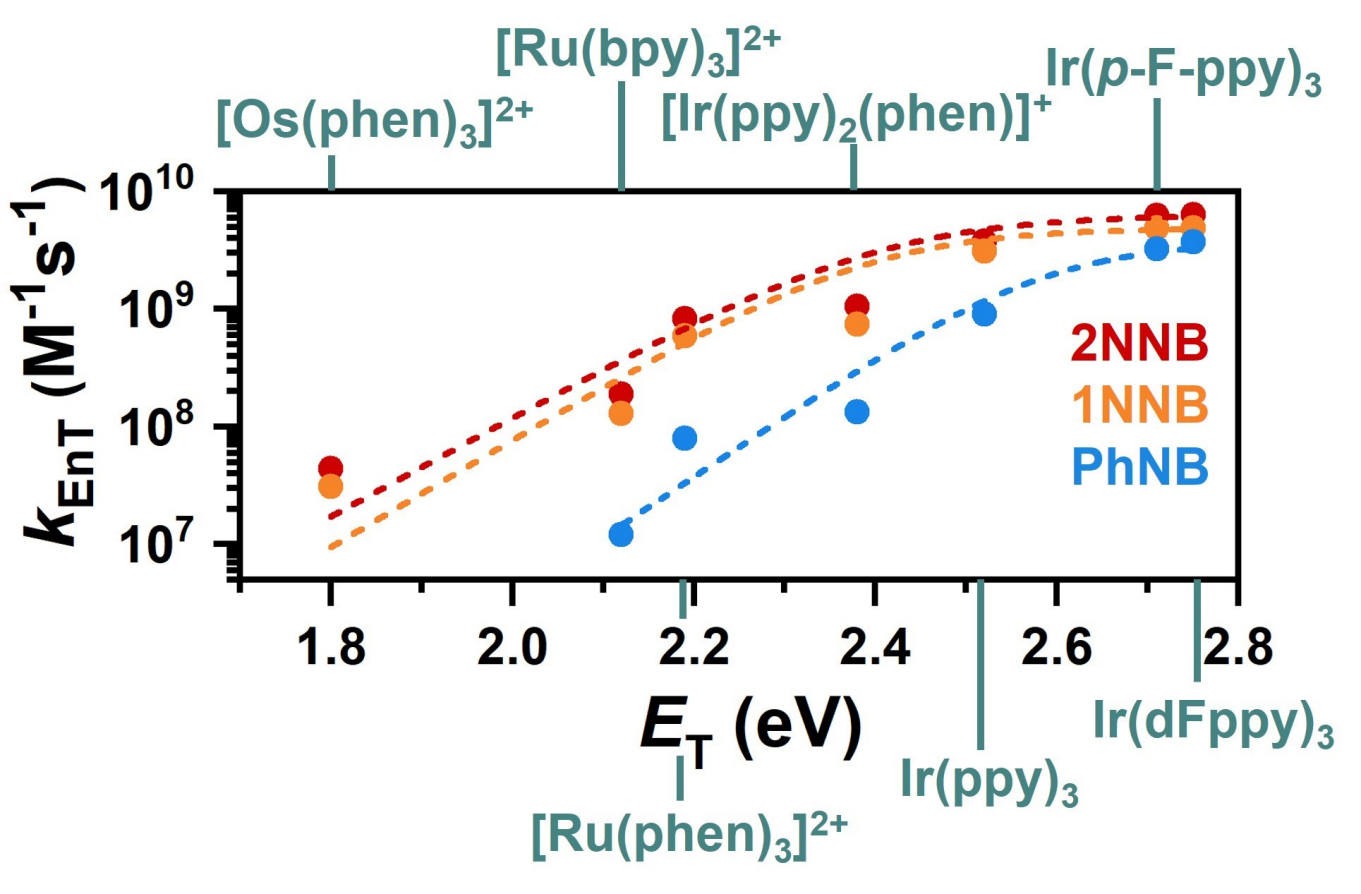
Rate constants of triplet energy transfer between excited metal sensitizers and norbornadienes plotted against the triplet energies of the energy donors with a thermal bond activation model fitted to the experimental data. The energy transfer rates in MeCN were determined by Stern–Volmer analyses. A fit function related to the Sandros equation was selected. For details see SI, Chapter S4.1.

Under operational conditions, the intersystem crossing quantum yield ($\Phi_{ISC}$ ) of RuPhen^[57]^ and the triplet energy transfer quantum yield ($\Phi_{EnT}$ ) approach unity, as demonstrated by kinetic simulations (SI; Figure S14). The quantum yield for the intrinsic transformation of triplet‐excited NB to QC ($\Phi_{NB\to QC}$ ) is a pivotal metric. This quantity was obtained for 1NNB through relative chemical actinometry by using Ir(ppy)_3_ as the sensitizer for both switching 1NNB and catalyzing a well‐established reference reaction (SI, Chapter S5).^[58]^ The findings indicated that the isomerization quantum yield for 1NNB is very close to unity within the experimental error (1.01±0.06), paving the way for solar energy conversion systems with ideal efficiencies. Having established the highly efficient sensitized conversion of an aryl‐norbornadiene to the corresponding quadricyclane, we conducted a more detailed series of experiments. These experiments focus on the triplet state energies of our chromophores to identify the most suitable sensitizer.[^50^, ^59^] In this context, a series of metal complex sensitizers with known triplet energies was employed in quenching studies with the energy acceptors 1NNB, 2NNB and PhNB (Figure 4).

Essentially diffusion‐controlled energy transfer rate constants were observed with the high‐energy sensitizers (triplet energy >2.7 eV). These rate constants decrease as expected with the sensitizer‘s triplet energy. Although the energy transfer rate decreases by two orders of magnitude when [Os(phen)_3_]^2+^ is used, high concentrations of 1NNB and 2NNB, which would be required for larger‐scale applications, could ensure substantial energy transfer efficiencies (Figure S14). Given that [Os(phen)_3_]^2+^ absorbs light across the entire visible spectrum, significant solar harvesting efficiencies can in principle be realized with this sensitizer–photoswitch couple. Based on the results displayed in Figure 4, we estimate a triplet energy of 2.39 eV for 1NNB, 2.41 eV for 2NNB and 2.56 eV for PhNB (see Chapter S4.1). Given that such deep spectroscopic insights are tricky to obtain with all intermediates of the multistate switch Ph3NB, DFT calculations were carried out to estimate the adiabatic triplet energies of all isomers as well as the benchmark core structures benzene (PhH) and naphthalene (NH) (see Table 1 and Supporting Information Chapter S3). While the calculations likely underestimate the triplet energies due to the large structural changes after vertical excitation, a pattern is observed. The naphthyl‐quadricyclanes possess triplet state energies ~0.5 eV higher compared with their corresponding norbornadienes, aligning them closely with the benchmark structure NH. Spin density analyses underscore significant contributions from the double bonds in the norbornadiene moieties, whereas the corresponding quadricyclanes closely resemble naphthalene triplets, as detailed in Chapter S3. This notable triplet energy difference between the photoisomers corroborates the observed selective quenching of ^3^RuPhen with 1NNB. Additional mechanistic investigations were conducted using the high triplet energy sensitizer thioxanthone TX (2.75 eV), in combination with 1NNB. We observed that the conversion of 1NNB to 1NQC is incomplete and the triplet lifetime of TX is still substantially quenched. These observations let us conclude that reactivation of 1NQC is in principle feasible (Chapter S8.3), further underscoring the importance of mechanistic studies and triplet energy determinations.

**Table 1:** Triplet energies computed by DFT of arenes, aryl‐substituted norbornadienes and corresponding quadricyclane isomers (B3LYP/6‐311+G(d,p) level of theory).

Compound	*E*_T_^[a]^/eV
PhH	3.83^[b]^
PhNB	2.11
PhQC	0.90^[c]^
Ph3NB	2.08
Ph2NB1QC	2.10
Ph1NB2QC	2.10
Ph3QC	4.20
NH	2.69^[d]^
1NNB	1.97
1NQC	2.51
2NNB	1.99
2NQC	2.64

[a] Adiabatic triplet state energies. [b] Experimental triplet energy of benzene, 3.9 eV.^[60]^ [c] Bond cleavage during optimization (one bond within the QC moiety). [d] Experimental triplet energy of naphthalene, 2.63 eV.^[54]^

### Mechanistic Studies with the Multistate Photoswitch

**Figure 5:**
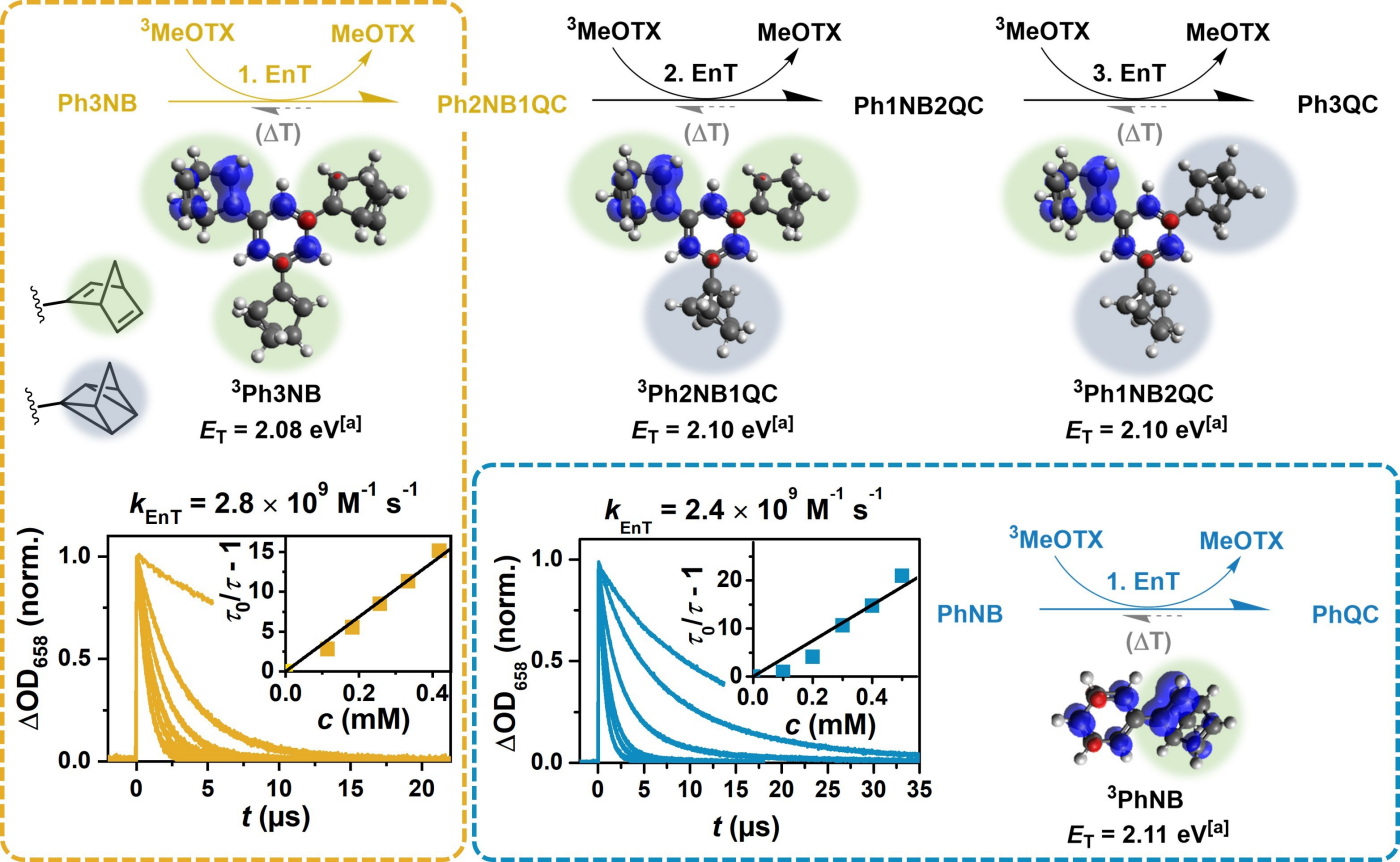
Reaction scheme of the cyclization of Ph3NB (top) and PhNB (bottom right) to the corresponding quadricyclane isomer using MeOTX as sensitizer (see Figure 6 for its structure). Comparative Stern–Volmer quenching experiments for the respective (first) energy transfer step are shown. The spin densities of the triplet‐excited starting compounds (Ph3NB and PhNB) and the intermediates Ph2NB1QC and Ph1NB2QC are displayed below each energy transfer step (B3LYP/6‐311+G(d,p) level of theory). [a] Computed by DFT (see Table 1).

The calculated triplet energies of the benzene derivatives Ph3NB, Ph2NB1QC, Ph1NB2QC and PhNB are virtually identical. In accordance with this result, we find that the spin density in the triplet excited state of each NB‐containing isomer of the multistate switch is mostly located on only one NB moiety (Figure 5), which is expected for meta‐substituted benzene derivatives.

**Figure 6:**
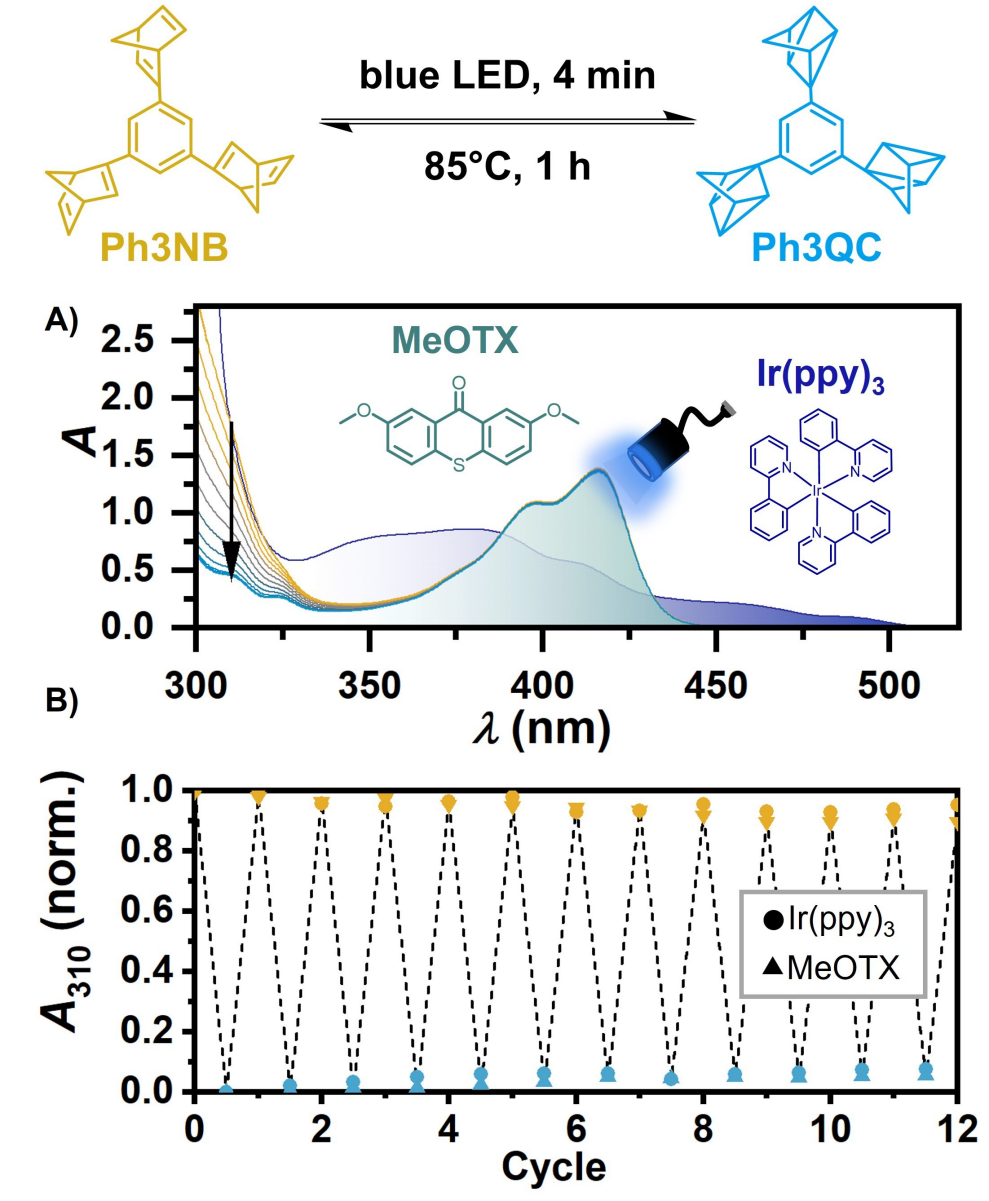
A) Photometric monitoring of the isomerization of Ph3NB to Ph3QC. A solution of 400 μM Ph3NB with 245 μM MeOTX as sensitizer in deaerated toluene was used. A 440 nm LED served as the light source. Absorption spectra were recorded with intervals of 20 s. Complementary experiments with Ir(ppy)_3_ can be found in the Supporting Information (Figure S29). For clarity only the starting absorption spectrum of 300 μM Ph3NB with 50 μM Ir(ppy)_3_ is displayed. B) Normalized absorption at 310 nm of the reversible conversion between Ph3NB and Ph3QC by sequential irradiation (440 nm, 4 min) and thermal treatment (85 °C, 1 h) for a total of 12 cycles using Ir(ppy)_3_ or MeOTX as photosensitizer.

Analyzing the conversion of Ph3NB to Ph3QC by spectroscopic methods is somewhat cumbersome involving three separate isomerization reactions. While the first photoisomerization step can be monitored spectroscopically when stopping the Ph3NB sensitization at very low conversion, the second and third steps require tedious isolation of the isomers Ph2NB1QC and Ph1NB2QC. The last conversion of Ph1NB2QC to Ph3QC, however, is key for optimal performance and typically the hardest to achieve by direct excitation.^[43]^ The calculated triplet energy of PhNB is very similar to that of Ph1NB2QC and this allows us to assume that when disregarding steric effects the photophysical properties of both compounds are comparable. These detailed investigations enabled us to identify 2,7‐dimethoxy‐thioxanthone (MeOTX) as an appropriate sensitizer for the conversion of the multistate Ph3NB, along with monosubstituted PhNB as reference compound. The UV/Vis absorption spectrum of MeOTX is red‐shifted by about 40 nm compared to TX such that MeOTX readily absorbs blue light (*ϵ*_440nm_=580 M^−1^ cm^−1^) and exhibits a triplet state energy of 2.38 eV^[50]^ with a lifetime of ~15 μs in toluene (Figure 6).^[61]^

Although triplet energy transfer from MeOTX to Ph3NB and PhNB (experimental triplet energy, 2.56 eV) is seemingly thermodynamically uphill, efficient isomerization remains feasible for three reasons: (i) Even at EnT rate constants significantly below the diffusion limit, the energy transfer efficiency can approach high values due to the much longer triplet lifetime of MeOTX compared to the employed metal sensitizers (Figure S14). (ii) The short triplet lifetime of the norbornadienes ensures conversion to quadricyclane before undesired back energy transfer to the sensitizer can occur, which usually limits uphill energy transfer. (iii) Thioxanthone‐derived sensitizers are reported to have lower reorganization energies compared to Ir‐based sensitizers. This ultimately results in faster sensitization even when the triplet energies of the sensitizers are comparable.^[62]^ LFP quenching studies with ^3^MeOTX revealed efficient and comparable energy transfer rate constants to the chromophores PhNB and Ph3NB (Figure 5). These experiments substantiate the conclusions drawn from quantum mechanical calculations. NMR‐scale experiments confirm that MeOTX quantitatively converts Ph3NB to Ph3QC under 440 nm LED irradiation (Figure S39). Based on our findings on all norbornadiene derivatives analyzed in this study, we conclude that the conversion of each norbornadiene unit (which cannot be distinguished by ^1^H NMR spectroscopy; see SI, Chapter S6, for details) can be considered as equally efficient and light‐driven back reactions are regarded to be unimportant owing to the high triplet energy of Ph3QC. A remarkable inherent quantum efficiency for the conversion of Ph3NB ($\Phi_{NB\to QC}$ ) approaching 100 % was determined by relative actinometry (SI, Chapter S6). Given that, under operational conditions (compare equation 1), nearly every absorbed photon converts one norbornadiene unit, the maximum efficiency has already been reached. To the best of our knowledge, this highly efficient visible light‐driven MOST system with its high energy storage capabilities and its long thermal stability of the photoisomer (compare Figure 1) is unprecedented, combining beneficial properties of four essential MOST criteria as discussed in the introduction.

### Photostability and Multiple Solar‐Driven Conversion Cycles

**Figure 7:**
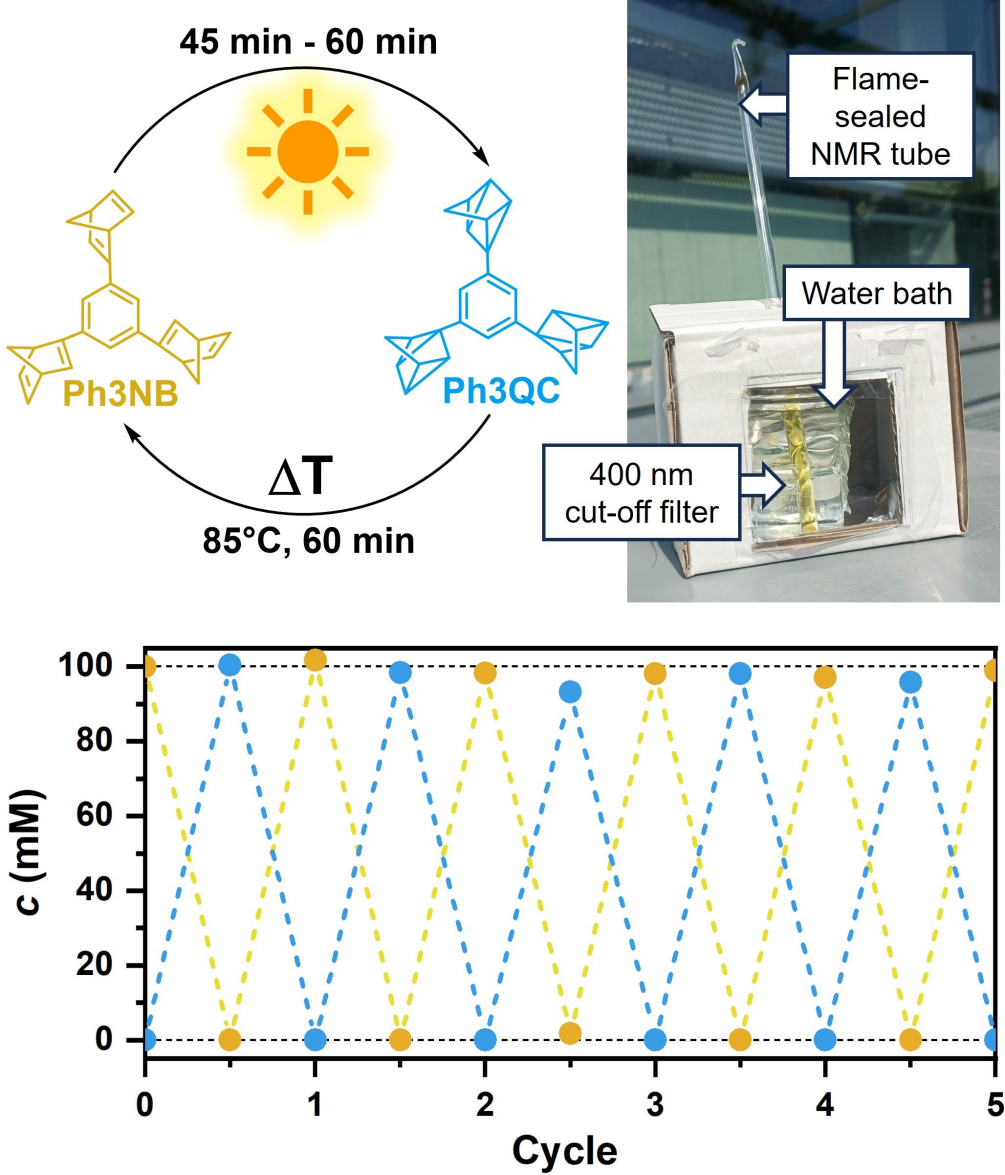
Experimental setup of the solar‐driven conversion cycles of a solution of 100 mM Ph3NB with 590 μM Ir(ppy)_3_ (0.59 mol %) and 26.9 mM bibenzyl (standard) in Ar‐saturated deuterated toluene. The concentrations of Ph3NB and Ph3QC are given for each cycle.

The photostability is another factor determining the practicability of a MOST system. To estimate the stability, several irradiation/thermal treatment cycles using a 440 nm LED were performed with a solution containing MeOTX and Ph3NB, and monitored by steady‐state absorption (Figure 6A). Over 12 conversion cycles the degradation is marginal (Figure 6B) and mainly a result of unquenched sensitizer decomposition typical for organic chromophores (Figure S31). Using Ir(ppy)_3_ as sensitizer not only enables the harvesting of photons up to 523 nm but also reduced the decomposition to ~1 % per cycle, which compares favorably with the stability of a strongly related multiswitch aryl‐norbornadiene investigated upon 315 nm excitation.^[43]^ To demonstrate the applicability of the Ir(ppy)_3_–Ph3NB MOST system at high norbornadiene concentrations, we tested a solution with 100 mM Ph3NB and 0.59 mol % Ir(ppy)_3_. This solution underwent five cycles of irradiation and thermal treatment using visible sunlight, deliberately avoiding direct UV excitation of Ph3NB (Figure 7). Even at elevated concentrations, the degradation remained minimal. We attribute the comparatively high photostability of the sensitized MOST system to the absence of electron transfer processes that are typically observed when highly functionalized compounds are used as quenchers.[^63^, ^64^]

For the promising Ir(ppy)_3_–Ph3NB MOST system with the specified concentrations, we calculated a maximum energy storage efficiency of 5.8 %. This compares very favorably to the efficiency of 0.53 % for direct solar excitation of Ph3NB (SI, Chapter S7) and to that of most systems so far analyzed with this efficiency parameter.[^3^, ^65^, ^66^, ^67^, ^68^]

## Conclusion

As has emerged from this work, the combination of photostable sensitizers with aryl‐norbornadiene switches as energy acceptors is a highly promising field for obtaining MOST systems with outstanding properties. Through the combination of quenching studies, detailed LFP experiments, quantitative irradiation experiments and quantum mechanical calculations we could unambiguously show that (i) aryl‐substituted norbornadienes inherently have low triplet energies paving the way for 200 nm red‐shifts of the photoaction spectrum compared to direct UV excitation, (ii) the resulting triplets rapidly deactivate to give the desired quadricyclanes with quantum yields very close to 100 % and (iii) quantitative unidirectional switching via sensitization is feasible. The wealth of information from this study adds to inherently unique properties of recently discovered aryl‐norbornadienes such as superior energy densities of the photoisomers and sufficiently long conservation times. Based on our promising results, our study might stimulate the broader application of sensitization strategies in MOST systems. In summary, our results not only deepen the understanding of triplet‐sensitized photoisomerization processes and the critical parameters influencing MOST applications but also introduce a visible light‐driven MOST system that combines outstanding energy conversion and storage efficiencies with promising robustness for the first time.

## Supporting Information

The Supporting Information contains synthetic and experimental procedures, spectroscopic data, detailed mechanistic investigations including quantum yield determinations, kinetic simulations and Stern–Volmer plots, as well as quantum chemical calculations. The authors have cited additional references within the Supporting Information.[^69^, ^70^, ^71^, ^72^, ^73^, ^74^, ^75^, ^76^, ^77^, ^78^, ^79^, ^80^]

## Conflict of Interests

The authors declare no conflict of interest.

1

## Supporting information

As a service to our authors and readers, this journal provides supporting information supplied by the authors. Such materials are peer reviewed and may be re‐organized for online delivery, but are not copy‐edited or typeset. Technical support issues arising from supporting information (other than missing files) should be addressed to the authors.

Supporting Information

## Data Availability

The data that support the findings of this study are available in the main article and/or the supplementary material. The data sets shown in the main paper and DFT output files are accessible via the JGU library and the homepage of the corresponding author.
