# Supplementary material for: Triplet‐Sensitized Switching of High‐Energy‐Density Norbornadienes for Molecular Solar Thermal Energy Storage with Visible Light
Source: Angew Chem Int Ed Engl. 2024 Nov 2;64(2):e202414733. doi: 10.1002/anie.202414733 (PMC11720394; doi:10.1002/anie.202414733)
Supplement: Supplementary file 1 — Supporting Information [file ANIE-64-e202414733-s001.pdf]

Angewandte Chemie
Eine Zeitschrift der Gesellschaft Deutscher Chemiker

Supporting Information

Triplet-Sensitized Switching of High-Energy-Density Norbornadienes for Molecular Solar Thermal Energy Storage with Visible Light

T. J. B. Zähringer, N. Perez Lopez, R. Schulte, M. Schmitz, H. Ihmels, C. Kerzig**

Supporting Information for

Triplet-Sensitized Switching of High-Energy-Density Norbornadienes for Molecular Solar Thermal Energy Storage with Visible Light

Till J. B. Zähringer*[a], Nico Perez Lopez[a], Robin Schulte[b], Matthias Schmitz[a], Heiko Ihmels[b] and Christoph Kerzig*[a]

[a] T. J. B. Zähringer, N. Perez Lopez, M. Schmitz, Prof. Dr. C. Kerzig
Department of Chemistry
Johannes Gutenberg University Mainz
Duesbergweg 10-14, 55128 Mainz, Germany
E-mail: tizaehri@uni-mainz.de, ckerzig@uni-mainz.de

[b] R. Schulte, Prof. Dr. H. Ihmels
Department of Chemistry-Biology, and Center of Micro- and Nanochemistry and (Bio-)Technology (Cμ)
University of Siegen
Adolf-Reichwein-Str. 2, 57068 Siegen, Germany

Table of contents

1. General information.....2
2. Synthetic procedures.....4
3. DFT calculations.....6
4. Stern–Volmer experiments.....11
4.1. Triplet-triplet energy transfer studies of PhNB, 1NNB and 2NNB with various metal sensitizers.....11
4.2. Additional triplet energy transfer studies of various sensitizers and triplet acceptors.....15
4.3. Kinetic simulations of the energy transfer efficiency.....16
5. Interconversion quantum efficiency of 1NNB to 1NQC determined by relative actinometry.....16
6. Interconversion quantum efficiency of Ph3NB to Ph2NB1QC determined by relative actinometry.....20
7. Maximum energy storage efficiency of the Ir(ppy)3–Ph3NB sensitizer–photoswitch pair.....23
8. Additional spectroscopic measurements.....24
8.1. Mechanistic investigations of the triplet state of 2NNB.....25
8.2. Steady-state absorption spectra.....27
8.3. Photostationary state composition of triplet sensitized 1NNB/1NQC.....28
9. Multiple conversion cycles of the Ir(ppy)3–Ph3NB pair under solar irradiance.....32
10. NMR spectra.....33
11. Literature.....43

1. General information

Materials

Unless specifically mentioned, all chemicals used for synthesis, irradiation experiments, or optical spectroscopy were commercially obtained and used as received (acetonitrile, 99.9%, Fisher Scientific; toluene, 99.7%, Thermo Scientific; toluene-d_8, 99.5%, deutero; acetonitrile-d_3, 99.0%, deutero; *fac*-[Ir(ppy)$_3$], 99%, Sigma-Aldrich; thioxanthone, 97%, Sigma-Aldrich (purified by recrystallization from toluene/EtOAc prior to use); 2,7-dimethoxy-9H-thioxanthen-9-one (MeOTX), 98%, BLD Pharmatech; tris[5-fluoro-2-(2-pyridinyl)phenyl]-Iridium (IrF), 99%, Sigma-Aldrich; tris[3,5-difluoro-2-(2-pyridinyl)phenyl]-Iridium (IrdF), 98%, Sigma-Aldrich; (*E*)-ethyl cinnamate, 98%, Thermo scientific; [Ru(bpy)$_3$]Cl$_2$, 99.95%, Sigma-Aldrich; [Ru(phen)$_3$]Cl$_2$, 98%, Sigma-Aldrich; potassium hexachloroosmate, >99%, Sigma-Aldrich; 1,10-phenanthroline monohydrate, >98%, TCI; ammonium hexafluorophospate (NH_4PF_6), 98%, abcr; ethylene glycol, ≥99%, Carl Roth; naphthalene, 99.6%, Alfa Aesar; norbornadiene (stabilized with 250 ppm dibutylhydroxytoluene, BHT), 97%, TCI; NaOH, 97%, VWR; bromobenzene, 99%, Sigma-Aldrich; Pd(PPh$_3$)$_4$, 98%, Carbolution; THF, 99.5%, Thermo Fisher; Et$_2$O, >95%, VWR; *n*-hexane, >95% (distilled before used), VWR; 1,3,5-tribromobenzene, 97%, Carbolution; 1-bromonaphthalene, 97%, Alfa Aesar; 2-bromonaphthalene, 97%, Acros Organics; dimethyl sulfone, 98%, Thermo Scientific; dioxan, >99.5%, VWR; bibenzyl, 99%, Sigma-Aldrich.

Argon from Nippon Gases (5.0) was used for removing dissolved oxygen before all experiments related to optical spectroscopy.

Steady-state measurements

Absorption spectra were recorded with a Perkin Elmer LAMBDA 365 UV-Vis spectrophotometer. Emission measurements were carried out with a Perkin Elmer FL-6500 spectrometer and the emission spectra were corrected for the wavelength-dependent sensitivity of the instrument. All spectra were recorded at room temperature (295 ± 3 K) using 10 mm quartz glass cuvettes.

LED and solar irradiation experiments

Before all irradiation experiments, dissolved oxygen was removed by three freeze-pump-thaw cycles and sealed in custom-made Schlenk-cuvettes or NMR tubes.[1]

A 440 nm or 525 nm LED (Kessil Science, PR160L) with an average intensity reported as 352 mW cm^{-2} (1 cm distance) and a 405 nm LED from Thorlabs (M405L4) with an intensity of 14.53 µW/mm^2 at 100% (2 cm distance) as stated by the manufacturer were employed for irradiation experiments. The emission spectra are shown in Figure S1.

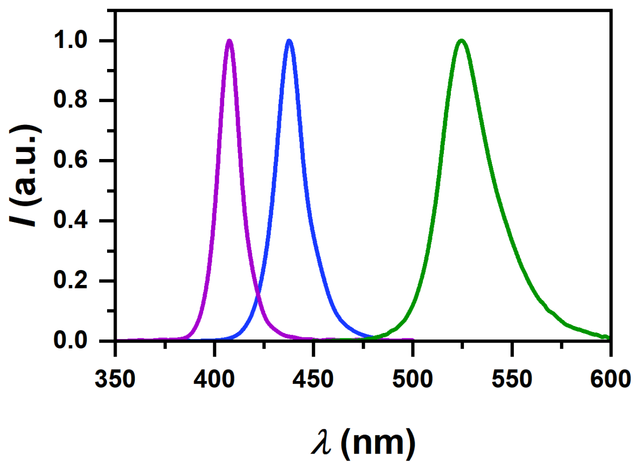

Figure S1: Normalized emission spectra of the 405 nm (purple), 440 nm (blue) and 525 nm (green) LEDs. The spectra were measured by irradiation a water-containing cuvette in the FL-6500 instrument, recording the Rayleigh scattered light. The light source was installed in a 90° angle with respect to the detector.

All solar irradiation experiments were performed in Mainz, Germany (49°59'N, 8°13'E) with no cloud coverage; date and irradiation times are given below the measurements.

Laser flash photolysis (LFP)

The LP980KS setup from Edinburgh Instruments equipped with an Nd:YAG laser from Litron (Nano LG 300-10) and an Nd:YAG Quantel (Q-smart 450) were employed for transient absorption and time-resolved emission spectroscopy. The frequency-doubled (532 nm) and frequency-tripled (355 nm) output served as the excitation source. The laser pulse duration was ~5 ns and the pulse frequency was 10 Hz. The typical pulse energy used for transient absorption and emission studies was ~20 mJ. A constant laser pulse energy was used within a series of experiments, which was ensured by power measurements before, after and in-between the experiments. The laser power was measured using a pyroelectric detector with attenuator (QE25LP-S-MB-QED-D0) from Gentec-eo combined with the integra software. Detection of transient absorption spectra occurred on an iCCD camera from Andor. Kinetic traces at selected wavelengths were recorded using a photomultiplier tube. The spectroscopic experiments were performed at 293 K using a cuvette holder that allows temperature control. If not stated otherwise the TA spectra were integrated over 100 ns. Longpass filters (385 nm or 400 nm, both from Newport) were used for transient absorption measurements with detection wavelengths exceeding 600 nm to exclude contributions from second-order signals.

Time-correlated single photon counting (TCSPC)

The *mini*-τ setup from Edinburgh Instruments (time-correlated single photon counting (TCSPC) technique) equipped with a pulsed laser (EPL-450, excitation at 446.4 nm, pulse width 74.5 ps) was employed for phosphorescence lifetime measurements of metal-based triplet sensitizers at 293 K. Stray light was removed with band pass filters and neutral density filters were used to avoid oversaturation of the detector.

Triplet-triplet energy transfer rate constants

Triplet-triplet energy transfer rate constants were determined by Stern–Volmer analyses. For Ir-based sensitizers this was achieved by lifetime-based measurements (τ) with varying concentrations c_q of the quencher and fitted according to the Stern–Volmer equation shown below (equation 1).

$$\frac{\tau_0}{\tau_i} = k_{\mathrm{EnT}} \cdot c_{\mathrm{i}} \cdot \tau_0 + 1 \tag{1}$$

Considering that the converted quadricyclane 1NQC does not deactivate the triplet sensitizer, we derived the following equation that allows us to monitor the conversion progress simply by measuring the sensitizer's lifetime. Rearranging the Stern–Volmer equation for c_{i} (sum of $c_{1\mathrm{NQC}}$ and $c_{1\mathrm{NNB}}$) gives us equation (2). Since the sensitizer lifetime is only dependent on the residual 1NNB concentration we derive equation (3) with $c_{1\mathrm{NNB}}$ as the concentration of 1NNB and τ_{j} as the measured sensitizer lifetime.

$$c_{\mathrm{i}} = \frac{\left(\frac{\tau_0}{\tau_{\mathrm{i}}} - 1\right)}{k_{\mathrm{EnT}} \cdot \tau_0} \tag{2}$$

$$c_{1\mathrm{NNB}} = \frac{\left(\frac{\tau_0}{\tau_{\mathrm{j}}} - 1\right)}{k_{\mathrm{EnT}} \cdot \tau_0} \tag{3}$$

The fraction of 1NQC ($X_{1\mathrm{NQC}}$) is given by equation (4) with c_{NQC} as the concentration of 1NQC. Inserting equation (2) and (3) into (4) gives us the dependence of the fraction of 1NQC and the sensitizer's lifetime, while τ_{i} and c_{i} are constant and correspond to the sensitizer lifetime and quencher concentration when 100% 1NNB is present. On the other hand, τ_{j} is the experimental lifetime that is obtained from lifetime-based measurements. Once τ_{j} is equal to τ_{i} full conversion is obtained.

$$X_{1\mathrm{NQC}} = \frac{c_{1\mathrm{NQC}}}{c_{\mathrm{i}}} = \frac{c_{\mathrm{i}} - c_{1\mathrm{NNB}}}{c_{\mathrm{i}}} \tag{4}$$

$$X_{1\mathrm{NQC}} = 1 - \frac{\left(\frac{\tau_0}{\tau_{\mathrm{j}}} - 1\right)}{\left(\frac{\tau_0}{\tau_{\mathrm{i}}} - 1\right)} \tag{5}$$

NMR spectroscopy

NMR spectra were recorded using either a multinuclear magnetic resonance spectrometer of the type AV II 400 (Bruker, Karlsruhe, Germany, ^{1}H: 400 MHz) or with a JEOL ECZ 500 spectrometer (^{1}H: 500 MHz,^{13}C: 125 MHz, 25 °C). The chemical shifts *δ* are stated in ppm and referenced to the residual solvent signal. The resonance multiplicity is abbreviated as: s (singlet), d (doublet), t (triplet), q (quadruplet) and m (multiplet).

2. Synthetic procedures

[Os(phen)$_3$](PF$_6$)$_2$, [Ru(phen)$_3$](PF$_6$)$_2$ and [Ru(bpy)$_3$](PF$_6$)$_2$

[Os(phen)$_3$](PF$_6$)$_2$ was prepared by a modified procedure of Constable *et al.*[2] Briefly, 1,10-phenanthroline monohydrate (0.20 g, 1.00 mmol, 3.0 equiv.), potassium hexachloroosmate (0.16 g, 0.33 mmol, 1.0 equiv.), and ethylene glycol (100 mL) were combined in a three-necked 250 mL round-bottom flask fitted with a Dimroth condenser. The solution was purged with argon for 30 minutes to remove dissolved oxygen and then refluxed for 2 hours. After cooling to room temperature, the reaction mixture was poured into 100 mL of an aqueous solution containing NH_4PF_6 (1.00 g, 6.10 mmol, 18.0 equiv.) and stirred for 15 minutes. A black precipitate formed, which was isolated by filtration, washed several times with MilliQ water, and dried under vacuum to afford a black solid (0.29 g, 0.28 mmol, 85% yield).

[Ru(phen)$_3$](PF$_6$)$_2$ and [Ru(bpy)$_3$](PF$_6$)$_2$ were synthesized according to a straightforward counterion exchange reaction with NH_4PF_6. The chloride salts ([Ru(phen)$_3$]Cl$_2$ and [Ru(bpy)$_3$]Cl$_2$) were dissolved in water and added to a saturated aqueous solution of NH_4PF_6. The precipitated [Ru(phen)$_3$](PF$_6$)$_2$, [Os(phen)$_3$](PF$_6$)$_2$, [Ru(bpy)$_3$](PF$_6$)$_2$ were washed thoroughly with cold water and acetonitrile.

[Ir(ppy)$_2$(phen)]PF$_6$ (IrPhen)

The sensitizer was synthesized according to a literature report by Bernhard *et al.*[3]

The ^{1}H NMR (DMSO-d$_6$) signals closely match the findings documented in the literature.[3]

Synthesis of the norbornadienes

All four aryl-substituted norbornadienes used in this study and the common precursor 4,4,5,5-tetramethyl-2-(bicyclo[2.2.1]-heptadien-2-yl)1,3,2-dioxaborolane are fully characterized prior to this study and were synthesized according to the procedure previously reported by us.[4–6] The exact amounts and yields for the aryl-substituted norbornadienes are given below.

1-(bicyclo[2.2.1]hepta-2,5-dien-2yl)benzene
(PhNB)

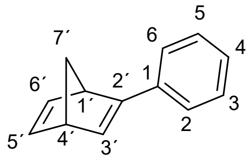

Under anaerobic conditions, an aqueous solution of NaOH (2.7 M, 5.8 mmol, 2.1 ml) was added to a solution of 4,4,5,5-tetramethyl-2-(bicyclo[2.2.1]-heptadien-2-yl)1,3,2-dioxaborolane (284 mg, 1.30 mmol), bromobenzene (187 mg, 1.19 mmol) and Pd(PPh$_3$)$_4$ (138 mg, 119 µmol) in THF (5.0 ml) and the resulting solution was stirred at 80 °C for 18 h. After cooling the reaction mixture to room temperature, it was diluted with Et$_2$O (50 ml), dried with Na$_2$SO$_4$ and filtered. The solvents were removed under reduced pressure and the remaining crude material was purified by column chromatography (*n*-hexane, R_f = 0.80) to provide the product as colorless oil (81.0 mg, 482 µmol, 41%). – ^{1}H NMR (500 MHz, CDCl$_3$): δ = 2.08 (dt, 2H, ^2J = 6.1 Hz, ^3J = 1.4 Hz, 7´-H), 3.68–3.73 (m, 1H, 1´-H), 3.92–3.97 (m, 1H, 4´-H), 6.80 (dd, 1H, ^3J = 5.1 Hz, ^3J = 3.0 Hz, 5´-H), 6.89–6.91 (m, 2H, 3´-H, 6´-H), 7.18–7.22 (m, 1H, 4-H), 7.30–7.34 (m, 2H, 2-H, 6-H), 7.39–7.43 (m, 2H, 3-H, 5-H). The NMR spectra are in accordance with the literature reports.[4,7]

1,3,5-tri(bicyclo[2.2.1]hepta-2,5-dien-2-yl)benzene

(Ph3NB)

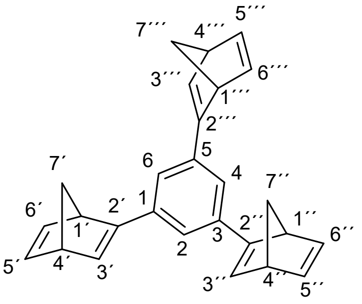

Under anaerobic conditions, an aqueous solution of NaOH (2.7 M, 5.8 mmol, 2.1 ml) was added to a solution of 4,4,5,5-tetramethyl-2-(bicyclo[2.2.1]-heptadien-2-yl)1,3,2-dioxaborolane (501 mg, 2.30 mmol), 1,3,5-tribromobenzene (181 mg, 575 µmol) and Pd(PPh$_3$)$_4$ (66.5 mg, 57.5 µmol) in THF (5.0 ml) and the resulting solution was stirred at 80 °C for 18 h. After cooling the reaction mixture to room temperature, it was diluted with Et$_2$O (50 ml), dried with Na$_2$SO$_4$ and filtered. The solvents were removed under reduced pressure and the remaining crude material was purified by column chromatography (*n*-hexane/EtOAc, 9/1, *v*/*v*, R_f = 0.28) to provide the product as off-white amorphous solid (120 mg, 345 µmol, 60%), mp > 300 °C. – ^{1}H NMR (500 MHz, CDCl$_3$): δ = 2.11 (dt, 3H, ^2J = 6.1 Hz, ^3J = 1.6 Hz, 7´-H, 7´´-H, 7´´´-H), 2.14 (dt, 3H, ^2J = 6.1 Hz, ^3J = 1.6 Hz, 7´-H, 7´´-H, 7´´´-H), 3.70–3.72 (m, 3H, 4´-H, 4´´-H, 4´´´-H), 3.94–3.98 (m, 3H, 1´-H, 1´´-H, 1´´´-H), 6.80 (dd, 3H, ^3J = 5.2 Hz, ^3J = 3.1 Hz, 5´-H, 5´´-H, 5´´´-H), 6.90–6.92 (m, 3H, 6´-H, 6´´-H, 6´´´-H), 6.92 (d, 3H, ^3J = 3´-H, 3´´-H, 3´´´-H), 7.28 (s, 3H, 2-H, 4-H, 6-H). – ^{13}C NMR (125 MHz, CDCl$_3$): δ = 51.0 (C4´, C4´´, C4´´´), 51.7 (C1´, C1´´, C1´´´), 72.7 (C7´, C7´´, C7´´´), 119.9 (C2, C4, C6), 136.4 (C3´, C3´´, C3´´´), 136.6 (C1, C3, C5), 142.2 (C6´, C6´´, C6´´´), 143.6 (C5´, C5´´, C5´´´), 157.0 (C2´, C2´´, C2´´´). – EI. Anal. for $C_{27}H_{24}$: calc. (%): C 93.06, H 6.94. found (%): C 93.33, H 7.08. The analytical data aligns with our previously reported characterization.[6]

1-(bicyclo[2.2.1]hepta-2,5-dien-2-yl)naphthalene

(1NNB)

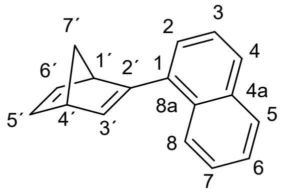

Under anaerobic conditions, an aqueous solution of NaOH (2.7 M, 14 mmol, 5.2 ml) was added to a solution of 4,4,5,5-tetramethyl-2-(bicyclo[2.2.1]-heptadien-2-yl)1,3,2-dioxaborolane (330 mg, 1.51 mmol), 1-bromonaphthalene (284 mg, 1.38 mmol) and $Pd(PPh_3)_4$ (79.6 mg, 68.8 µmol) in THF (5.0 ml) and the resulting solution was stirred at 80 °C for 18 h. After cooling the reaction mixture to room temperature, it was diluted with Et_2O (50 ml), dried with Na_2SO_4 and filtered. The solvents were removed under reduced pressure and the remaining crude material was purified by column chromatography (*n*-hexane, R_f = 0.61) to provide the product as colorless oil (168 mg, 771 µmol, 56%). – ^{1}H NMR (500 MHz, $CDCl_3$): δ = 2.18 (d, 1H, ^3J = 6.0 Hz, 7´-H), 2.39 (d, 1H, ^3J = 6.0 Hz, 7´-H), 3.84–3.85 (m, 2H, 1´-H, 4´-H), 6.83 (d, 1H, ^3J = 3.0 Hz, 3´-H), 6.89 (dd, 1H, ^3J = 5.2 Hz, ^3J = 3.0 Hz, 5´-H), 7.07 (dd, ^3J = 5.1 Hz, ^3J = 3.0 Hz, 6´-H), 7.28 (d, 1H, ^3J = 7.1 Hz, 2-H), 7.42 (dd, 1H, ^3J = ^3J = 8.0 Hz, 3-H), 7.46–7.49 (m, 2H, 6-H, 7´-H), 7.72 (d, 1H, ^3J = 8.2 Hz, 4-H), 7.83–7.86 (m, 1H, 5-H), 8.12–8.15 (m, 1H, 8-H). – ^{13}C-NMR (125 MHz, $CDCl_3$): δ = 51.6 (C4´), 56.2 (C1´), 73.5 (C7´), 123.3 (C2), 125.5 (C3), 125.8 (C6), 125.9 (C7), 126.0 (C8), 127.3 (C4), 128.5 (C5), 131.2 (C8a), 134.0 (C4a), 136.3 (C1), 140.1 (C3´), 142.8 (C6´), 143.4 (C5´), 156.1 (C2´). – EI. Anal. for $C_{17}H_{14}$: calc. (%): C 93.54, H 6.46, found (%): C 93.82, H 6.45. The analytical data aligns with our previously reported characterization.[4,5]

2-(bicyclo[2.2.1]hepta-2,5-dien-2-yl)naphthalene

(2NNB)

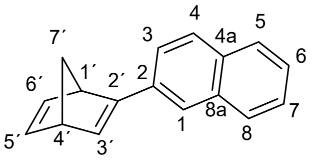

Under anaerobic conditions, an aqueous solution of NaOH (2.7 M, 14 mmol, 5.2 ml) was added to a solution of 4,4,5,5-tetramethyl-2-(bicyclo[2.2.1]-heptadien-2-yl)1,3,2-dioxaborolane (330 mg, 1.51 mmol), 2-bromonaphthalene (284 mg, 1.38 mmol) and $Pd(PPh_3)_4$ (79.6 mg, 68.8 µmol) in THF (5.0 ml) and the resulting solution was stirred at 80 °C for 18 h. After cooling the reaction mixture to room temperature, it was diluted with Et_2O (50 ml), dried with Na_2SO_4 and filtered. The solvents were removed under reduced pressure and the remaining crude material was purified by column chromatography (*n*-hexane, R_f = 0.40) to provide the product as a white amorphous solid (220 mg, 1.00 mmol, 72%); mp. 59–62 °C. – ^{1}H-NMR (500 MHz, $CDCl_3$): δ = 2.16 (dt, 1H, ^2J = 6.2 Hz, ^3J = 1.6 Hz, 7´-H), 2.18 (dt, 1H, ^2J = 6.2 Hz, ^3J = 1.6 Hz, 7´-H), 3.75–3.78 (m, 1H, 4´-H), 4.08–4.11 (m, 1H, 1´-H), 6.82 (dd, 1H, ^3J = 5.0 Hz, ^3J = 3.0 Hz, 5´-H), 6.95 (dd, 1H, ^3J = 5.0 Hz, ^3J = 3.2 Hz, 6´-H), 7.03 (d, 1H, ^3J = 3.2 Hz, 3´-H), 7.38–7.48 (m, 2H, 6´-H, 7-H), 7.57 (dd, 1H, ^3J = 8.6 Hz, ^4J = 1.8 Hz, 3-H), 7.74–7.79 (m, 3H, 1-H, 4-H, 5-H), 7.82–7.84 (m, 1H, 8-H). – ^{13}C-NMR (125 MHz, $CDCl_3$): δ = 51.1 (C4´), 51.5 (C1´), 72.3 (C7´), 122.7 (C1), 124.0 (C3), 125.7 (C6), 126.3 (C7), 127.7 (C5), 128.1 (C4), 128.2 (C8), 132.8 (C4a), 133.8 (C8a), 136.9 (C3´), 139.2 (C2), 142.0 (C6´), 143.6 (C5´), 156.9 (C2´). – EI. Anal. for $C_{17}H_{14}$: calc. (%): C 93.54, H 6.46, found (%): C 93.22, H 6.52. The analytical data aligns with our previously reported characterization.[5]

3. DFT calculations

All computational calculations were performed using the Orca 5.0.4 software package.[8] The B3LYP functional in combination with the 6-311+G(d,p) basis set was employed for all Density-Functional Theory (DFT) calculations. Geometry optimizations were carried out initially, followed by the calculation of

vibration frequencies. Notably, all optimization runs consistently converged to stationary points, with no imaginary frequencies detected, indicating convergence to minimum energy structures.

The lowest triplet states were optimized starting from the respective energy-minimized singlet ground state geometries. Triplet state energies were determined by comparing the energies of the optimized structures of the singlet ground state and lowest triplet state for each compound. These results are summarized in Table 1 of the main manuscript. Additional single-point calculations were performed to obtain spin densities for the optimized triplet states using the Mulliken population analysis method.

To determine electronic excitation energies and oscillator strengths of the 30 lowest transitions, Time-Dependent Density-Functional Theory (TD-DFT) calculations were performed on the previously geometry-optimized structures.

The computational results were analyzed and visualized using the Avogadro 1.2.0 software tool,[9] which facilitated the depiction of molecular structures, orbital distributions, and spin densities. In the depiction of spin densities, blue-colored surfaces denote positive spin densities, whereas red-colored surfaces denote negative spin densities.

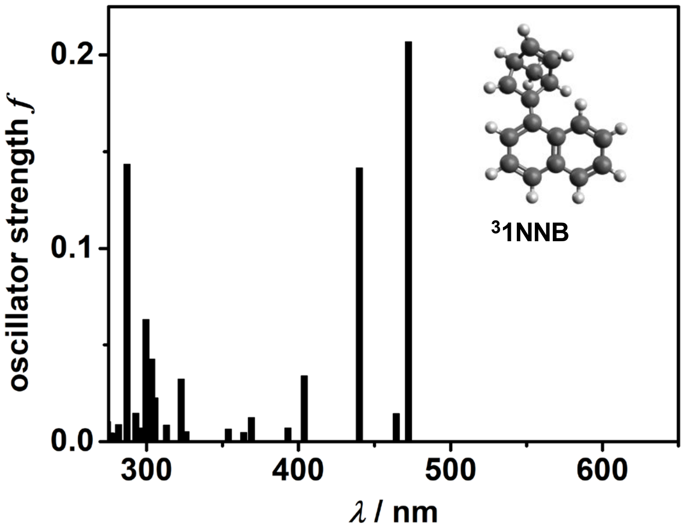

Figure S2: Predicted energies and oscillator strengths of electronic transitions for the triplet state of 1NNB.

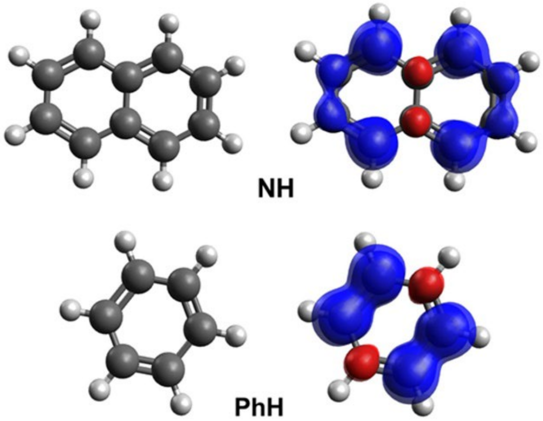

Figure S3: (Left) Structures of the geometry-optimized triplet states of the reference compounds benzene (PhH) and naphthalene (NH). (Right) The corresponding spin densities (iso value: 0.005 a.u.; total sum of atomic spin populations: 2.000).

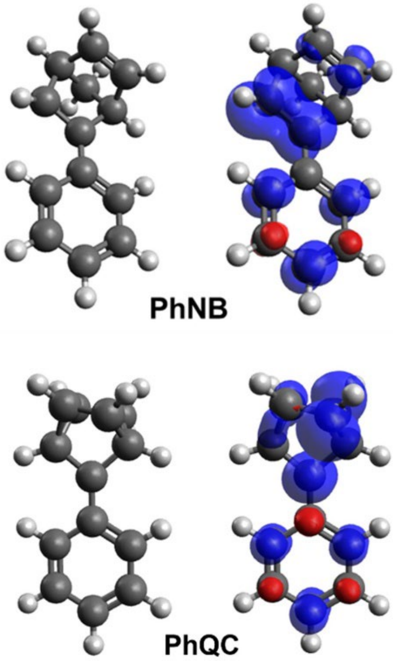

Figure S4: (Left) Structures of the geometry-optimized triplet states of PhNB and PhQC. (Right) The corresponding spin densities (iso value: 0.005 a.u.; total sum of atomic spin populations: 2.000). Bond cleavage during optimization (one bond within the QC moiety).

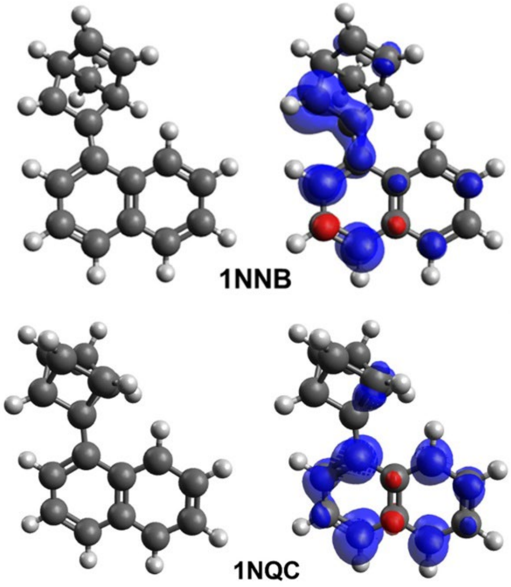

Figure S5: (Left) Structures of the geometry-optimized triplet states of 1NNB and 1NQC. (Right) The corresponding spin densities (iso value: 0.005 a.u.; total sum of atomic spin populations: 2.000).

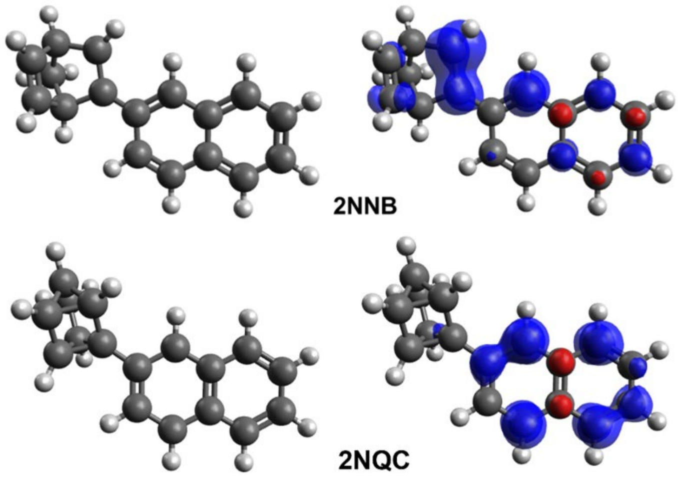

Figure S6: (Left) Structures of the geometry-optimized triplet states of 2NNB and 2NQC. (Right) The corresponding spin densities (iso value: 0.005 a.u.; total sum of atomic spin populations: 2.000).

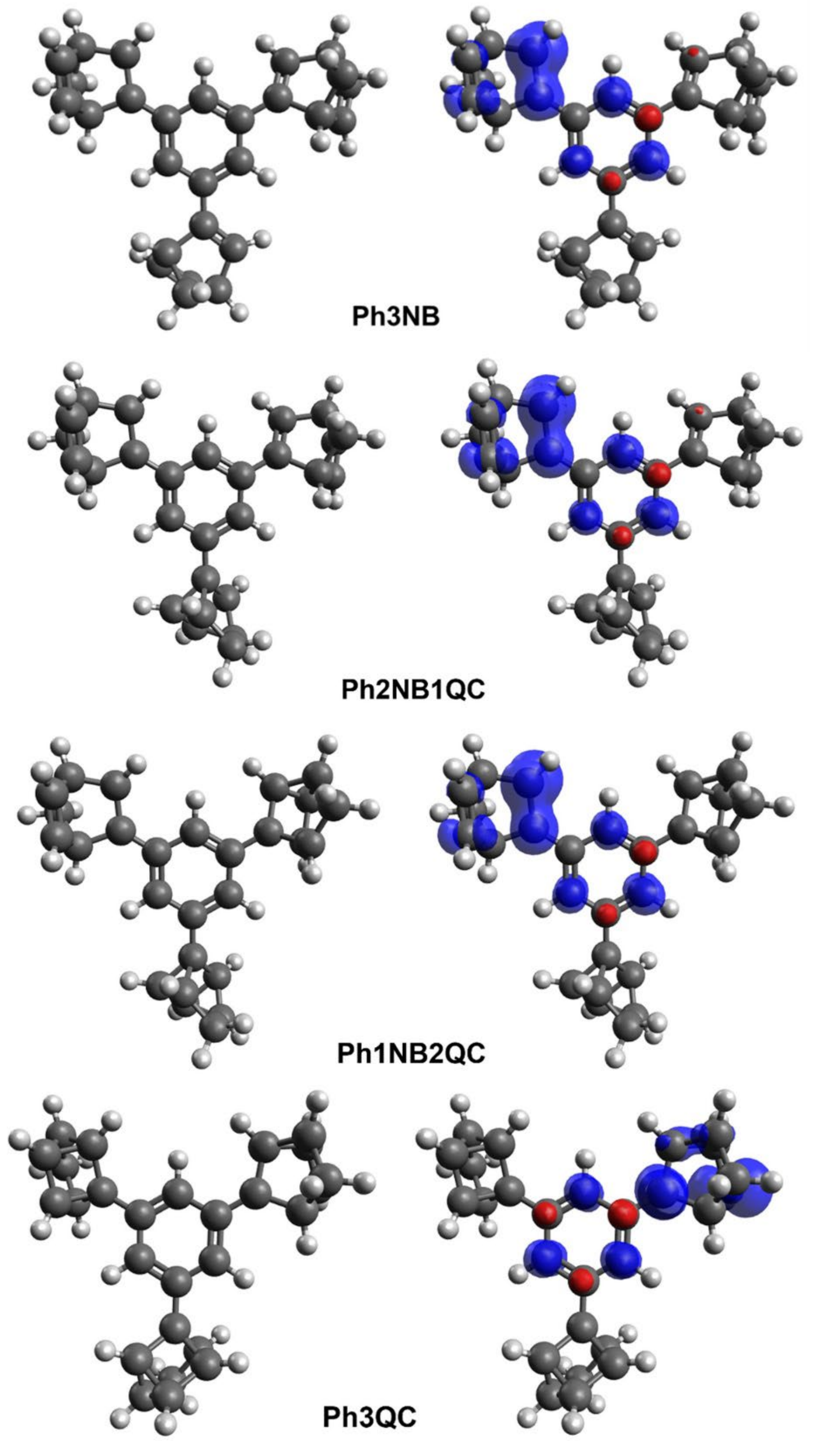

Figure S7: (Left) Structures of the geometry-optimized triplet states of Ph3NB, Ph2NB1QC, Ph1NB2QC and Ph3QC. (Right) The corresponding spin densities (iso value: 0.005 a.u.; total sum of atomic spin populations: 2.000).

4. Stern–Volmer experiments

4.1. Triplet-triplet energy transfer studies of PhNB, 1NNB and 2NNB with various metal sensitizers

Table S1: Triplet energy transfer rates of selected metal sensitizers to the norbornadienes arranged by decreasing triplet energy. The energy transfer rates were determined by Stern–Volmer analyses (see below).

Sensitizer	τ_0 (ns)	E_T (eV)	k_{EnT} (10^9 M^{-1} s^{-1})		
			PhNB	1NNB	2NNB
$Ir(dFppy)_3$	1940	2.75 [b]	3.72	4.87	6.30
Ir(*p*-F-ppy)$_3$	2000	2.71 [b]	3.27	4.89	6.23
$Ir(ppy)_3$	1790	2.52 [b]	0.91	3.10	3.80
$[Ir(ppy)_2(phen)]^+$	790	2.38 [b]	0.13	0.74	1.05
$[Ru(phen)_3]^{2+}$	450-510 [a]	2.19 [c]	0.079	0.59	0.83
$[Ru(bpy)_3]^{2+}$	950	2.12 [c]	0.012	0.13	0.19
$[Os(phen)_3]^{2+}$	230	1.80 [d]	–	0.031	0.044

[a] The triplet lifetime of $[Ru(phen)_3]^{2+}$ depends strongly on the temperature see Chapter S7 for details. [b] taken from reference[10]. [c] Taken from reference[11]. [d] Taken from reference[12].

A series of similar metal sensitizers was used to study the triplet energy transfer step to 1NNB, 2NNB and PhNB. The deactivation of the sensitizers with increasing quencher concentration of the norbornadienes was monitored by TCSPC or LFP (see Figure S8-S10). TCSPC and LFP measurements for a given quenching process gave the same result regardless of the method as several control experiments established. A Stern–Volmer analysis yielded the triplet energy transfer rates (Table S1). Applying the Sandros-equation to model the data would be inadequate. Balzani and colleagues have pointed out that this equation fails to consider energy transfer from and to distorted excited states as well as the potential for rates below diffusion in the exergonic area.[13] Indeed, the geometries of the excited states of norbornadienes are described to be highly distorted,[14–16] which in our case is also supported by the geometry-optimized structures of the triplet-excited states (see Chapter S2). Based on this, we rather estimate the triplet energy using an exponential fit function with the EnT rate limit as the upper boundary (Figure 4, main paper). The energy transfer rate limit does not only depend on the diffusion limit of the solvent[17] but also on the size of the sensitizer and quencher as well as spatial overlap of the involved orbitals.[10] When the triplet energy of the sensitizer equals that of the quencher ($\Delta E_{\mathrm{T}} = 0$) the energy transfer rate is set as exactly half of the energy transfer limit.[17] This method yielded energies of 2.39 eV for 1NNB, 2.41 eV for 2NNB and 2.56 eV for PhNB, respectively.

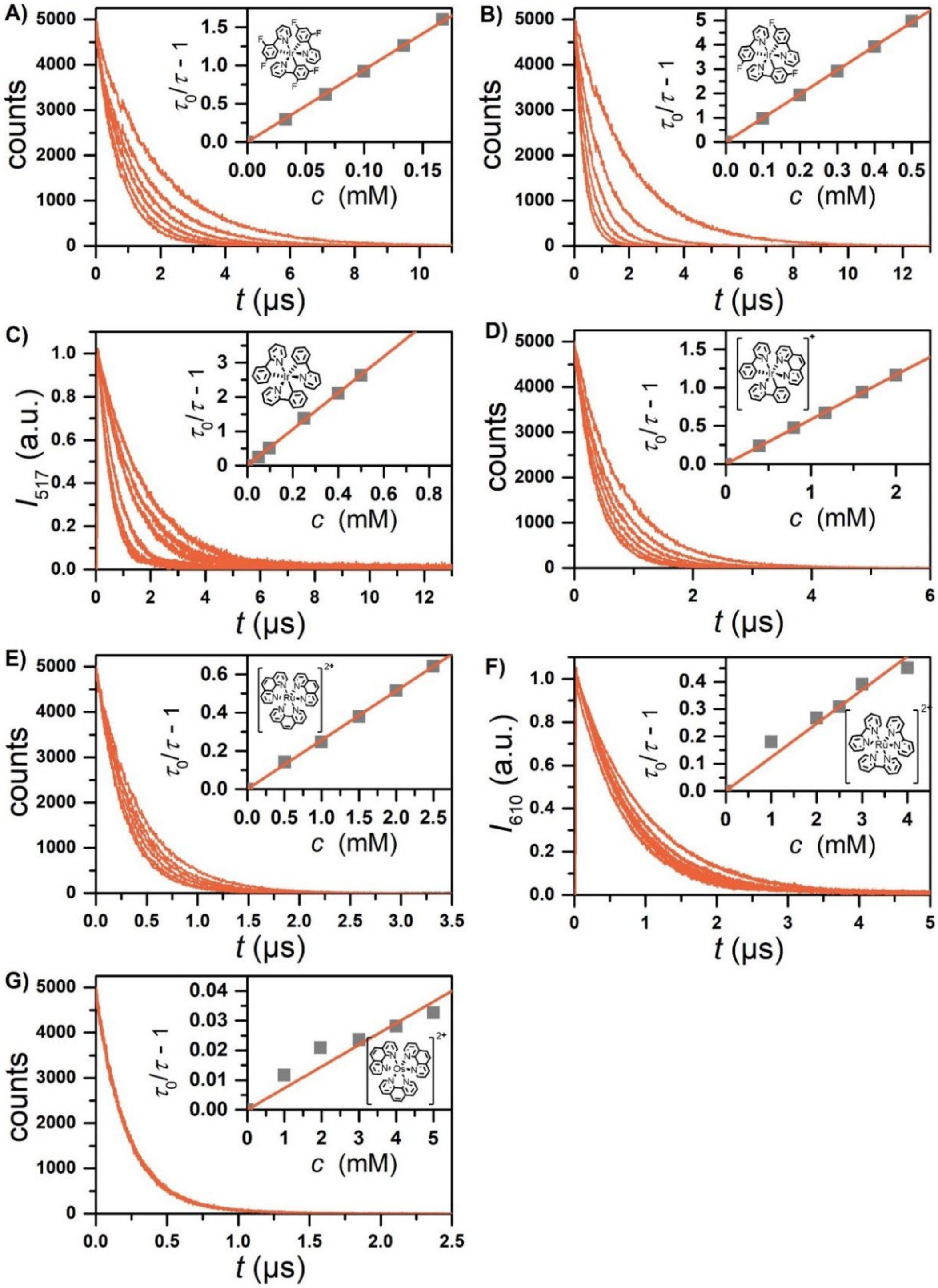

Figure S8: Time-resolved emission quenching experiments with various metal sensitizers and different concentrations of 1NNB in Ar-saturated MeCN. The concentration of the sensitizer was adjusted to OD ~ 0.2 at the excitation wavelength (446.4 nm using the TCSPC instrument or 355 nm and 532 nm using the LFP setup). The insets display the corresponding Stern–Volmer plot. A) Ir(dFppy)$_3$ (90 μM), B) Ir(*p*-F-ppy)$_3$ (90 μM), C) Ir(ppy)$_3$ (20 μM), D) [Ir(ppy)$_2$(phen)]$^+$ (85 μM), E) [Ru(phen)$_3$]$^{2+}$ (15 μM), F) [Ru(bpy)$_3$]$^{2+}$ (200 μM), G) [Os(phen)$_3$]$^{2+}$ (15 μM).

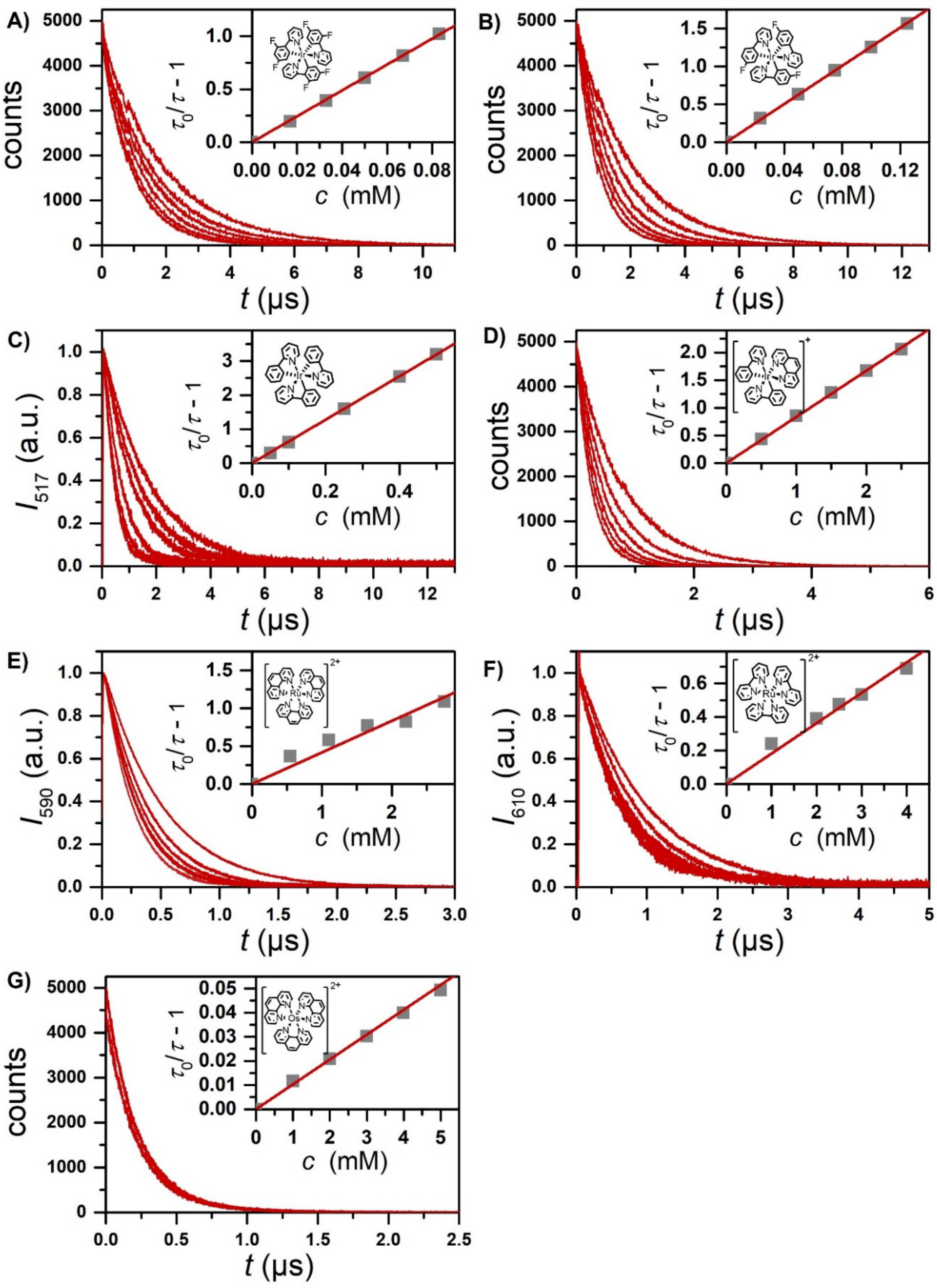

Figure S9: Time-resolved emission quenching experiments with various metal sensitizers and different concentrations of 2NNB in Ar-saturated MeCN. The concentration of the sensitizer was adjusted to OD ~ 0.2 at the excitation wavelength (446.4 nm using the TCSPC instrument or 355 nm and 532 nm using the LFP-setup). The insets display the corresponding Stern−Volmer plot. A) Ir(dFppy)$_3$ (90 µM), B) Ir(*p*-F-ppy)$_3$ (90 µM), C) Ir(ppy)$_3$ (20 µM), D) [Ir(ppy)$_2$(phen)]$^+$ (85 µM), E) [Ru(phen)$_3$]$^{2+}$ (100 µM), F) [Ru(bpy)$_3$]$^{2+}$ (150 µM), G) [Os(phen)$_3$]$^{2+}$ (15 µM).

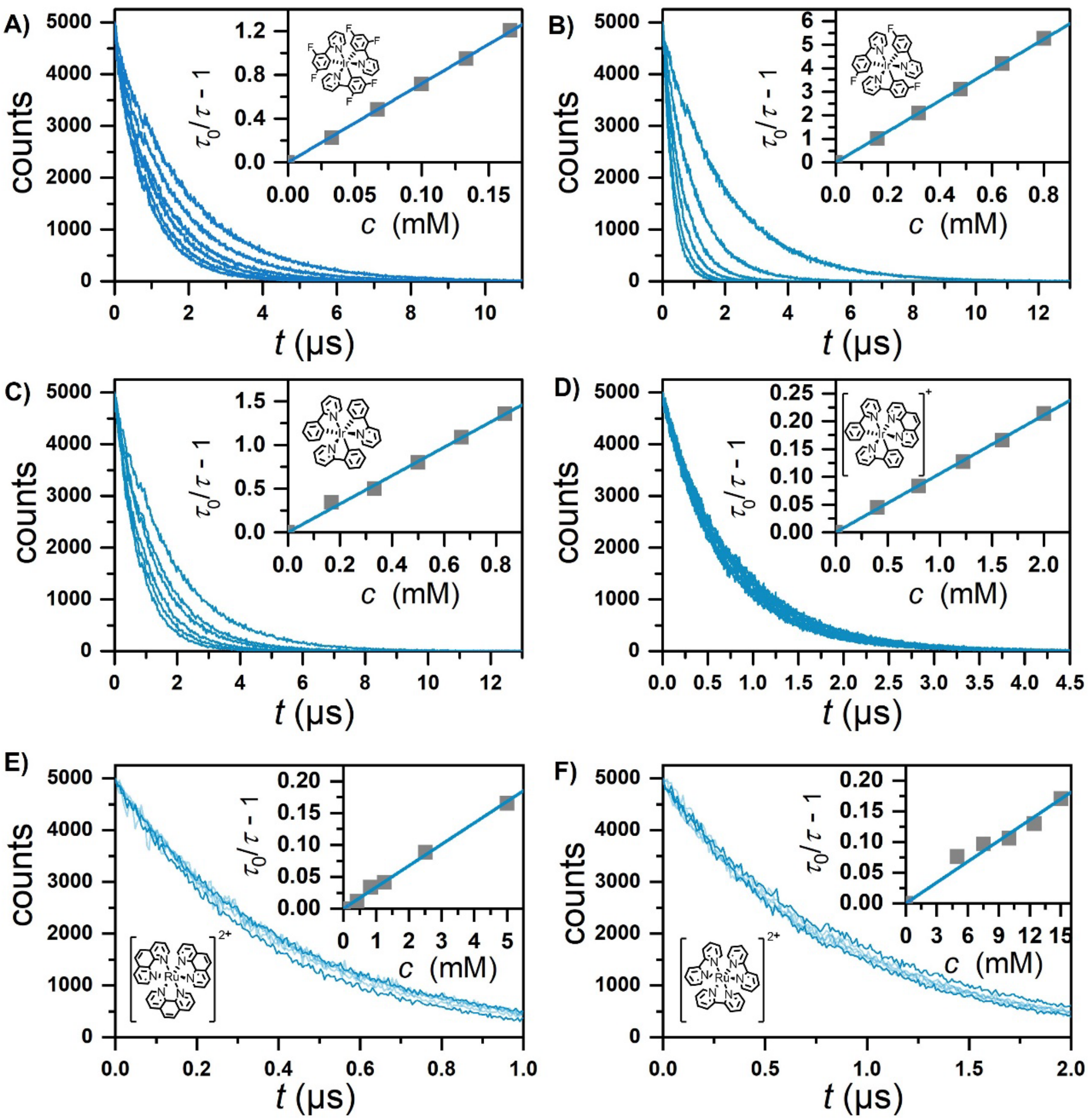

Figure S10: Time-resolved emission quenching experiments with various metal sensitizers and different concentrations of PhNB in Ar-saturated MeCN. The concentration of the sensitizer was adjusted to $OD_{446.5}$ ~ 0.2 at the excitation wavelength. The insets display the corresponding Stern–Volmer plot. A) $Ir(dFppy)_3$ (90 µM), B) Ir(*p*-F-ppy)$_3$ (90 µM), C) $Ir(ppy)_3$ (15 µM), D) $[Ir(ppy)_2(phen)]^+$ (85 µM), E) $[Ru(phen)_3]^{2+}$ (15 µM), F) $[Ru(bpy)_3]^{2+}$ (20 µM).

4.2. Additional triplet energy transfer studies of various sensitizers and triplet acceptors

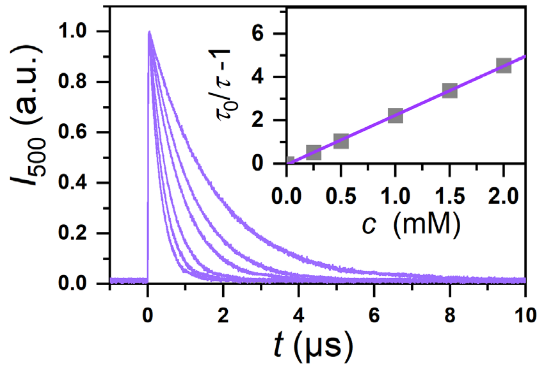

Figure S11: Time-resolved emission of a solution of 20 μM Ir(ppy)$_3$ in Ar-saturated MeCN with increasing concentrations of (*E*)-ethyl cinnamate upon excitation at 355 nm. Inset: corresponding Stern–Volmer plot.

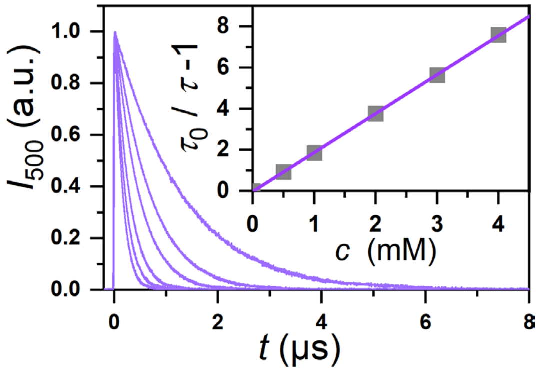

Figure S12: Time-resolved emission of a solution of 20 μM Ir(ppy)$_3$ in Ar-saturated toluene with increasing concentrations of (*E*)-ethyl cinnamate upon excitation at 355 nm. Inset: corresponding Stern–Volmer plot.

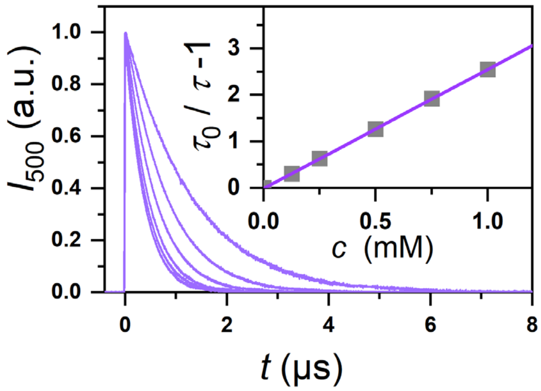

Figure S13: Time-resolved emission of a solution of 20 μM Ir(ppy)$_3$ in Ar-saturated toluene with increasing concentrations of Ph3NB upon excitation at 355 nm. Inset: corresponding Stern–Volmer plot.

4.3. Kinetic simulations of the energy transfer efficiency

The influence of the sensitizer lifetime, the quencher concentration and the energy transfer rate on the energy transfer efficiency can be rationalized by equation (6). By specifying the quencher concentration and triplet sensitizer lifetime (unquenched) we simulated the energy transfer efficiency against the triplet energy transfer rate (Figure S14A). A common concentration of 100 mM for lab-scale irradiation experiments and triplet sensitizer lifetimes typical for organic chromophores (100 µs – 1 µs) and photoactive metal complexes (10 µs – 100 ns) were selected.

$$\eta_{\mathrm{EnT}} = 1 - \frac{1}{1 + c_0 \cdot \tau_0 \cdot k_{\mathrm{EnT}}} \tag{6}$$

Furthermore, the triplet energy transfer efficiencies for the specific sensitizer-photoswitch pairs RuPhen–1NNB, OsPhen–1NNB and Ir(ppy)$_3$–Ph3NB were simulated using the experimental unquenched sensitizer lifetimes and triplet energy transfer rate constants (Figure S14B). This simulation was performed for different 1NNB/Ph3NB concentrations ranging from 1 to 1000 mM, according to equation (6).

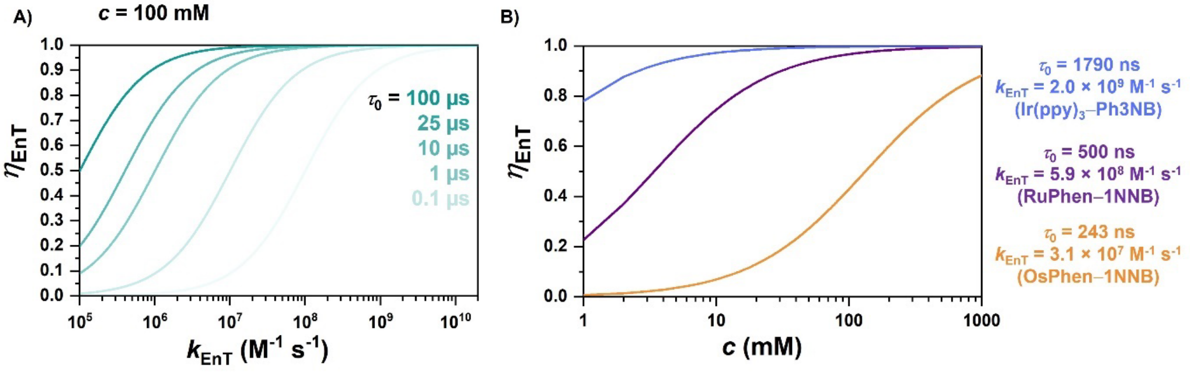

Figure S14: Simulated energy transfer efficiencies according to equation (6). A) Against the energy transfer rate with a quencher concentration of 100 mM and sensitizer lifetimes ranging from 100 µs to 100 ns. B) Against the quencher concentration for specific sensitizer–photoswitch pairs.

5. Interconversion quantum efficiency of 1NNB to 1NQC determined by relative actinometry

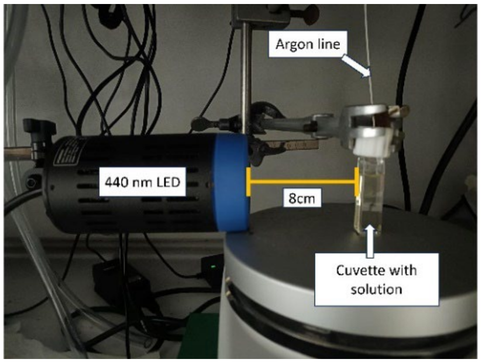

Figure S15: Experimental setup used to determine the interconversion efficiency.

The triplet-sensitized photoisomerization yield of 1NNB to 1NQC was determined by a relative actinometry method. Under identical conditions the interconversion of 1NNB to 1NQC was compared to the sensitized photoisomerization of a reference compound ((*E*)-ethyl cinnamate, *E*-EC) with a known isomerization efficiency (η_{iso} = 0.5 to the *Z* isomer).[10,18] Two solutions each containing 23.4 µM Ir(ppy)$_3$ (used as sensitizer), 2.53 mM dimethyl sulfone (DMS) (used as internal standard), and either 29.1 mM 1NNB or 30.5 mM *E*-EC in Ar-saturated MeCN-d$_3$ (4 mL) were prepared. The selected concentrations of 1NNB and *E*-EC are high enough to ensure near quantitative quenching of ^{3}Ir(ppy)$_3$ (compare quenching constants in Chapter S4 and Table S1). Each solution was transferred

to a cuvette and irradiated with a 440 nm Kessil LED at 25% intensity at a constant distance of 8 cm (see Figure S15 for the experimental setup).

This ensures that the photon flux is consistent for both photoreactions. At several time intervals 0.5 mL were removed and used to monitor the photoisomerization of 1NNB and *E*-EC by ^{1}H NMR spectroscopy. Selected NMR signals of 1NNB and 1NQC (Figure S17) and *E*-EC and the respective isomer *Z*-EC (Figure S18) were used to determine the isomer composition (for complete NMR spectra see Figure S43 and Figure S44). The integrated signals were referenced to the internal standard (DMS, 6H at 2.91 ppm) allowing us to calculate the total and isomer concentrations of 1NNB/1NQC and *E*-EC/*Z*-EC (Figure S16). The irradiation experiment was stopped when >15% (and <20%) of the product isomer was formed ensuring a linear relationship and a sufficient sensitivity. A linear fit yielded the relative reactions rates. Since exactly the same concentration of the sensitizer $Ir(ppy)_3$ was employed, we can disregard the sensitizer's properties such as spectral overlap and intersystem crossing quantum yield for this relative comparison. According to equation 7, the number of absorbed photons (*NAP*) of this specific setup can be calculated for the reference system from the observed reaction rate. Rearranging equation 7 allows us to determine an interconversion efficiency, η_{iso}, of 1.01 ± 0.06 for 1NNB. An efficiency over 1 is not reasonable as e.g. chain reactions cannot be expected for this transformation. Hence, we conclude that practically quantitative conversion of triplet-excited 1NNB is achieved in line with literature reports on the sensitization of norbornadiene and derivatives.[19,20]

$$NAP = \frac{rate}{\eta_{\mathrm{TTET}} \cdot \eta_{\mathrm{iso}}} \tag{7}$$

Table S2: Photochemical properties of 1NNB and *E*-EC involved in triplet energy transfer driven photoisomerization with $Ir(ppy)_3$ using a 440 nm Kessil lamp for sensitizer excitation.

Quencher	$k_q(Ir(ppy)_3)$ (10^9 M^{-1} s^{-1})	$\eta_{TTET}(Ir(ppy)_3)$[a]	η_{iso}	rate (mM s^{-1})	*NAP* (mM s^{-1})
1NNB	3.10	0.993	1.01 ± 0.06	$(3.31 \pm 0.05)\cdot10^{-2}$	$(3.28 \pm 0.04)\cdot10^{-2}$
E-EC	1.35	0.986	0.5[10,18]	$(1.62 \pm 0.02)\cdot10^{-2}$	

[a] At selected concentration (29.1 mM 1NNB, 30.5 mM *E*-EC).

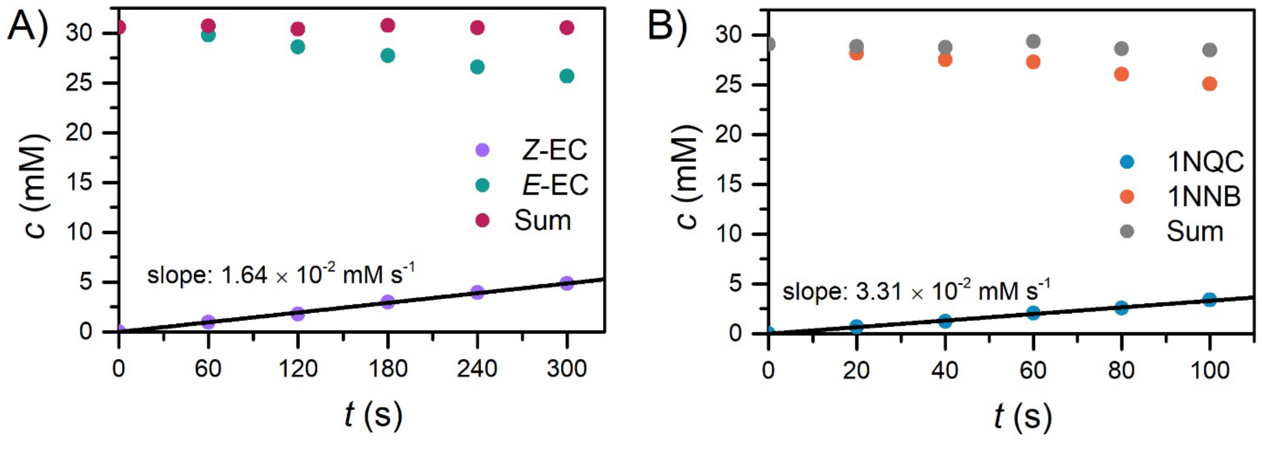

Figure S16: Isomer and total concentrations of two solutions containing each 23.4 µM $Ir(ppy)_3$, 2.5 mM dimethyl sulfone (DMS), and either 30.5 mM *E*-EC (A) or 29.1 mM 1NNB in Ar-saturated MeCN-d_3 (4 mL) given at different irradiation time intervals. A 440 nm LED from Kessil (25% intensity, 8 cm distance) was used as excitation light source. The concentrations were determined by NMR spectroscopy (see text for details).

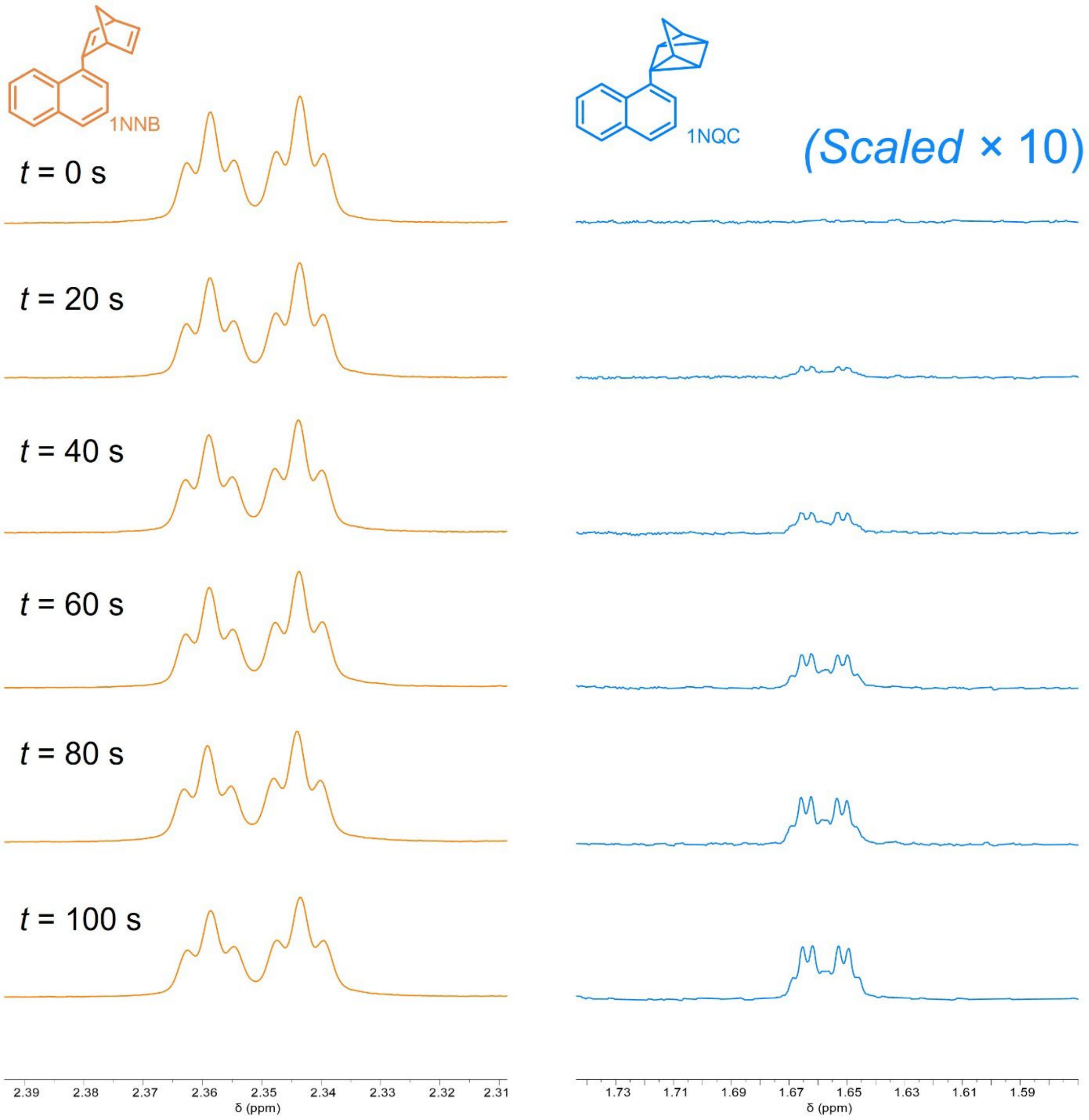

Figure S17: ^{1}H NMR spectra with selected spectral range of an Ar-saturated solution MeCN-d_3 containing 23.4 µM $Ir(ppy)_3$, 29.1 mM 1NNB, and 2.53 mM DMS. The spectra were recorded during irradiation with a 440 nm LED (set to 25%, 8 cm distance) with intervals of 20 s. The displayed signals, left corresponding to 1NNB (1H) and right to 1NQC (1H), were used to determine the interconversion efficiency. The complete ^{1}H NMR spectrum is displayed in Figure S43.

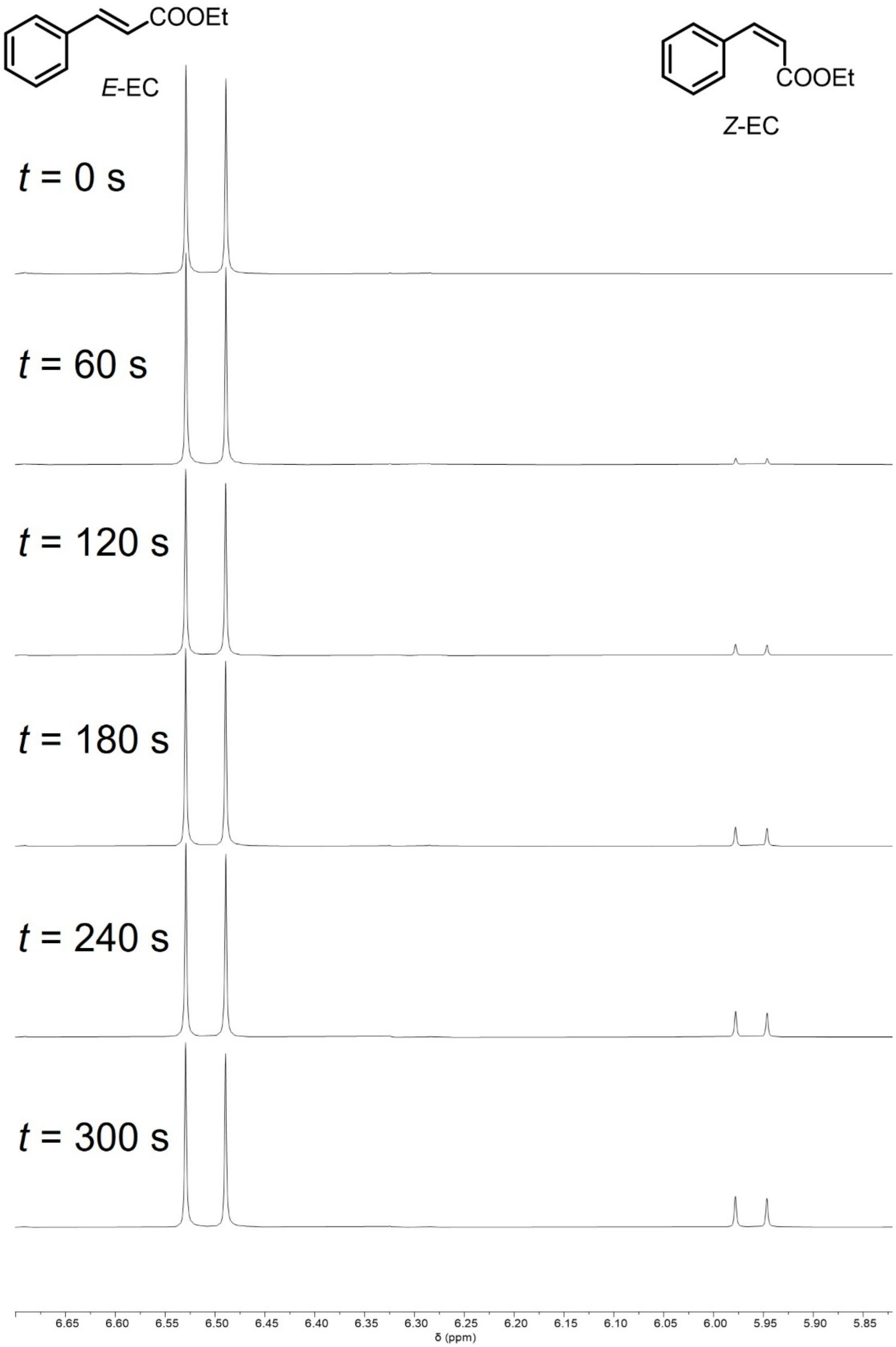

Figure S18: ^{1}H NMR spectra with selected spectral range of an Ar-saturated MeCN-d_3 solution containing 23.4 µM Ir(ppy)$_3$, 30.5 mM *E*-EC, and 2.53 mM DMS. The spectra were recorded during irradiation with a 440 nm LED (set to 25%, 8 cm distance) with intervals of 60 s. The displayed doublet signals, left (6.52 ppm, 1H) corresponding to *E*-EC and right (5.96 ppm, 1H) to *Z*-EC, were used to determine the number of absorbed photons of our setup, finally yielding the interconversion efficiency of 1NNB. The complete ^{1}H NMR spectrum is displayed in Figure S44.

6. Interconversion quantum efficiency of Ph3NB to Ph2NB1QC determined by relative actinometry

The triplet-sensitized photoisomerization yield of Ph3NB to Ph2NB1QC was determined by relative actinometry method analogous to 1NNB (Chapter S5). Under identical irradiation conditions (440 nm) the interconversion of Ph3NB to Ph2NB1QC was compared to the sensitized photoisomerization of (*E*)-ethyl cinnamate) with a known isomerization efficiency (η_{iso} = 0.5 to the *Z* isomer). Two solutions each containing 23.4 µM Ir(ppy)$_3$ (used as sensitizer), 10.0 mM dioxan (used as internal standard), and either 20.0 mM Ph3NB or 30.0 mM (*E*)-ethyl cinnamate (*E*-EC) in Ar-saturated toluene-d$_8$ (4 mL) were prepared. The selected concentration of *E*-EC is high enough to ensure near quantitative quenching of ^{3}Ir(ppy)$_3$ (compare quenching constants in Chapter S4). The number of absorbed photons of this measurement is lower than for the measurement involving 1NNB. This is due to the lower molar extinction coefficient of Ir(ppy)$_3$ in toluene-d$_8$ than in MeCN-d$_3$. The ^{1}H NMR signals of the NB units are virtually identical in the different isomers, and the same issue occurs for the QC signals, complicating an individual analysis of all isomer concentrations. Therefore, we can only observe the conversion of all three norbornadiene signals to quadricyclane signals simultaneously. We obtain an interconversion efficiency of 0.35 ± 0.02 for Ph3NB, normalizing this value by the number of norbornadiene units (assuming that at the low conversion during our quantitative irradiation experiments only Ph2NB1QC is formed) yields an efficiency of 1.05 ± 0.06 which we assign to the first conversion (Ph3NB to Ph2NB1QC).[19,20]

Table S3: Photochemical properties of Ph3NB and *E*-EC involved in triplet energy transfer driven photoisomerization with Ir(ppy)$_3$ using a 440 nm Kessil lamp for sensitizer excitation.

Quencher	k_q(Ir(ppy)$_3$) (10^9 M^{-1} s^{-1})	η_{TTET}(Ir(ppy)$_3$)[a]	η_{iso}	rate (mM s^{-1})	*NAP* (mM s^{-1})
Ph3NB	2.00	0.987	0.35 ± 0.02 (1.05 ± 0.06)[b]	(0.84 ± 0.02)·10^{-2}	(2.38 ± 0.02)·10^{-2}
E-EC	1.48	0.983	0.5[10,18]	(1.19 ± 0.01)·10^{-2}	

[a] At selected concentration (20.0 mM Ph3NB, 30.0 mM *E*-EC). [b] Normalized to the first conversion (Ph3NB to Ph2NB1QC).

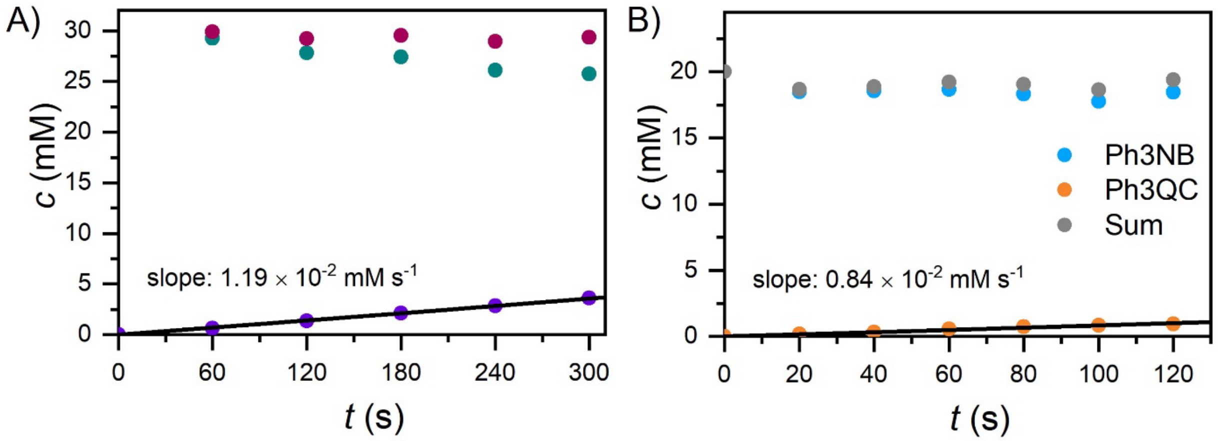

Figure S19: Isomer and total concentrations of two solutions containing each 23.0 µM Ir(ppy)$_3$, 10.0 mM dioxan, and either 30.0 mM *E*-EC (A) or 20.0 mM Ph3NB in Ar-saturated toluene-d$_8$ (4 mL) given at different irradiation time intervals. A 440 nm LED from Kessil (25% intensity, 8 cm distance) was used as excitation light source. The concentrations were determined by ^{1}H NMR spectroscopy (see text for details).

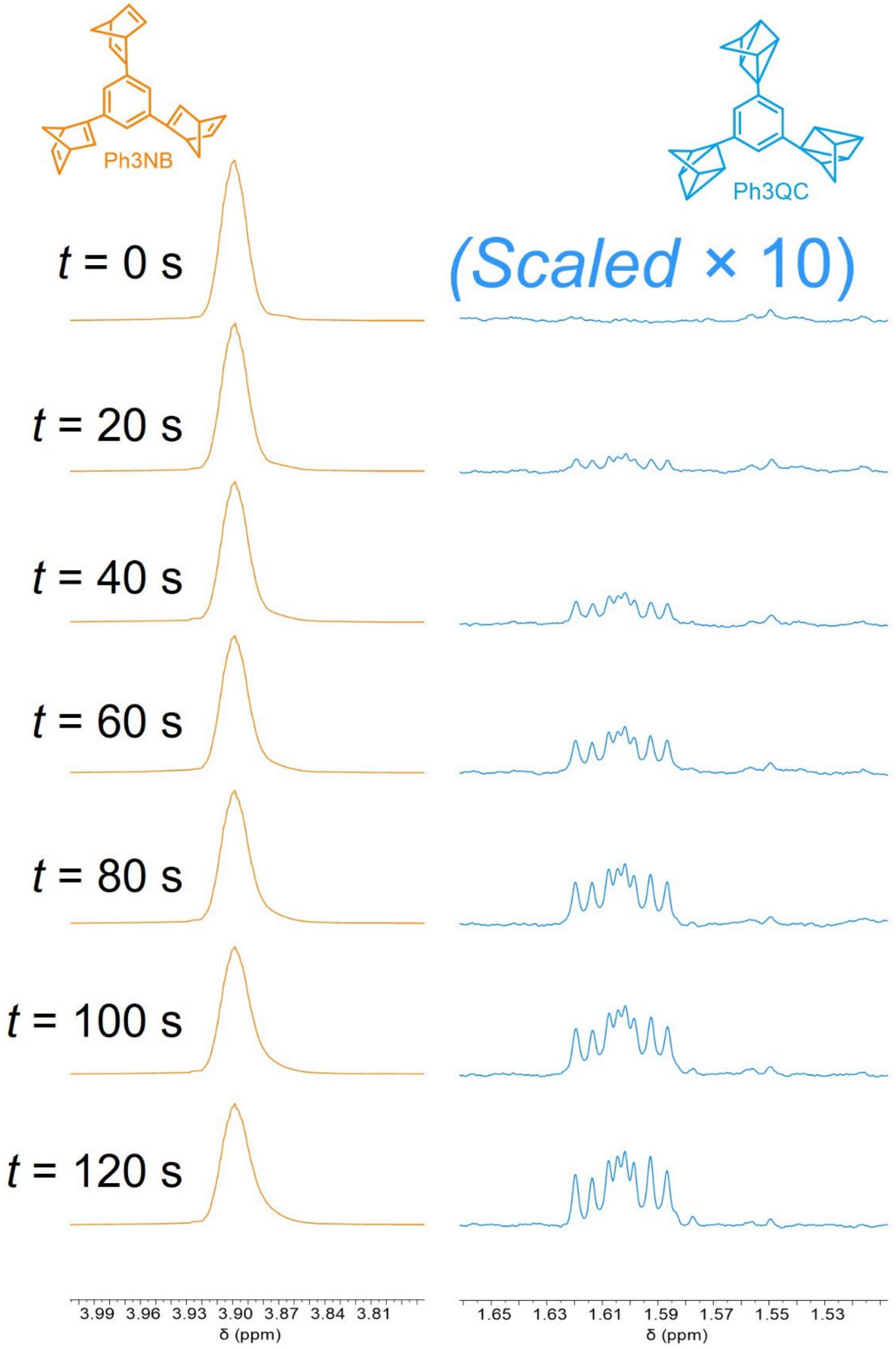

Figure S20: ^{1}H NMR spectra with selected spectral range of an Ar-saturated toluene-d_8 solution containing 23.0 µM Ir(ppy)$_3$, 20.0 mM Ph3NB, and 10.0 mM dioxan. The spectra were recorded during irradiation with a 440 nm LED (set to 25%, 8 cm distance) with intervals of 20 s. The displayed signals, left corresponding to Ph3NB (3H) and right to Ph3QC (3H), were used to determine the interconversion efficiency. The complete ^{1}H NMR spectrum is displayed in Figure S46. See text for further explanations.

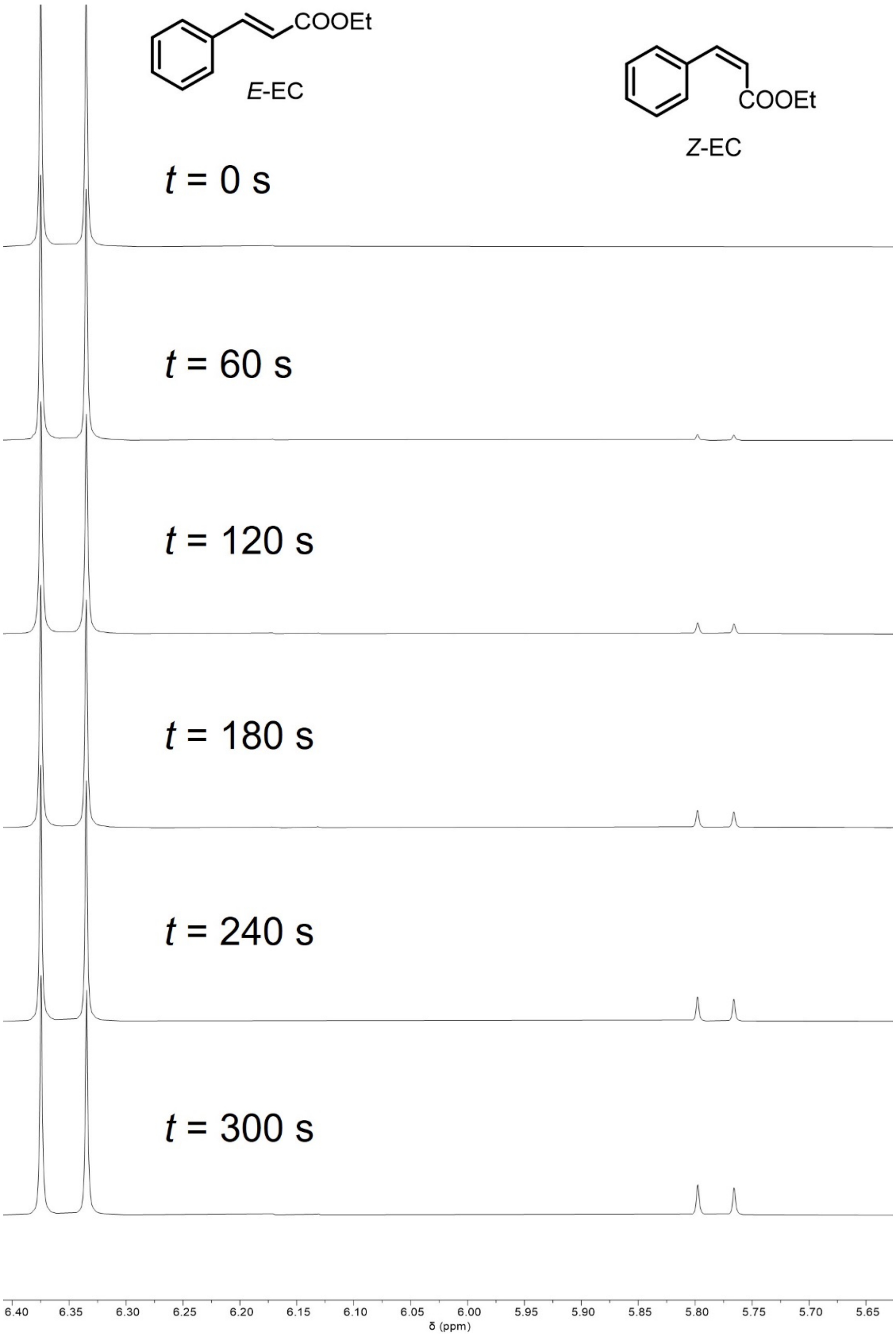

Figure S21: ^{1}H NMR spectra with selected spectral range of an Ar-saturated toluene-d$_8$ solution containing 23 µM Ir(ppy)$_3$, 30.0 mM *E*-EC, and 10 mM dioxan. The spectra were recorded during irradiation with a 440 nm LED (set to 25%, 8 cm distance) with intervals of 60 s. The displayed doublet signals, left (6.35 ppm, 1H) corresponding to *E*-EC and right (5.78 ppm, 1H) to *Z*-EC, were used to determine the number of absorbed photons of our setup, finally yielding the interconversion efficiency of Ph3NB. The complete ^{1}H NMR spectrum is displayed in Figure S45.

7. Maximum energy storage efficiency of the Ir(ppy)3–Ph3NB sensitizer–photoswitch pair

Based on the Ir(ppy)3–Ph3NB sensitizer–photoswitch pair we calculated the maximum energy storage efficiency, η_{MOST}, with equation (8).[21,22]

$$\eta_{\mathrm{MOST}} = \frac{\int_0^{\lambda_{\mathrm{onset}}} \frac{E_{\mathrm{AM1.5}}(\lambda)\cdot(1-T(c,\lambda))\cdot\Phi\cdot\Delta H_{\mathrm{stored}}}{3\cdot h\nu\cdot N_{\mathrm{A}}}\cdot d\lambda}{E_{\mathrm{AM1.5}}} \tag{8}$$

Where $E_{\mathrm{AM1.5}}(\lambda)$ is the spectral irradiance in J s^{-1} m^{-2} nm^{-1}, as reported by the American Society for Testing and Materials (ASTM)[23], T is the transmittance of Ir(ppy)3 (with the reasonable assumption that Ph3NB does not significantly contribute to the overall absorption of solar photons, see below), Φ is the overall quantum efficiency of the sensitized conversion of Ph3NB, $\Delta H_{\mathrm{stored}}$ is the stored energy of Ph3NB and equals 255.8 kJ mol^{-1}, h is the Planck constant in J s, ν is the frequency of the light in s^{-1}, N_{A} is the Avogadro constant and $E_{\mathrm{AM1.5}}$ is the integral over the complete solar irradiance. We simulate the efficiency for a device with the following specifications: 100 mM Ph3NB, 590 µM Ir(ppy)3 (corresponding to the solubility limit), 1 cm path length. The onset absorption wavelength is 523 nm. The overall quantum yield, Φ, is defined in the main paper as follows:

$$\Phi = \Phi_{\mathrm{ISC}}\cdot\eta_{\mathrm{EnT}}\cdot\Phi_{\mathrm{NB}\rightarrow\mathrm{QC}} \tag{9}$$

Ir(ppy)3 efficiently produces the triplet state upon excitation, with Φ_{ISC} = 1.[24] For this particular system η_{EnT} is simulated in Figure S14B, demonstrating that it already exceeds 0.97 at a Ph3NB concentration of 10 mM and approaches unity at 100 mM. The interconversion quantum efficiency is determined in Chapter S6 as $\Phi_{\mathrm{NB}\rightarrow\mathrm{QC}}$ = 1. Therefore, in this scenario, Φ is equal to 1. Since each absorbed photon results in the conversion of only one NB unit, the integral has to be divided by the number of NB units (3). The integrals are visualized in Figure S22 and yield an efficiency limit of η_{MOST} = 5.8%.

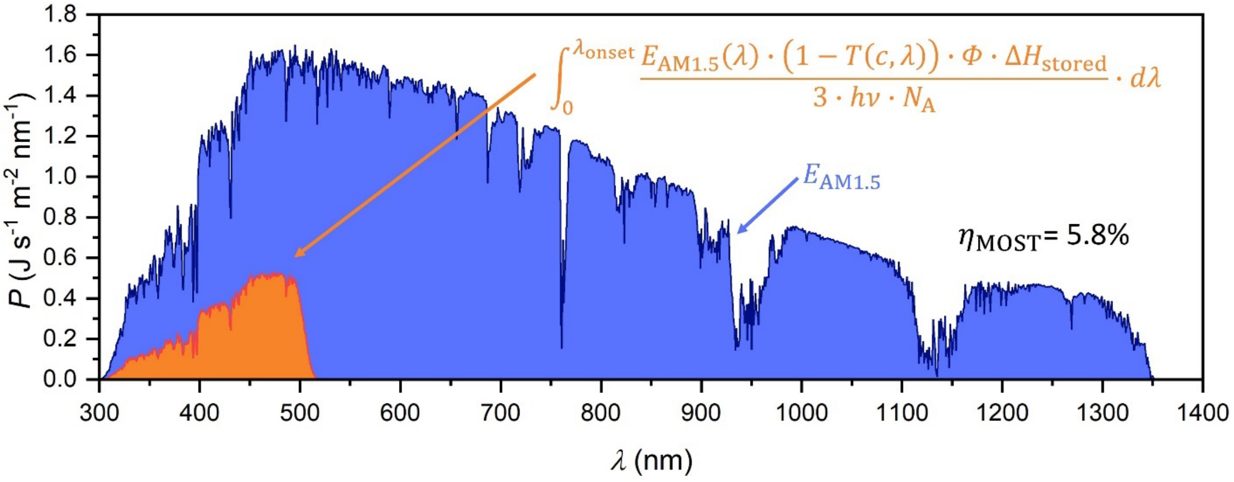

Figure S22: Solar conversion efficiency of the Ir(ppy)3–Ph3NB sensitizer–photoswitch pair. Integrals of equation (8) are visualized. For clarity, the graph is only plotted until 1400 nm, the integration was performed over the complete solar spectrum (up to 4000 nm).

Similar calculations were conducted for direct excitation of Ph3NB at a concentration of 100 mM in the absence of a sensitizer. Under the same parameters as before, we used an onset absorption wavelength

of 390 nm (see Figure 1C, main paper). With a conversion quantum yield after direct excitation of Φ_{iso} = 0.18 (normalized to the number of NB units)[6] we calculated an efficiency of η_{MOST} = 0.53%. This efficiency is an order of magnitude lower than what is achieved through triplet sensitization.

It is important to note that for direct excitation, this efficiency represents only an upper limit. It does not account for the re-excitation of Ph3QC or the decreasing absorption as the conversion to Ph3QC progresses. These issues are avoided with triplet sensitization. Re-sensitization of Ph3QC does not occur due to its significantly higher triplet energy. Additionally, the absorption of Ir(ppy)$_3$ remains unaffected by the concentrations of Ph3NB and Ph3QC.

We estimated the charging time for this specific MOST device using equation (10), which simply compares the number of MOST molecules and the available number of photons that initiate photoswitching. Parameter *d* is the path length, and *PF* is the solar photon flux in E s^{-1} m^{-2} nm^{-1}. The overall solar photon flux is 0.00714 E s^{-1} m^{-2} while only 0.000685 E s^{-1} m^{-2} can be absorbed by Ir(ppy)$_3$ with a path length *d* of 1 cm and a concentration of 590 µM. This corresponds to only 9.6% of the solar photon flux. The concentration of Ph3NB, c_{Ph3NB}, is multiplied by a factor of three accounting for the norbornadiene units for each molecule. In this calculation, we assume that for a given surface area, a specific amount of substance is present, with each norbornadiene unit being converted per absorbed photon.

$$t = \frac{3c_{\mathrm{Ph3NB}} \cdot d}{\int_0^{\lambda_{\mathrm{onset}}} PF \cdot (1 - T(c,\lambda))\, d\lambda \cdot \Phi} = \frac{3 \cdot 90\ \mathrm{mol} \cdot \mathrm{m}^{-3} \cdot 0.01\ \mathrm{m}}{0.000685\ \mathrm{E} \cdot \mathrm{s}^{-1} \cdot \mathrm{m}^{-2} \cdot 1} = 3941\ \mathrm{s} \sim 66\ \mathrm{min} \qquad (10)$$

Charging this device to 90%, where 10 mM of Ph3NB remain in solution thus requires 66 min in this simplified calculation. These estimations align well with the experimental solar irradiation times of 45-60 min observed in Chapter S9 for a setup with related conditions.

8. Additional spectroscopic measurements

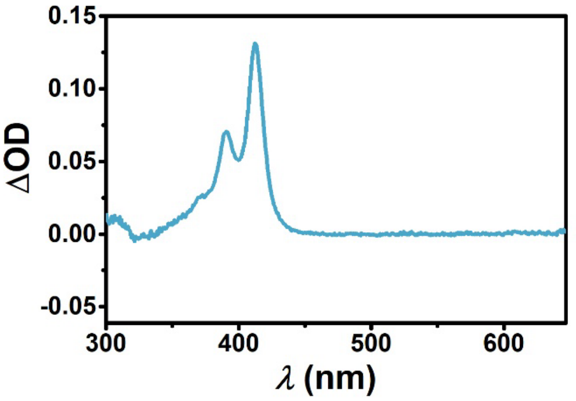

Figure S23: Triplet-triplet absorption spectrum of naphthalene. The spectrum was recorded 600 ns after 355 nm laser excitation using an Ar-saturated solution containing 40 µM thioxanthone and 1 mM naphthalene in MeCN.

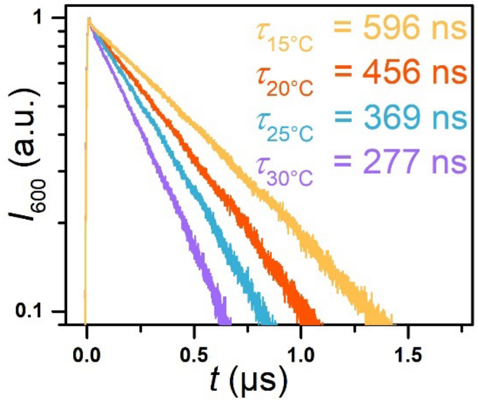

Figure S24: Time-resolved emission of 15 µM RuPhen in Ar-saturated MeCN recorded at 15°C, 20°C, 25°C and 30°C upon 532 nm excitation.

The temperature-dependent emission of RuPhen was measured at 15°C, 20°C, 25°C, and 30°C (Figure S24). As the temperature increased, the lifetime of RuPhen decreased significantly, from 596 ns at 15°C to 277 ns at 30°C. Despite using a temperature-controlled cuvette holder for LFP experiments, the actual solution temperature can vary due to external conditions (e.g., ambient temperature). Given the high temperature sensitivity of the lifetime, even minor fluctuations can cause changes of ± 25 ns. Nevertheless, at room temperature (20°C), the literature-reported lifetime of RuPhen[25] matches well with the measured range across all experiments (~450-510 ns). Therefore, the unquenched lifetime of RuPhen was determined individually for each experiment.

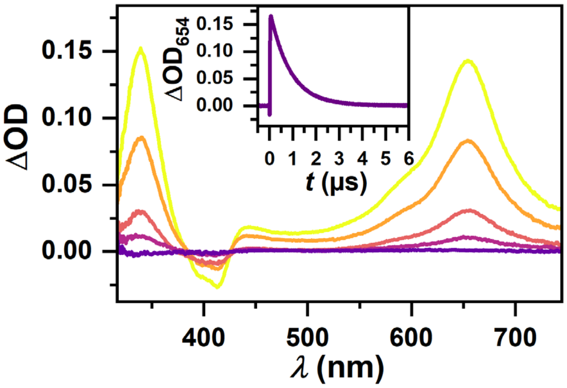

Figure S25: TA spectra of an Ar-saturated toluene solution containing 200 µM MeOTX and 500 µM Ph3NB using 355 nm laser pulses. The spectra were recorded 100 ns, 500 ns, 1 µs, 2 µs and 5 µs after excitation. Inset: Time-resolved transient absorption at 654 nm.

Transient absorption spectroscopy was used to investigate a solution of MeOTX and Ph3NB in toluene. Following the initial quenching of MeOTX via triplet energy transfer to Ph3NB, only the baseline level was observed. No quenching products from MeOTX, resulting from electron or hydrogen atom transfer, were detected, which allows us to exclude side reactions, in line with the ~100% switching quantum efficiency determined in Chapter S6.

8.1. Mechanistic investigations of the triplet state of 2NNB

Analogous to the measurements of the triplet species of 1NNB in the main paper, we investigated the structural isomer 2NNB as well. RuPhen was employed as sensitizer and high concentrations of 2NNB (36 mM) were used ensuring selective excitation and optimized conditions for the detection of short-lived intermediates (see main paper for details). A TA spectrum recorded 30 ns after 532 nm laser excitation in

Ar-saturated MeCN is displayed in Figure S26. A reference spectrum of RuPhen was scaled to match in the region >600 nm. The difference between both spectra reveals the transient triplet species of 2NNB.

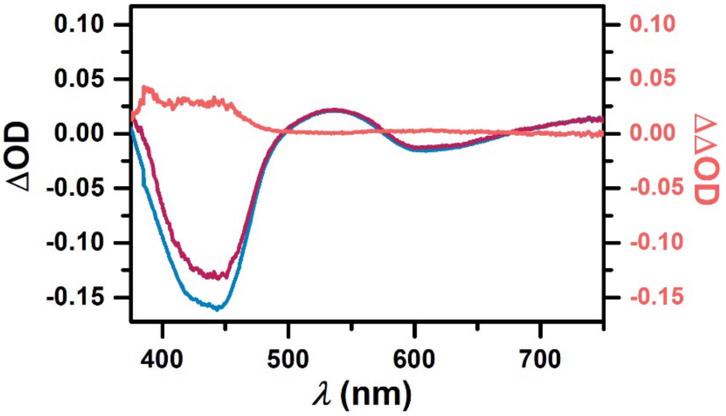

Figure S26: Mechanistic investigations of the triplet state of 2NNB by laser flash photolysis using 532 nm laser pulses. TA spectra of a solution of 100 µM RuPhen in the absence (blue) and presence (red) of 36 mM 2NNB in deaerated MeCN. The spectrum of RuPhen was scaled to match in the region >600 nm in order to determine the respective difference spectrum (orange).

Time-resolved measurements at the isosbestic point of RuPhen were not successful because of the weak absorption of ^{3}2NNB at 496 nm. To determine the triplet lifetime of 2NNB we compared the kinetic emission of RuPhen (at 600 nm, 39 ns lifetime) to the time-resolved transient absorption at 400 nm (superposition of the ground state bleach of RuPhen and absorption of ^{3}2NNB) (Figure S27). The RuPhen emission was appropriately scaled to match the minimum of the TA measurement. A lifetime analysis was unsuccessful due to the much shorter (< 20 ns) triplet lifetime of 2NNB.

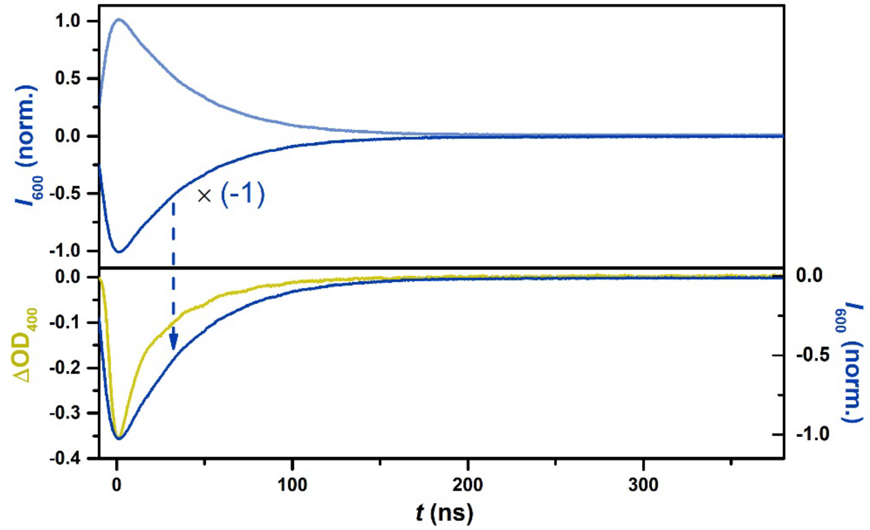

Figure S27: Time-resolved measurements of a solution of of 100 µM RuPhen and 36 mM 2NNB in deaerated MeCN upon 532 nm excitation. Blue traces display the kinetic emission at 600 nm. The dark yellow trace displays the kinetic transient absorption at 400 nm.

8.2. Steady-state absorption spectra

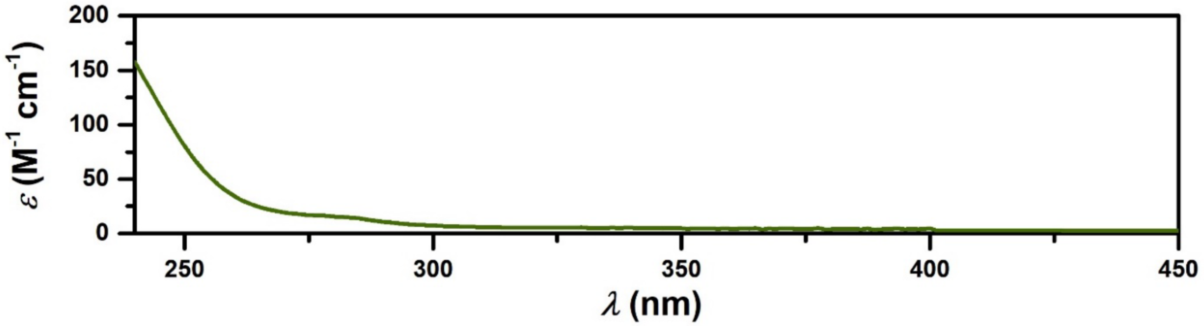

Figure S28: Molar absorption coefficient of NB (stabilized with dibutylhydroxytoluene) in MeCN plotted against the wavelength.

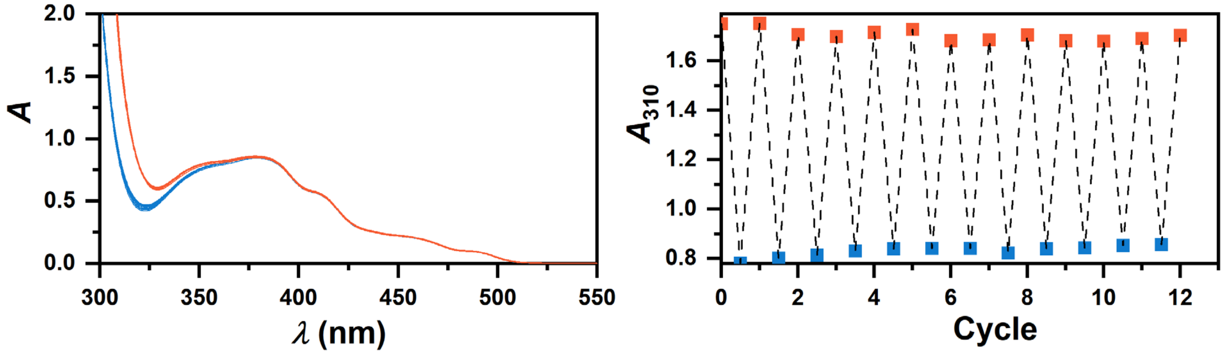

Figure S29: Right panel: Steady-state monitoring of multiple interconversions between Ph3NB and Ph3QC using Ir(ppy)$_3$ as sensitizer. Each isomerization cycle was performed by irradiation a solution of 300 µM Ph3NB with 50 µM Ir(ppy)$_3$ in Ar-saturated toluene at 440 nm for 4 min followed by heat treatment at 85°C for 1 h in a Schlenk cuvette. Left panel: Absorption changes at 310 nm displayed for 12 cycles.

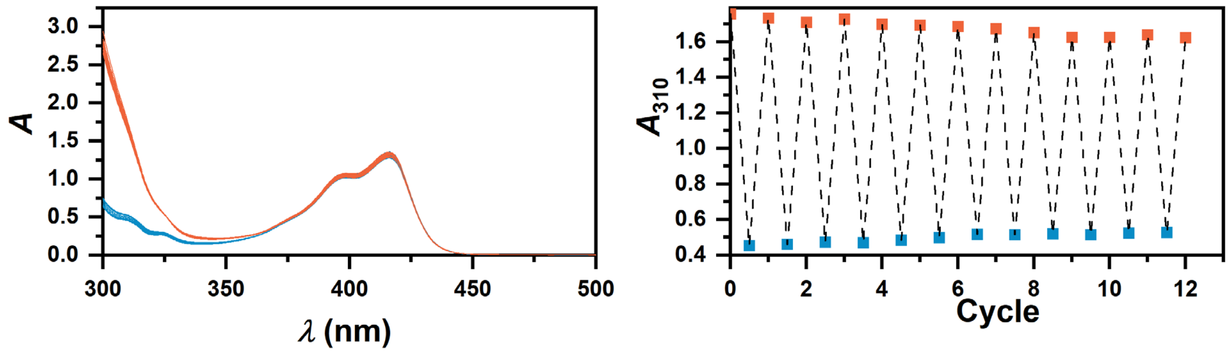

Figure S30: Right panel: Steady-state monitoring of multiple interconversions between Ph3NB and Ph3QC using MeOTX as sensitizer. Each isomerization cycle was performed by irradiation a solution of 400 µM Ph3NB with 245 µM MeOTX in Ar-saturated toluene at 440 nm for 4 min followed by heat treatment at 85°C for 1 h in a Schlenk cuvette. Left panel: Absorption changes at 310 nm displayed for 12 cycles.

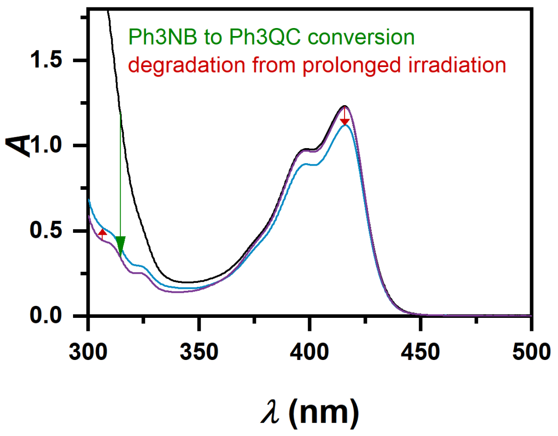

Figure S31: Steady-state absorption measurements of 245 µM MeOTX and 400 µM Ph3NB in Ar-saturated toluene. Absorption spectra were recorded before irradiation (black), after 4 min of photoirradiation using a 440 nm LED monitoring the Ph3NB to Ph3QC conversion (purple) and prolonged irradiation (another 4 min irradiation) leading to degradation of the sensitizer (blue).

A solution containing MeOTX and Ph3NB in Ar-saturated toluene was prepared and irradiated with a 440 nm LED (Figure S31). After 4 minutes of irradiation, the conversion to Ph3QC was complete (purple graph). Continued irradiation of the system resulted in the degradation of the sensitizer, as shown in the blue graph. This degradation is faster after the isomerization as a result of the unquenched triplet state of MeOTX.

8.3. Photostationary state composition of triplet sensitized 1NNB/1NQC

The photostationary state composition of triplet sensitized 1NNB/1NQC was investigated by steady-state and NMR spectroscopy in the presence of structurally similar triplet sensitizers. Thioxanthone (TX) and 2,7-dimethoxythioxanthone (MeOTX) exhibit triplet energies of 2.75 eV and 2.38 eV, respectively.[10] An adiabatic triplet state energy of 2.51 eV was estimated for 1NQC by DFT calculations (see Chapter S3), which should be accessible only by the sensitizer with higher triplet energy, TX, while 1NNB is sensitized by both TX and MeOTX.

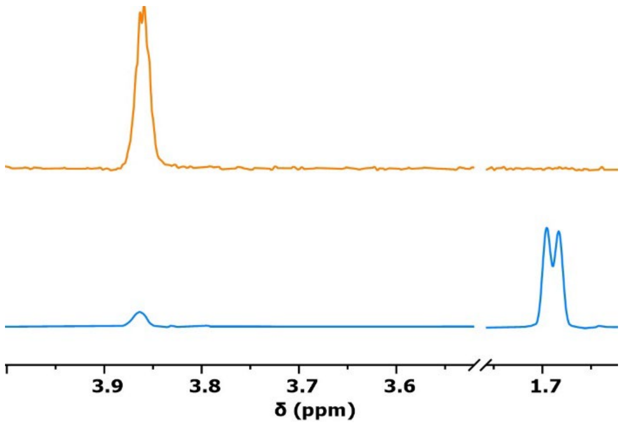

Figure S32: NMR spectra of 3 mM 1NNB and 10 mol% TX in degassed MeCN-d_3, before (top) and after (bottom) irradiation with a Thorlabs 405 nm LED (1 cm distance, 100%) for 1 h. A cut-off filter at 385 nm was used to avoid direct excitation of 1NNB.

An NMR irradiation experiment was conducted with 3 mM 1NNB and 10 mol% TX in Ar-saturated MeCN-d3, irradiating at 405 nm for 1 hour, resulting in a ratio of 7/93 (1NNB/1NQC) (Figure S32). Analogous irradiation experiments monitored by steady-state absorption at different irradiation time intervals of 150 µM 1NNB with either sensitizer confirm that a photostationary state composition is reached rapidly (Figure S33 and S34).

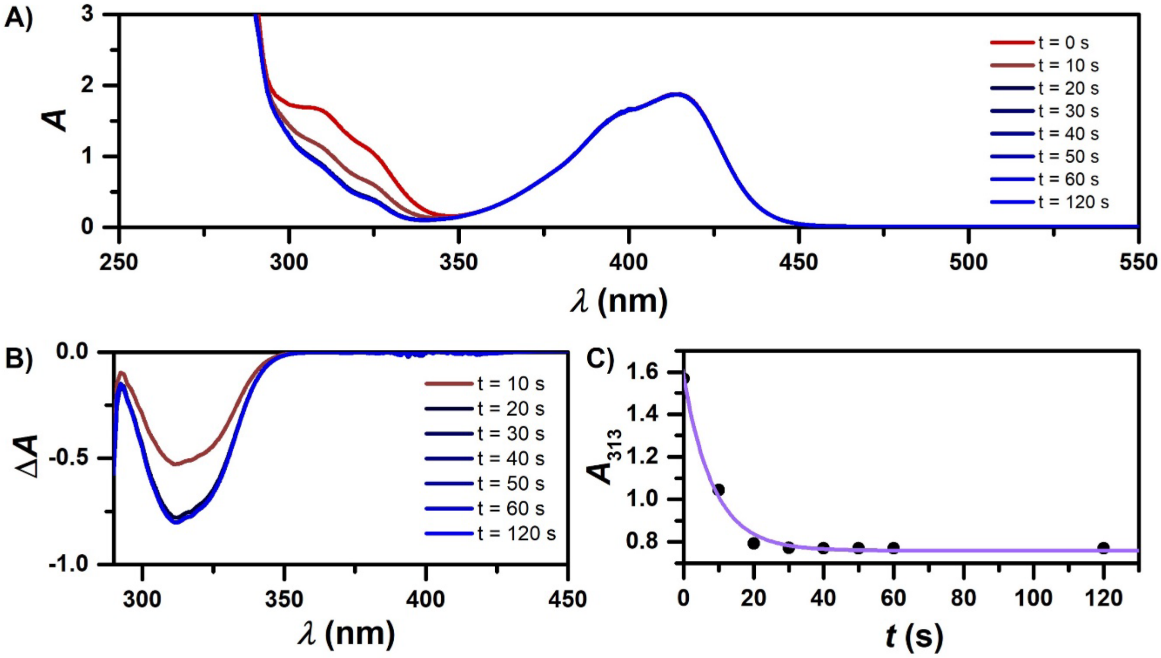

Figure S33: Steady-state absorption measurements of 385 µM MeOTX and 150 µM 1NNB in Ar-saturated MeCN irradiated with a 440 nm LED (25% and 10 cm distance). A) Absorption spectra recorded at different irradiation time intervals. B) Corresponding difference absorption. C) Absorption at 313 nm plotted against the irradiation time, fitted using an exponential decay function.

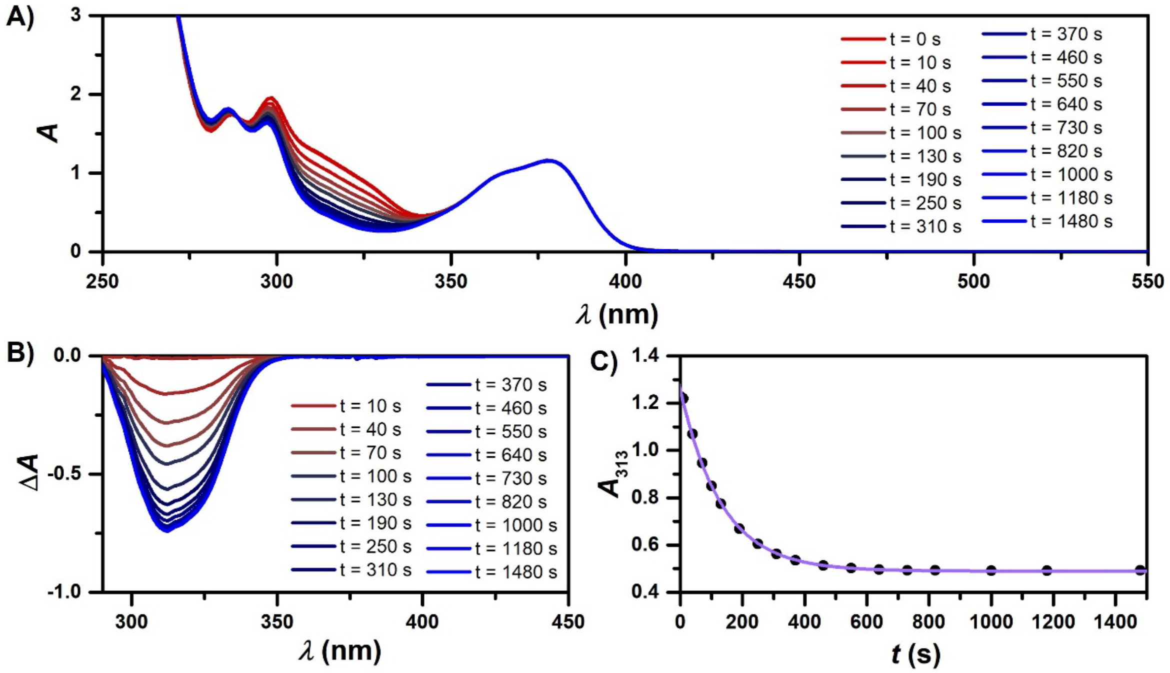

Figure S34: Steady-state measurements of 235 µM TX and 150 µM 1NNB in Ar-saturated MeCN irradiated with a 405 nm LED (100% and 1 cm distance). A) Absorption spectra recorded at different irradiation time intervals. B) Corresponding difference absorption spectra. C) Absorption at 313 nm plotted against the irradiation time, fitted using an exponential decay function.

Quantitative interconversion is achieved when MeOTX is employed as sensitizer (see Figure S40), we compared the relative changes in the absorption spectrum to estimate a ratio of 8/92 of 1NNB/1NQC at the photostationary state in the presence of TX (Figure S34), reflecting the results obtained by ^{1}H NMR spectroscopy. Further LFP experiments using 355 nm laser pulses for the excitation of 60 µM TX with 271 µM 1NNB in Ar-saturated MeCN were recorded during 440 nm LED irradiation (Figure S35B). The triplet state of TX is not luminescent, hence time-resolved transient absorption spectroscopy at 625 nm was used to monitor the quenching step by 1NNB/1NQC.[10] Concurrent steady-state absorption experiments confirm that the photostationary state was reached after 4 min of irradiation (Figure S35A). The lifetime of TX was virtually unchanged (346 ns at t = 0 min to 351 ns at t = 4 min) during irradiation suggesting that 1NNB and 1NQC quench TX with nearly identical rate constants, which would imply a ~50:50 ratio of 1NNB and 1NQC in the photostationary state. While quenching by 1NQC is feasible when a high triplet energy sensitizer such as TX is employed, the inherent switching efficiency of ^{3}1NQC to 1NNB seems to be low based on the results in Figure S32 (7:93 ratio). These results underline the very high interconversion efficiency of ^{3}1NNB to 1NQC obtained by relative actinometry (see Chapter S5).

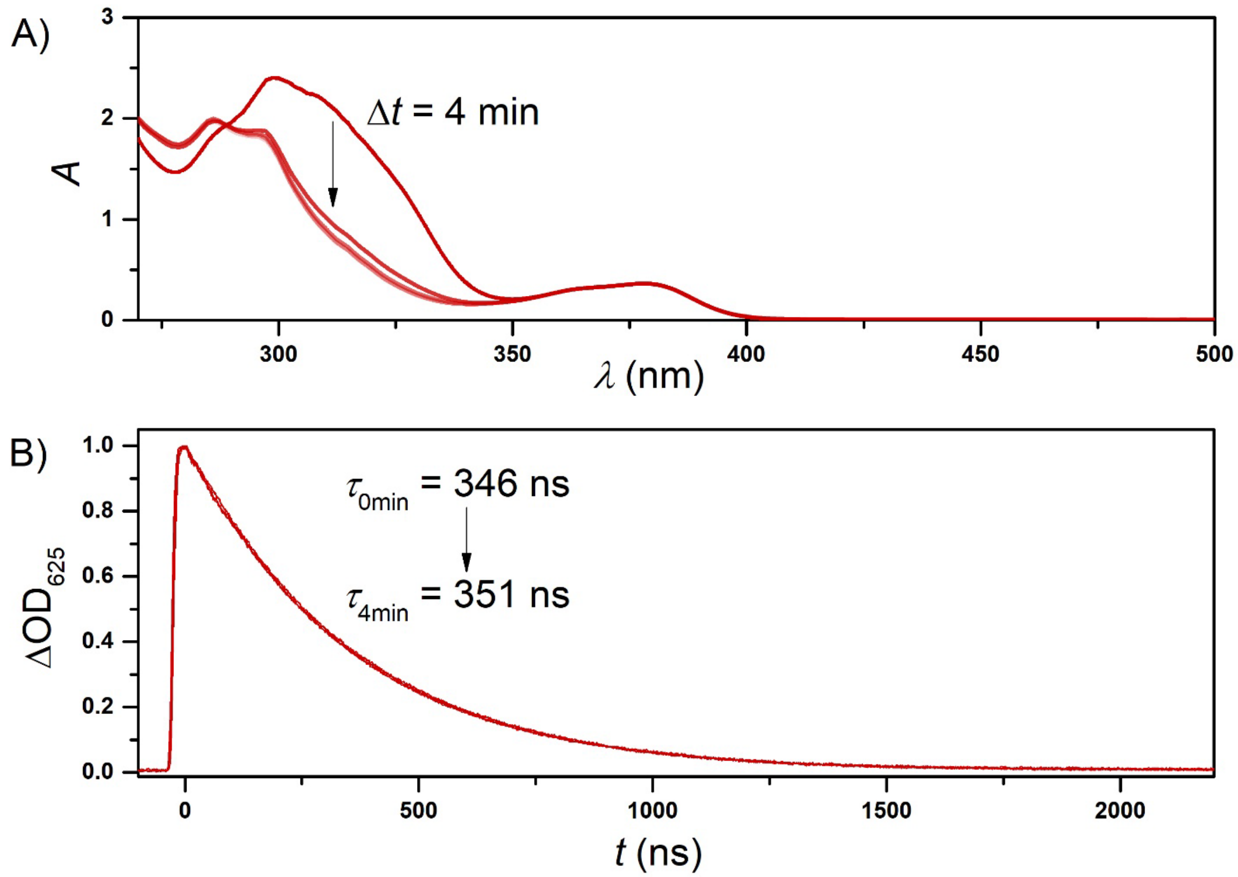

Figure S35: Mechanistic investigations of the isomerization of 271 µM 1NNB using 60 µM TX as triplet sensitizer in Ar-saturated MeCN. The solution was irradiated for 4 minutes with a 405 nm LED from Thorlabs. Measurements were taken within 1 minute intervals. A) Absorption spectra. B) Time-resolved transient absorption with 355 nm laser pulses detected at 625 nm.

9. Multiple conversion cycles of the Ir(ppy)$_3$–Ph3NB pair under solar irradiance

A solution of 100 mM Ph3NB with 590 µM Ir(ppy)$_3$ (0.59 mol%) and 26.9 mM bibenzyl (used as standard) in Ar-saturated deuterated toluene was prepared by three freeze-pump-thaw cycles and sealed in an NMR tube. The solution was exposed to sunlight for 45 min to 60 min. A 400 nm cut-off filter was used to avoid direct excitation of Ph3NB. The experimental setup is shown in Figure S36. The NMR tube was then heated to 85°C for 1 h recovering Ph3NB. This cycle was repeated five times over three days (27.08.2024-29.08.2024). After each step an NMR spectrum is recorded ensuring complete conversion to Ph3QC or Ph3NB (Figure S47). Bibenzyl served as the NMR standard allowing us to quantify the isomers. The solar irradiation times and dates, heating times and concentrations are displayed in Table S4.

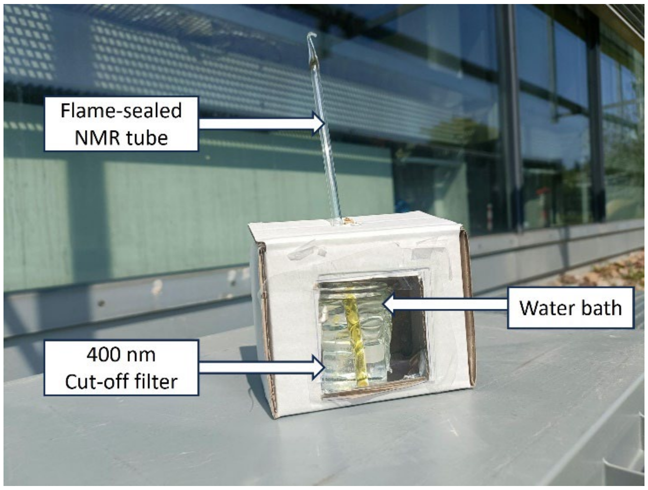

Figure S36: Experimental setup for the solar irradiation experiments of a solution of 100 mM Ph3NB with 590 µM Ir(ppy)$_3$ (0.59 mol%) and 26.9 mM bibenzyl in Ar-saturated deuterated toluene.

Table S4: Irradiation times, heating times, and concentrations of Ph3NB and Ph3QC across multiple conversion cycles of Ir(ppy)$_3$-Ph3NB. See Figure S47 for raw data sets.

Cycle	Irradiation time (min)[a]	Heating time (min)	Concentration (mM)[b]
0	–	–	100.0
0.5	60 min (16:45-17:45, 27.08.2024)	–	100.4
1	–	60 min	101.7
1.5	45 min (11:30-12:15, 28.08.2024)	–	98.4
2	–	60 min	98.2
2.5	45 min (15:30-16:15, 28.08.2024)	–	95.1
3	–	60 min	98.1
3.5	45 min (10:30-11:15, 29.08.2024)	–	98.1
4	–	60 min	97.2
4.5	45 min (14:00-14:45, 29.08.2024)	–	95.8
5	–	60 min	98.9

[a] No cloud coverage during irradiation. [b] Concentration of Ph3NB or Ph3QC determined by quantitative ^{1}H NMR spectroscopy.

10. NMR spectra

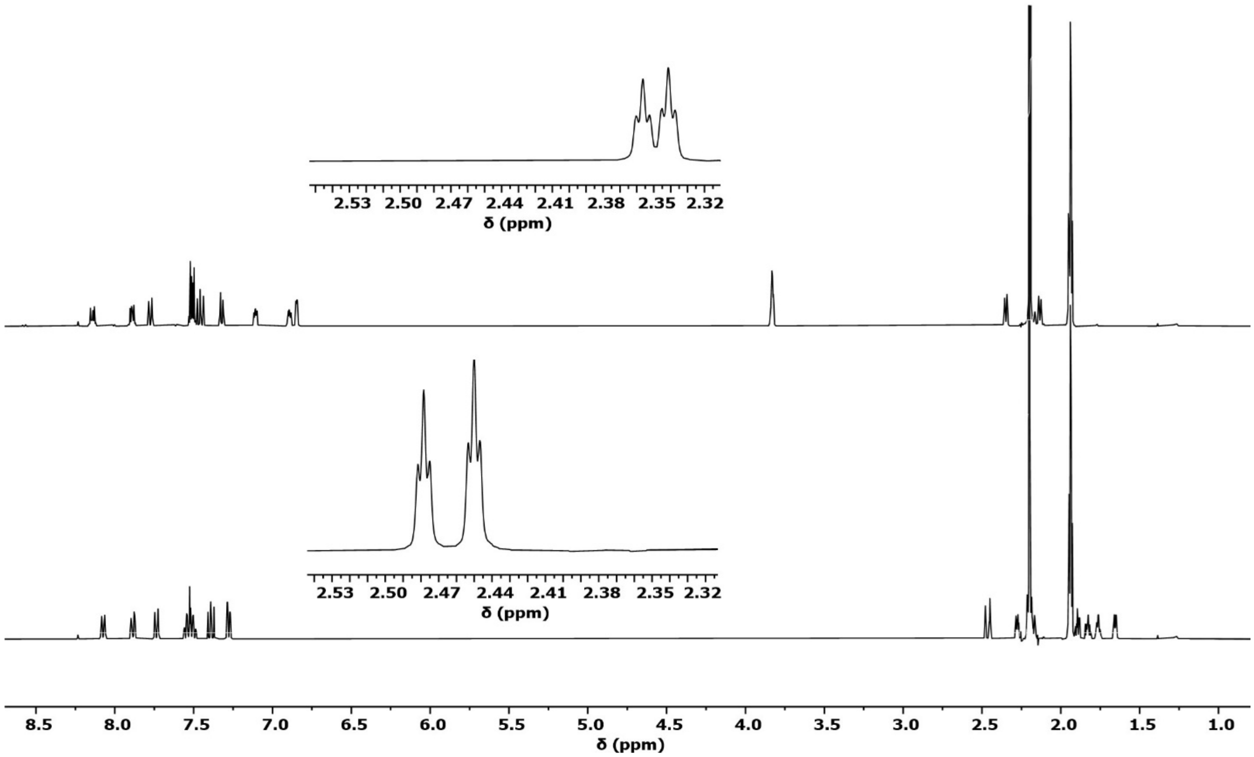

Figure S37: ^{1}H NMR spectra of a solution containing 30 mM 1NNB and 0.5 mol% RuPhen before (top) and after (bottom) 525 nm irradiation for 30 min in deuterated MeCN.

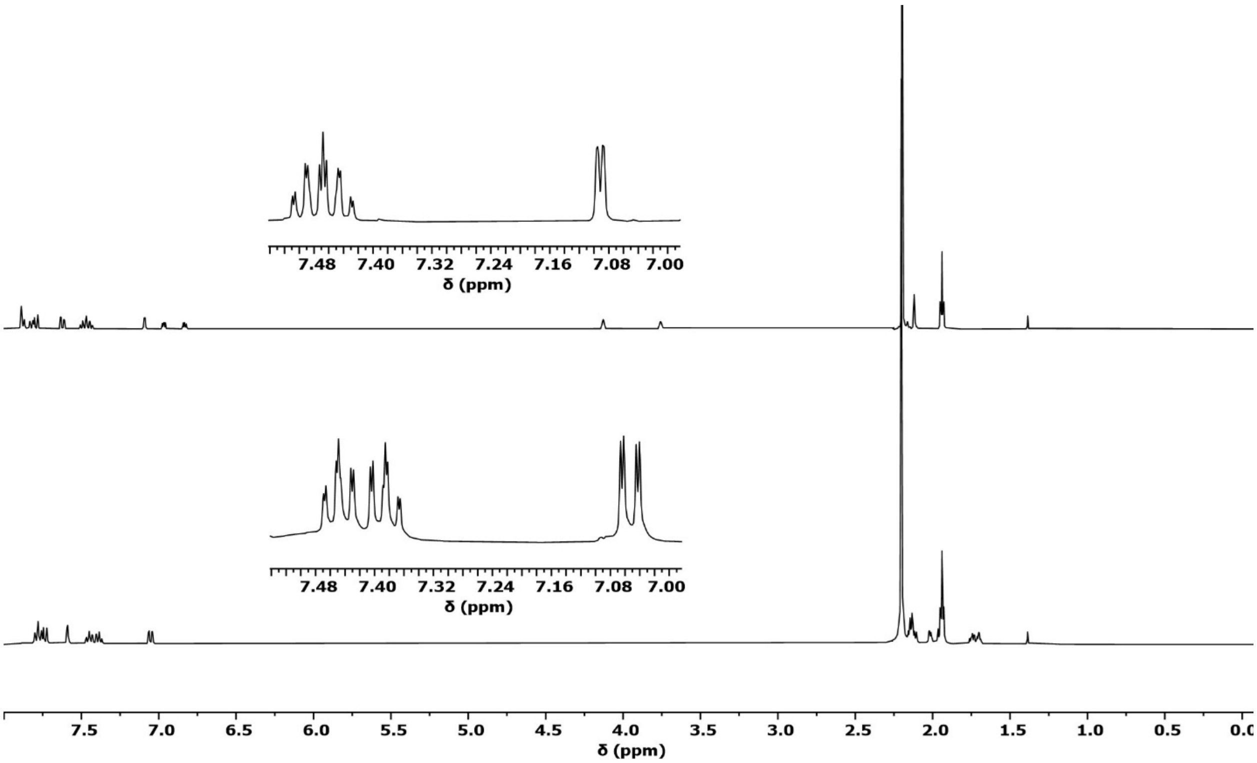

Figure S38: ^{1}H NMR spectra of a solution containing 36 mM 2NNB and 0.5 mol% RuPhen before (top) and after (bottom) 525 nm irradiation for 21 min in deuterated MeCN.

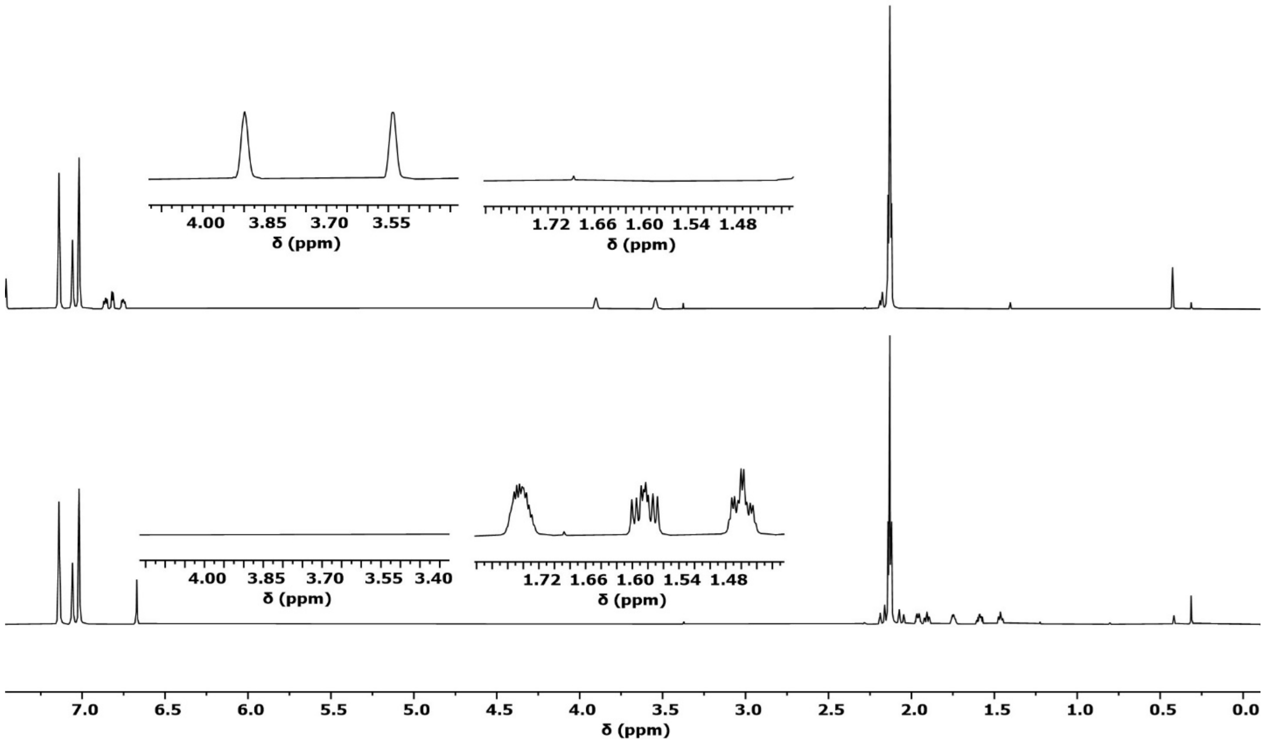

Figure S39: ^{1}H NMR spectra of a solution containing 5 mM Ph3NB and 5 mol% MeOTX before (top) and after (bottom) 440 nm irradiation for 30 min in deuterated toluene.

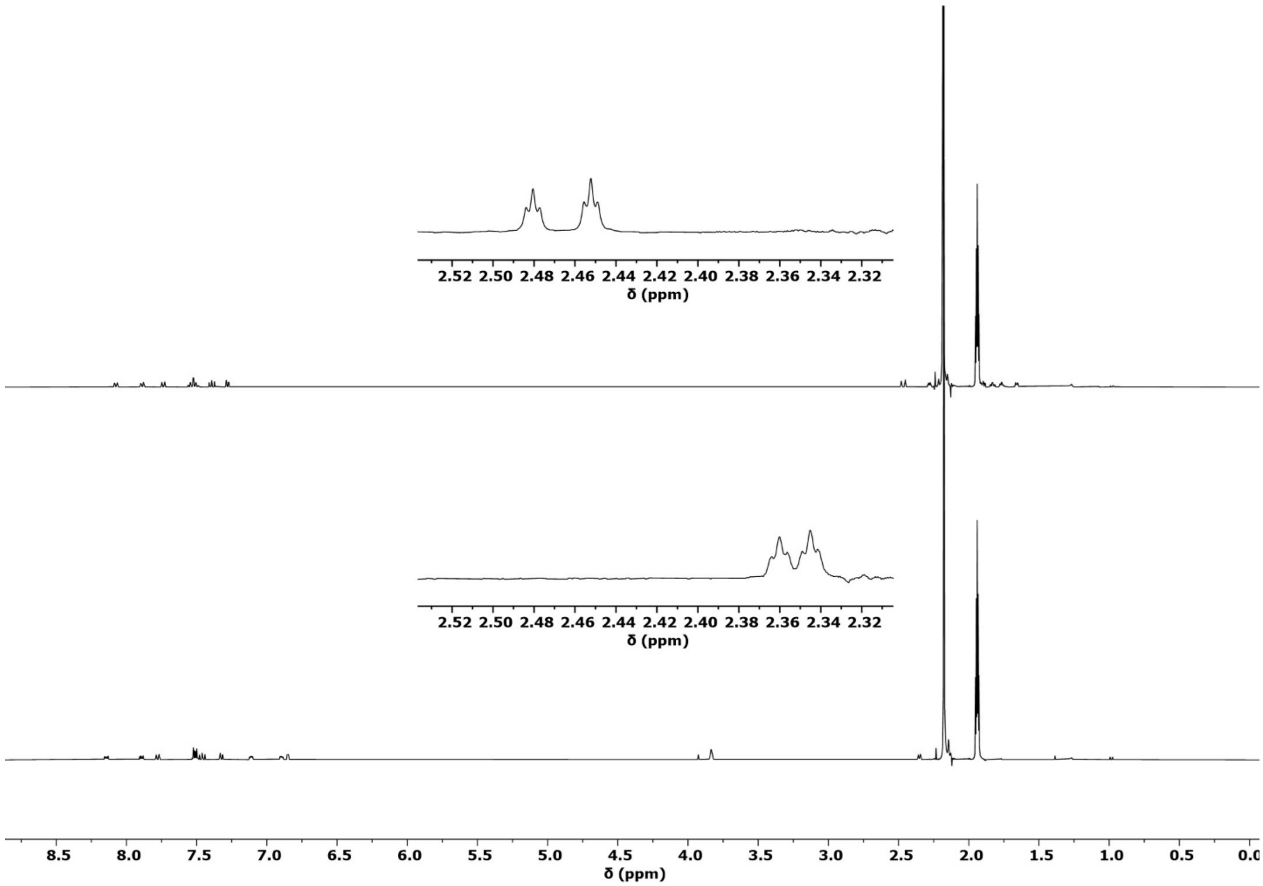

Figure S40: ^{1}H NMR spectra of a solution containing 5 mM 1NNB and 5 mol% MeOTX before (top) and after (bottom) 440 nm irradiation for 30 min in MeCN-d_3.

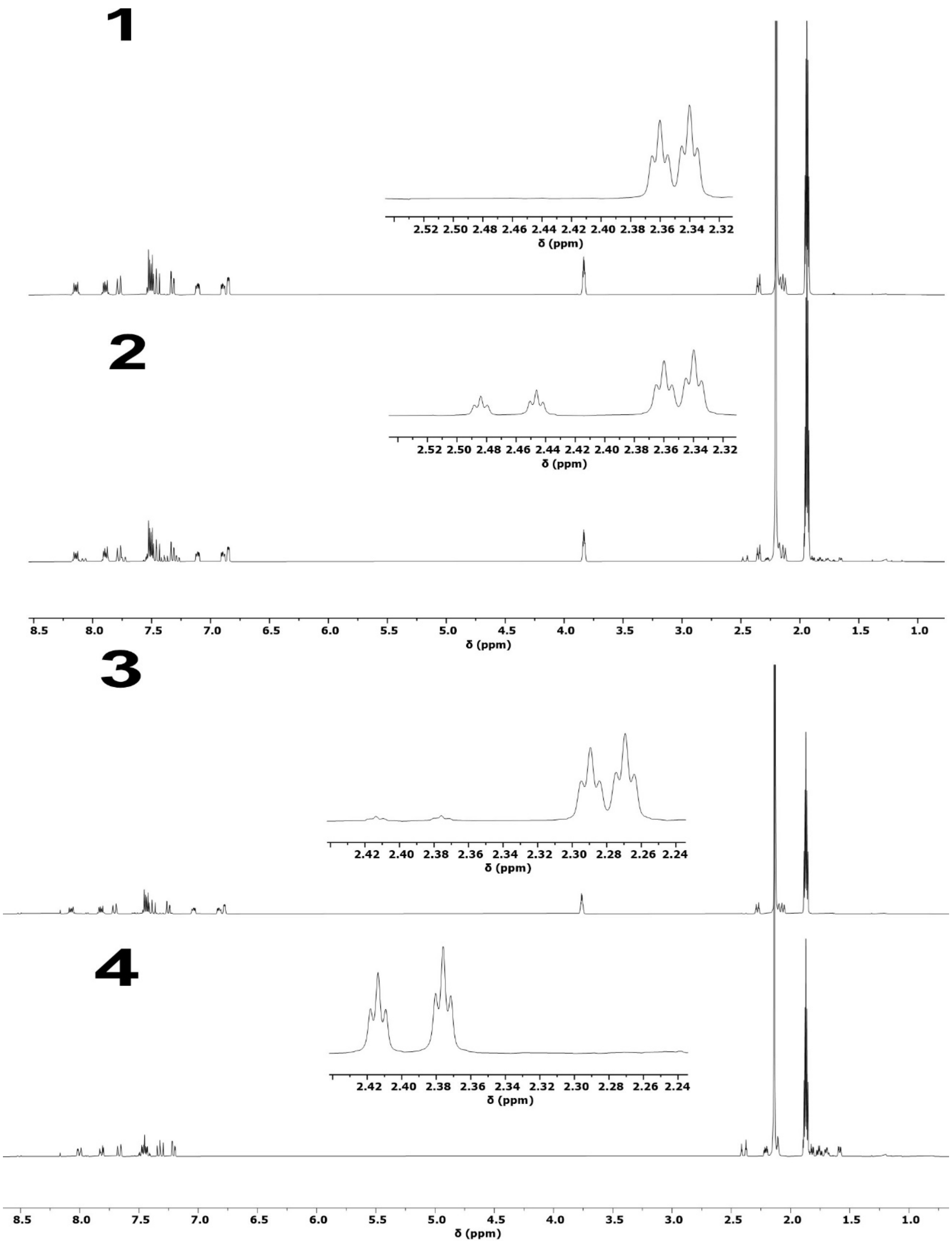

Figure S41: ^{1}H NMR experiment of 22.5 mM 1NNB in Ar-saturated MeCN-d_3 before (1) and after (2) solar irradiation. ^{1}H NMR experiment of 0.25 mM $[Ru(phen)_3](PF_6)_2$ with 22.5 mM 1NNB in Ar-saturated MeCN-d_3 before (3) and after (4) solar irradiation (date: 06.09.2022, time: 2.00 pm – 3.30 pm , 21-22 °C). For experimental setup see Figure S42.

.

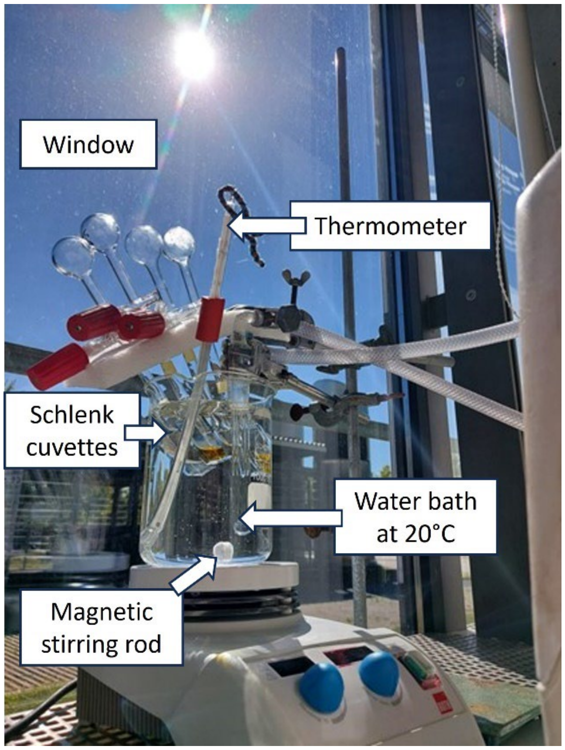

Figure S42: Experimental setup for solar irradiation experiment (corresponding ^{1}H NMR data sets can be found in Figure S41).

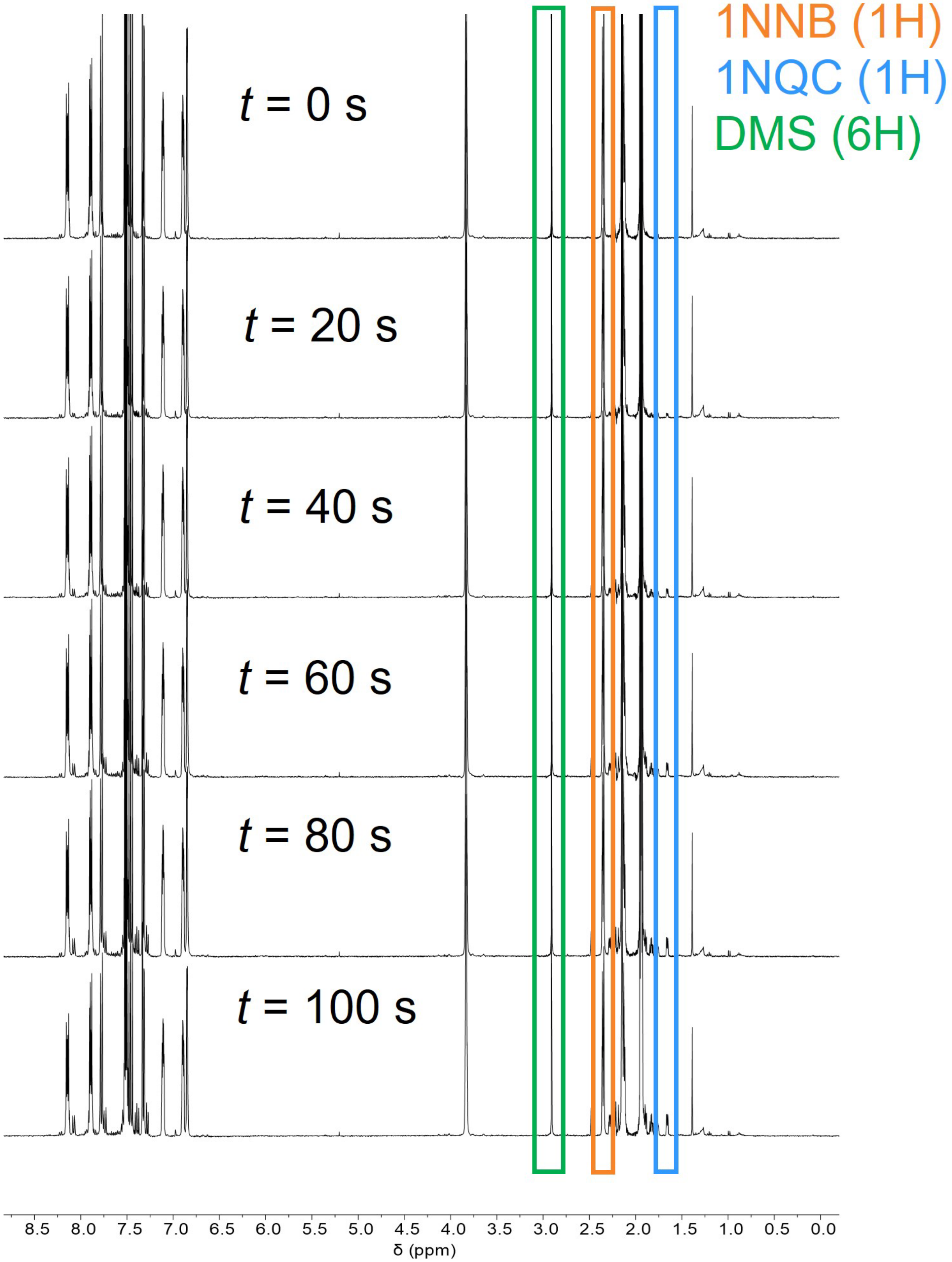

Figure S43: ^{1}H NMR spectra of an Ar-saturated solution containing 23.4 µM $Ir(ppy)_3$, 29.1 mM 1NNB, and 2.53 mM DMS. The spectra were recorded during irradiation with a 440 nm LED (set to 25%, 8 cm distance) with intervals of 20 s. See Chapter S5 for details.

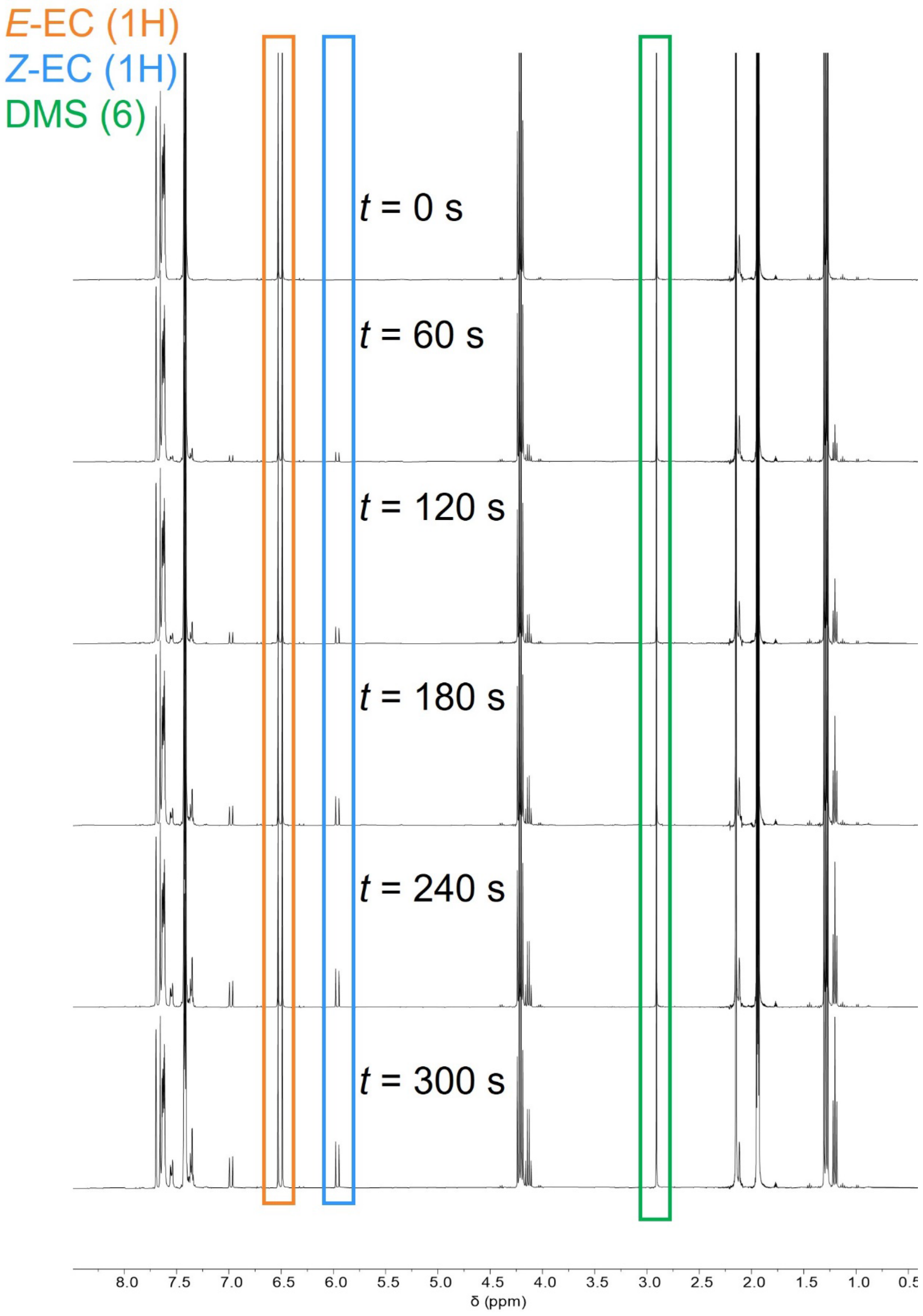

Figure S44: ^{1}H NMR spectra of an Ar-saturated solution containing 23.4 µM $Ir(ppy)_3$, 30.5 mM *E*-EC, and 2.53 mM DMS. The spectra were recorded during irradiation with a 440 nm LED (set to 25%, 8 cm distance) with intervals of 60 s. See Chapter S5 for details.

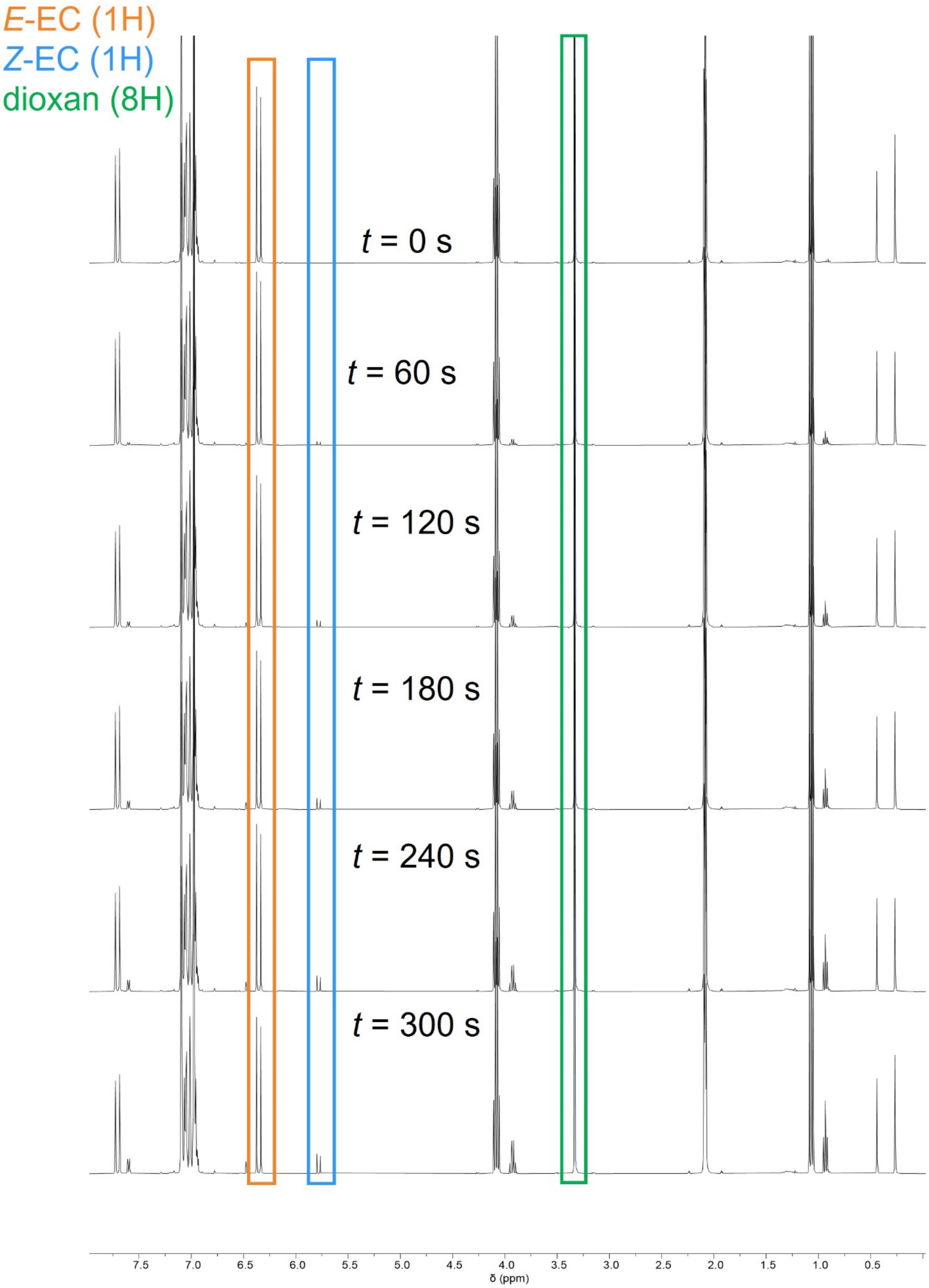

Figure S45: ^{1}H NMR spectra of an Ar-saturated toluene-d_8 solution containing 23.0 µM $Ir(ppy)_3$, 30.0 mM *E*-EC, and 10.0 mM dioxan. The spectra were recorded during irradiation with a 440 nm LED (set to 25%, 8 cm distance) with intervals of 60 s. See Chapter S6 for details.

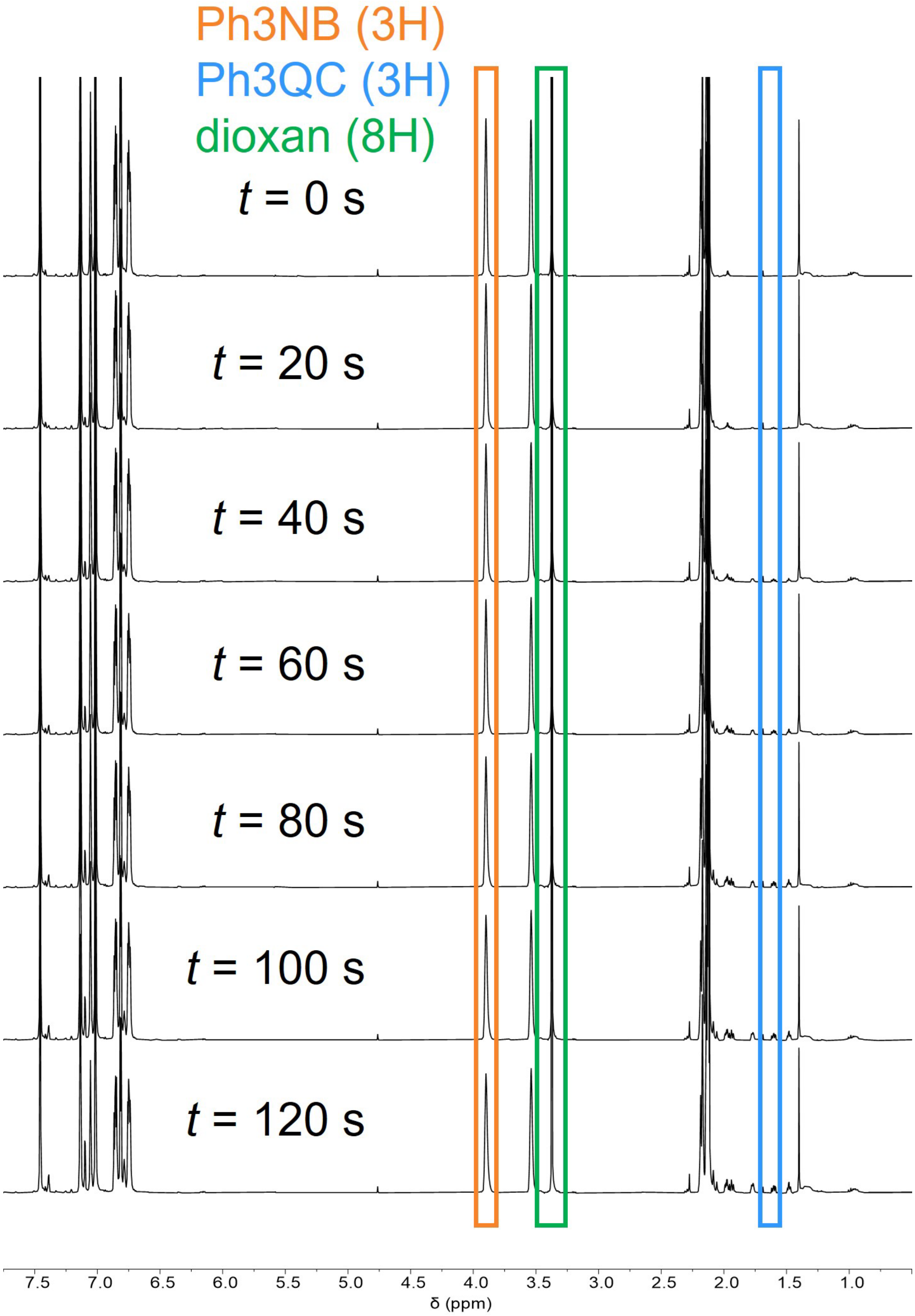

Figure S46: ^{1}H NMR spectra of an Ar-saturated toluene-d_8 solution containing 23 µM $Ir(ppy)_3$, 20 mM Ph3NB, and 10.0 mM dioxan. The spectra were recorded during irradiation with a 440 nm LED (set to 25%, 8 cm distance) with intervals of 20 s. See Chapter S6 for details.

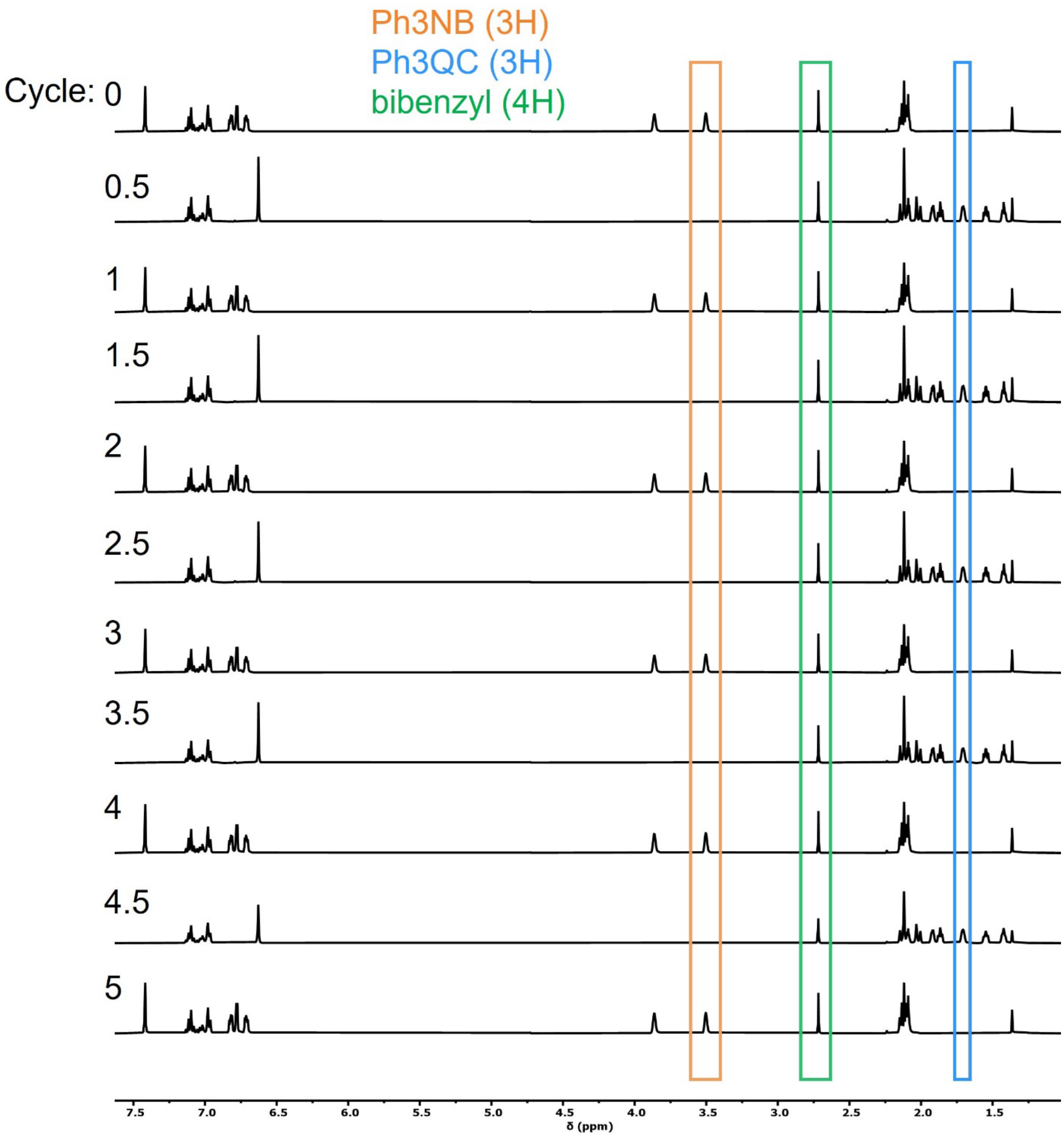

Figure S47: ^{1}H NMR spectra of an Ar-saturated toluene-d_8 solution containing 590 µM Ir(ppy)$_3$, 100 mM Ph3NB, and 26.9 mM bibenzyl. The spectra were recorded for five conversion cycles. See Chapter S9 for details.

11. Literature

[1] T. J. B. Zähringer, M.-S. Bertrams, C. Kerzig, *J. Mater. Chem. C* **2022**, *10*, 4568–4573.
[2] E. C. Constable, P. R. Raithby, D. N. Smit, *Polyhedron* **1989**, *8*, 367–369.
[3] M. S. Lowry, W. R. Hudson, R. A. Pascal, S. Bernhard, *J. Am. Chem. Soc.* **2004**, *126*, 14129–14135.
[4] R. Schulte, H. Ihmels, *Beilstein J. Org. Chem.* **2022**, *18*, 368–373.
[5] R. Schulte, S. Afflerbach, H. Ihmels, *Eur. J. Org. Chem.* **2023**, *26*, e202201398.

[6] R. Schulte, S. Afflerbach, T. Paululat, H. Ihmels, *Angew. Chem. Int. Ed.* **2023**, *62*, e202309544.
[7] O. Baumgärtel, G. Szeimies, *Chem. Ber.* **1983**, *116*, 2180–2204.
[8] F. Neese, *WIREs Comput. Mol. Sci.* **2012**, *2*, 73–78.
[9] M. D. Hanwell, D. E. Curtis, D. C. Lonie, T. Vandermeersch, E. Zurek, G. R. Hutchison, *J. Cheminformatics* **2012**, *4*, 17.
[10] T. J. B. Zähringer, M. Wienhold, R. Gilmour, C. Kerzig, *J. Am. Chem. Soc.* **2023**, *145*, 21576–21586.
[11] A. A. Vlcek, E. S. Dodsworth, W. J. Pietro, A. B. P. Lever, *Inorg. Chem.* **1995**, *34*, 1906–1913.
[12] Y. Wei, Y. Li, Z. Li, X. Xu, X. Cao, X. Zhou, C. Yang, *Inorg. Chem.* **2021**, *60*, 19001–19008.
[13] V. Balzani, F. Bolletta, F. Scandola, *J. Am. Chem. Soc.* **1980**, *102*, 2152–2163.
[14] A. M. Helms, Richard. A. Caldwell, *J. Am. Chem. Soc.* **1995**, *117*, 358–361.
[15] I. Antol, *J. Comput. Chem.* **2013**, *34*, 1439–1445.
[16] K. Raghavachari, R. C. Haddon, H. D. Roth, *J. Am. Chem. Soc.* **1983**, *105*, 3110–3114.
[17] M. Montalti, A. Credi, L. Prodi, M. T. Gandolfi, *Handbook of Photochemistry*, CRC Press, **2006**.
[18] Y. Shindo, K. Horie, I. Mita, *J. Photochem.* **1984**, *26*, 185–192.
[19] N. J. Turro, W. R. Cherry, M. F. Mirbach, M. J. Mirbach, *J. Am. Chem. Soc.* **1977**, *99*, 7388–7390.
[20] K. Nakabayashi, H. Nishino, S. Toki, S. Takamuku, *Int. J. Radiat. Appl. Instrum. Part C Radiat. Phys. Chem.* **1989**, *34*, 809–815.
[21] Z. Wang, J. Udmark, K. Börjesson, R. Rodrigues, A. Roffey, M. Abrahamsson, M. B. Nielsen, K. Moth-Poulsen, *ChemSusChem* **2017**, *10*, 3049–3055.
[22] Z. Wang, A. Roffey, R. Losantos, A. Lennartson, M. Jevric, A. U. Petersen, M. Quant, A. Dreos, X. Wen, D. Sampedro, K. Börjesson, K. Moth-Poulsen, *Energy Environ. Sci.* **2019**, *12*, 187–193.
[23] "The American Society for Testing and Materials (ASTM) G-173, 'Reference Air Mass 1.5 Spectra,'" can be found under https://www.nrel.gov/grid/solar-resource/spectra.html, **2003**.
[24] T. Hofbeck, H. Yersin, *Inorg. Chem.* **2010**, *49*, 9290–9299.
[25] A. Juris, V. Balzani, F. Barigelletti, S. Campagna, P. Belser, A. Von Zelewsky, *Coord. Chem. Rev.* **1988**, *84*, 85–277.
